# Structural Features and Photophysical and Antiproliferative Properties of Me_2_N‑pbt-Cycloplatinated Complexes with Picolinate Ligands

**DOI:** 10.1021/acs.inorgchem.5c01175

**Published:** 2025-05-13

**Authors:** David Gómez de Segura, Michèle Salmain, Benoît Bertrand, Julio Fernández-Cestau, Elena Lalinde, M. Teresa Moreno

**Affiliations:** † Departamento de Química-Instituto de Investigación en Química (IQUR), 16764 Universidad de La Rioja, 26006 Logroño, Spain; ‡ 27063 Sorbonne Université, CNRS, Institut Parisien de Chimie Moléculaire (IPCM UMR 8232), F-75005 Paris, France

## Abstract

2-(4-dimethylaminophenyl)­benzothiazolate (Me_2_N-pbt)-cyclometalated platinum complexes containing four different picolinate ligands, [Pt­(Me_2_N-pbt)­(R-pic-κ*N∧O*)] (R = 3-H **1**, 3-NH_2_ **2**, 3-OH **3**, 4-COOH **4**) were prepared and examined for their photophysical properties, singlet oxygen production, and bioactivity. X-ray studies of **1** and **3**·CHCl_3_ revealed aggregation to give 1D infinite chains. They showed phosphorescent emissions essentially associated with metal-perturbed ^3^ILCT excited states in **1**–**3** and mixed ^3^LL′CT/^3^ILCT (L = Me_2_N-pbt, L′ = pic) in **4**, in agreement with theoretical calculations. Their tendency to self-assemble was demonstrated in films (**1**, **3**) and DMSO/H_2_O (**3**). Complexes **1**–**3** showed singlet oxygen photosensitization quantum yields (ϕ_Δ_^1^O_2_) in the range of 13–17%. The *in vitro* biological activity toward selected cell lines in dark conditions and under 5 min irradiation at 450 nm was tested. Complex **3** showed the highest phototoxicity with up to 10 times improvement of the antiproliferative activity upon irradiation, with EC_50_ values in the nanomolar range, related to overproduction of ROS in dark conditions, further enhanced upon irradiation. Complexes **1**–**4** did not bind to DNA, while the most potent complex **3** demonstrated interaction with BSA and photooxidation of NADH. Finally, intracellular dose-dependent ROS production in MDA-MB-231 cells treated with **3** was observed in the dark and further stimulated upon blue light irradiation.

## Graphical abstract

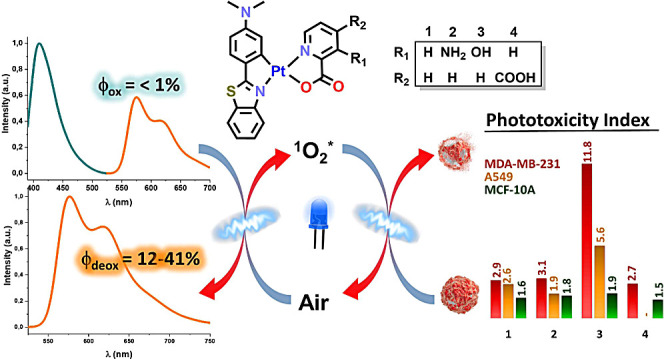

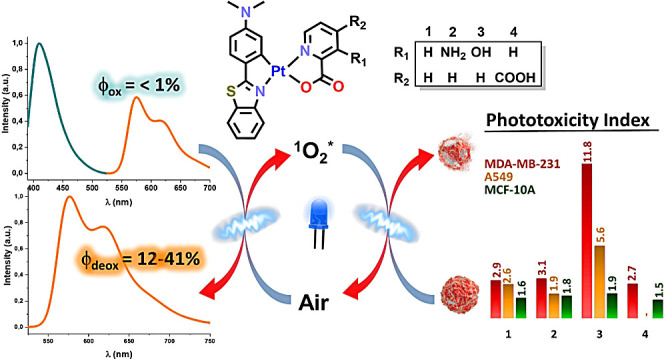

## Introduction

Since the discovery of the anticancer activity of cisplatin,^1^ extensive research has been carried out in order to develop more efficient metallodrugs with reduced side effects. However, only two platinum-based complexes (carboplatin and oxaliplatin), together with cisplatin, are used in clinics for the treatment of several types of cancer with worldwide approval.^2^

Cyclometalated platinum­(II) compounds have proved particularly promising as anticancer agents.^3^ These complexes present several advantages: (i) their high stability may allow them to reach the cells unaltered, (ii) the aromatic groups in the cyclometalated ligand might favor intercalative binding to DNA through π···π stacking, (iii) the presence of σ­(Pt–C) bonds increases the lability of the ligand in *trans*, favoring the coordination to DNA, as for cisplatin, and (iv) there is a wide range of cyclometalated and ancillary ligands, enabling optimization of their biological properties. Moreover, cycloplatinated­(II) complexes usually display luminescence properties^4^ with long-lived excited states, tunable ligands, high quantum yields, and cellular uptake.^3a,3b,3d,5^ As a consequence of these properties, cyclometalated complexes with long-lived excited states are of interest in further applications in bioimaging and biosensing^4a,6^ and they have emerged as excellent photosensitizers (PSs) for the production of reactive oxygen species (ROS).^7^ Upon illumination, the PS is promoted into a triplet excited state (^3^PS*) that transfers to the O_2_ molecules an electron (mechanism type I to produce free radicals or radical ions) or energy (type II to convert into singlet oxygen ^1^O_2_). Excess ROS production can induce a plethora of damaging effects on cellular components.^8^ PS cycloplatinated complexes generally produce singlet oxygen ^1^O_2_ via energy transfer from their triplet excited state to the triplet ground state of surrounding dioxygen (^3^O_2_) molecules,^9^ with high oxygen quantum yields (ϕ^1^O_2_). This effect is known as photodynamic effect and has numerous applications in photocatalysis^10^ and photodynamic therapy (PDT).^7,11^ The rate of ^1^O_2_ formation (*K*_*r*_^1^O_2_) is strongly dictated by the excited state lifetimes (τ) and the triplet state energies of the PS. The emission of singlet oxygen appears at 1270 nm and can be directly detected with a near-infrared (NIR) detector.^12^ The absolute quantum yield (ϕ^1^O_2_) and lifetime data are essential for an accurate determination of the photophysical data of ^1^O_2_. In this area, the selection of cyclometalated and auxiliary ligands is key to modulate these parameters and improve their selectivity and efficacy, which could significantly contribute to ongoing efforts to enhance the arsenal of cancer treatment options.

In this study, we employed substituted picolinate auxiliary ligands, which act as chelating ligands, comprising a six-membered ring with nitrogen and carboxyl groups. Picolinic acid is a metabolic product of the kynurenine pathway, contributing to the production of nicotinamide adenine dinucleotide (NAD^+^), which is essential for living cells.^13^ Despite the lack of clearly defined physiological functions, picolinic acid has been detected in various biological media, including cell culture supernatants, blood serum, cerebrospinal fluid, human milk, pancreatic juice, and intestinal homogenates.^14^ Some reports have described studies of picolinate transition metal complexes of Re­(I),^15^ Ir­(III),^16^ Os­(II),^17^ Ru­(II),^18^ or Pt­(II)^19^ toward different cancer cells.

Furthermore, we used 2-(4-dimethylaminophenyl)­benzothiazolate (Me_2_N-pbt) as a cyclometalated ligand. Benzothiazole is a core structure in various natural compounds and small organic molecules that are widely utilized in medicinal chemistry. Notably, 2-arylbenzothiazoles have shown significant antitumor activity^20^ and have been explored for their potential as amyloid-binding biomarkers.^21^ Among them, the donor–acceptor molecule, 2-(4-dimethylaminophenyl)­benzothiazole (Me_2_N-pbtH), stands out due to its intriguing photophysical characteristics,^22^ and 2-aminobenzothiazoles have been explored to construct molecules with excellent biological activity.^23^ However, studies related to the biological activity of cyclometalated complexes derived from this ligand are scarce.^24^ We have previously reported the synthesis, photophysical properties, and bioactivity studies of two related families, [Pt­(Me_2_N-pbt)­XL] [X = Cl, L = DMSO, 1,3,5-triaza-7-phosphaadamantane (PTA);^24a^ X = C_6_F_5_, L = 4-(diphenylphosphino)­benzoic acid (*p*-dpbH), 2-(diphenylphosphino)­benzoic acid (*o*-dpbH)], [Pt­(Me_2_N-pbt)­(*o*-dpb)] and the binuclear [{Pt­(Me_2_N-pbt)­(C_6_F_5_)}_2_(μ-PR_12_P)] (PR_12_P = O­{(CH_2_CH_2_O)_3_C­(O)­C_6_H_4_PPh_2_}_2_).^24b^ The Cl derivatives show moderate cytotoxic activity against A549 and HeLa cancer cell lines, which was suggested to be related to the inhibition of tubulin polymerization. The C_6_F_5_ complexes and the compound [Pt­(Me_2_N-pbt)­(*o*-dpb)] generated ^1^O_2_, upon blue light irradiation, with excellent yields, and the PDT properties of the latter were demonstrated, showing IC_50_ values of 0.83 and 1.50 μM in A549 and HeLa cancer cell lines, respectively, upon 1 min of irradiation (396 nm), while remaining inactive in the dark. Furthermore, Cl complexes show cytoplasmic staining, which is more visible in the perinuclear area, whereas the ligand Me_2_N-pbtH and the C_6_F_5_ complexes specifically accumulate in the Golgi apparatus.

In the present work, we investigated the optical properties, supported by theoretical calculations, and the bioactivity against selected cancer cell lines of a series of mononuclear Me_2_N-pbt cyclometalated Pt­(II) complexes, [Pt­(Me_2_N-pbt)­(R-pic-κ*N∧O*)] (R = 3-H **1**, 3-NH_2_ **2**, 3-OH **3**, 4-COOH **4**). This series of cyclometalates was designed to consider the influence of changes in the nature of the substituent of the pic auxiliary ligand. Furthermore, the sensitization of ^3^O_2_ and formation of ^1^O_2_ have been studied in detail for **1**–**3**, and the potential photodynamic properties of these complexes have been evaluated.

## Results and Discussion

### Synthesis and Characterization

Complexes [Pt­(Me_2_N-pbt)­(R_1_-pic-κ*N∧O*)] (R_1_ = 3-H **1**, 3-NH_2_ **2**, 3-OH **3**) were isolated by conventional procedures as orange solids with good to moderate yields by the reaction of the solvate [Pt­(Me_2_N-pbt)­Cl­(DMSO)][Bibr cit24a] with the corresponding picolinic acid (1:1), in the presence of excess Na_2_CO_3_, in acetone at room temperature. Complex [Pt­(Me_2_N-pbt)­(4-COOH-pic-κ*N∧O*)] (**4**) was obtained as a black solid by the reaction of the same precursor with the 2,4-pyridinedicarboxylic acid ligand (1:1) in toluene at reflux for 8 h (Scheme 1). All complexes were fully characterized by elemental analyses, IR spectroscopy, electrospray ionization (ESI) mass spectrometry, 1D-NMR (^1^H and ^13^C­{^1^H}). and 2D–NMR experiments [^1^H–^1^H (COSY) and ^1^H–^13^C­{^1^H} (HSQC, HMBC)] (see Experimental Section and Figures S1–S4). All complexes exhibit one ν­(C=O) stretching band in the range of 1580–1661 cm^–1^ and one ν­(C–O) at 1326–1363 cm^–1^. Complex **2** shows the characteristic ν­(NH) band at 3383 cm^–1^, and **3** and **4** exhibit the ν­(OH) band at 3084 and 3455 cm^–1^, respectively. In **3**, the OH substituent of the picolinate can establish an interaction with the carboxylate, whereas in **4**, it is the OH of a carboxylic acid. Their ESI­(+)–MS spectra show [M + H]^+^ as a parent peak (*m*/*z*: 571.08 **1**, 586.09 **2**, 587.07 **3**, and 615.07 **4**), and the ESI–MS spectrum with negative ion mode for **4** exhibits [M – H]^−^ (*m/*z 613.00) as a parent peak. The ^1^H and ^13^C­{^1^H} NMR spectra show the expected set of signals corresponding to the Me_2_N-pbt cyclometalated and picolinate ligand in a 1:1 ratio. The ^1^H NMR spectra show, as the most deshielded signals, the corresponding H^7^ of the Me_2_N-pbt ligand (δ 9.08 **1**, 9.11 **2**, 8.86 **3** and 8.84 **4**), appearing as a doublet, and the picolinate H^6′^ proton (δ 9.29 **1**, 8.58 **2**, 8.74 **3** and 9.46 **4**) with the expected platinum satellites (^3^ *J*_*Pt–H*_ 38–46.5). H^11^, which appears as a doublet with Pt coupling (^3^ *J*_Pt–H_ 38.2–41.1 Hz), suffers a strong upfield shift (δ 6.49–6.61) compared to the precursor (δ 7.84). For **2**, the NH_2_ protons were found at δ 6.43, whereas for **3**, the hydroxyl of the OH-pic shows a narrow singlet at δ 13.21. The ^13^C­{^1^H} NMR spectra of **2** and **3** display, as the most deshielded signals, the corresponding C^2^ (δ 180.9 **2** and 180.6 **3**) and carboxylic carbon of the picolinate ligand (δ 176.6 **2** and 177.8 **3**). The methyl resonances of the NMe_2_ group of the cyclometalated ligand appear in the ^1^H and ^13^C­{^1^H} NMR spectra at the expected positions (δ_H_ 3.11 **1**, 3.10 **2**, 3.06 **3**, and 3.13 **4**; δ_C_ 40.3 **2** and 40.2 **3**).

**Scheme 1:**
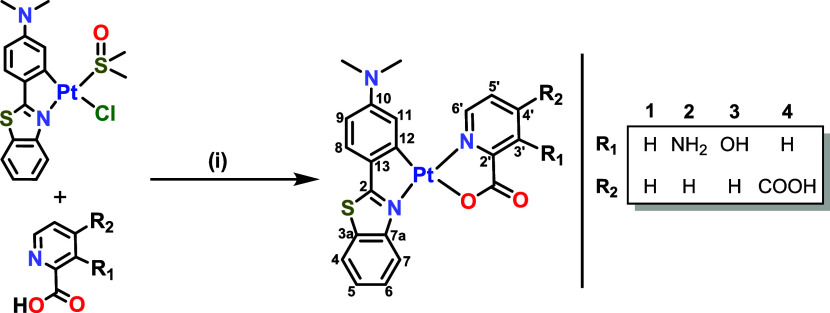
Labeling, Reagents, and Conditions: (i) Na_2_CO_3_ (Excess), Acetone 298 K, 5 h (for **1**, **2**, and **3**), or Toluene Reflux 8 h (for **4**)

### Crystal Structure Analysis

Orange and black single crystals of **1** and **3·CHCl**_**3**_ were obtained by slow diffusion of *n-*hexane into a solution of the corresponding complex in CHCl_3_ at room temperature and low temperature, respectively. **3·CHCl**_**3**_ contains two very similar molecules (**A** and **B**) and two molecules of CHCl_3_ in the asymmetric unit. Their molecular structures are depicted in Figures 1 and S5, S6, and the structural details are provided in Tables 1 and S1, S2. Red crystals of **4·DMSO** were grown from DMSO at low temperatures, but they were not good enough for an accurate resolution. However, connectivity and general packing could be established (Figure S7), revealing a slipped stack with short interplanar separation and long Pt··Pt distances. The X-ray diffraction analysis confirms in all cases the *trans*-N,N configuration, in which the N-coordinated atom of the picolinate ligand is in the *trans* position with respect to the N atom of the cyclometalated ligand (Me_2_N-pbt). In **1** and **3**, the Pt–C, Pt–N, and Pt–O bond lengths are similar to those found in other related complexes,^24,25^ indicating the negligible influence of the OH substituent on the bonding to Pt­(II). The bite angles [C_C∧N_–Pt–N_C∧N_ 80.61(9)° **1** and 80.92(9), 80.91(10)° **3·CHCl**_**3**_; N_C∧N_–Pt–O 79.69(8)° **1** and 79.46(8), 79.43(8)° **3·CHCl**_**3**_] are similar and in the expected range for five-membered metallacycles. The Me_2_N-pbt ligand displays a more planar disposition in complex **3** than in **1**. Thus, the angles between the phenyl ring and the benzothiazole group are 13.25° in **1** and 2.89 and 5.76° in **3**, and between the Me_2_N substituent and the Ph ring of 10.01° in **1** and 1.84 and 3.12° in **3**.

**Figure 1:**
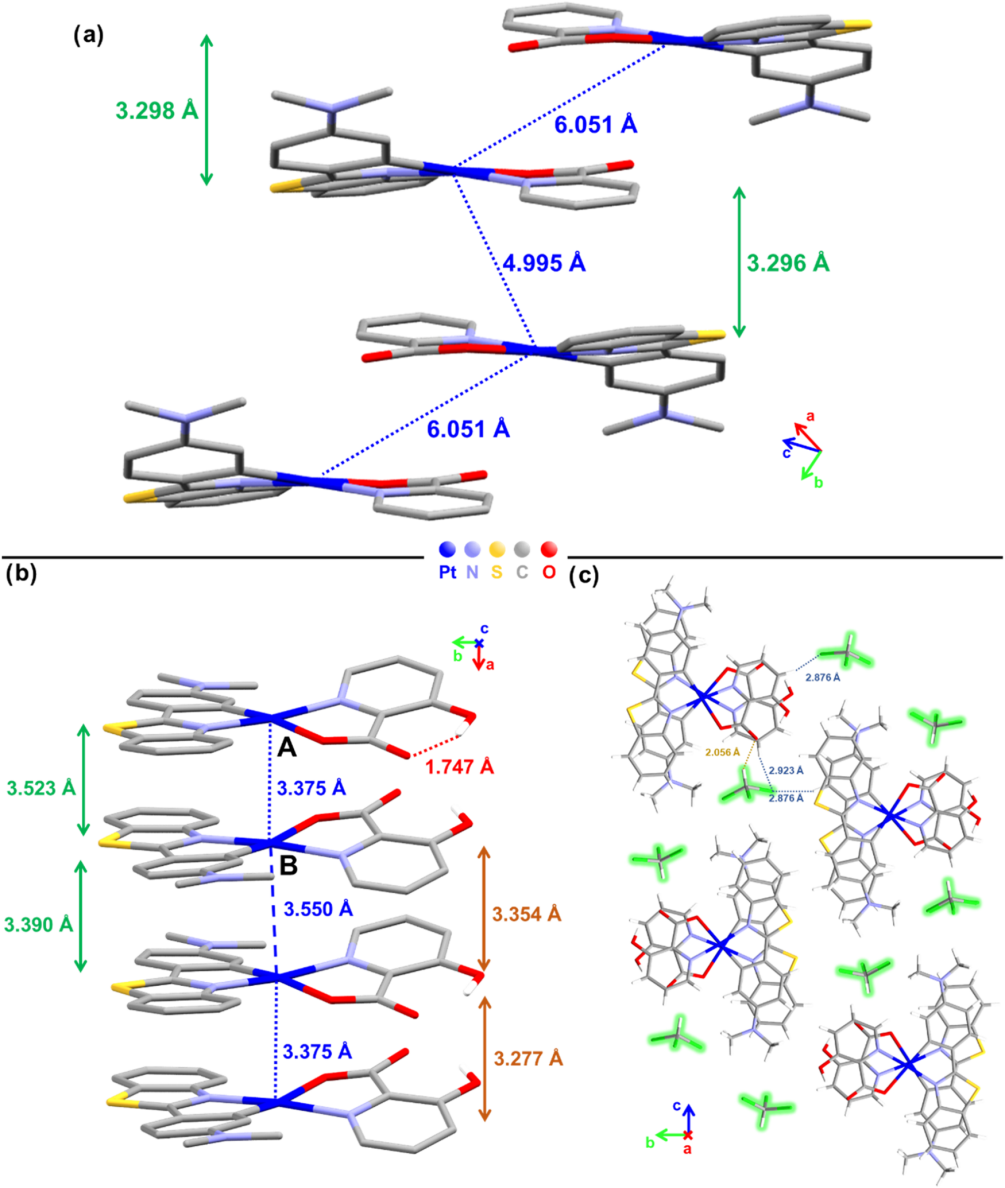
Packing structure of (a) **1** along the *b*-axis, (b) **3·CHCl**_**3**_ along the *a*-axis showing the π···π interplanar and Pt···Pt distances. (c) Top view from the *a*-axis of four stackings of **3·CHCl**_**3**_, showing CHCl_3_ solvent localization and secondary contacts.

**Table 1:** Color and Selected Distances (Å) of **1** and **3·CHCl**_**3**_

	**1**	**3·CHCl**_**3**_
color	orange	black
Pt–C_C∧N_ (Å)	1.999(2)	1.960(2) | 2.000(3)
Pt–N_C∧N_ (Å)	2.0185(19)	2.024(2) | 2.019(2)
Pt–N_N∧O_ (Å)	2.021(2)	2.031(2) | 2.027(2)
Pt–O_N∧O_ (Å)	2.1036(18)	2.1396(19) | 2.1363(19)
Pt···Pt (Å)	4.995 | 6.051	3.375 | 3.550
*d*_interplanar_ (Å)	3.296 | 3.298	3.390 | 3.523
		3.277 | 3.354

Complex **1** shows a staggered columnar packing along the *b-*axis, in a head-to-tail arrangement, supported by π···π stacking interactions with interplanar distances of ∼3.3 Å. The alternating Pt···Pt distances (4.995 and 6.051 Å) are longer than the sum of the van der Waals radius of Pt (3.50 Å),^26^ excluding Pt···Pt interactions, with a zigzag Pt–Pt–Pt angle of 86.42°. Between the columns, there are secondary weak intermolecular contacts with S···Ph_centroid_ distances of 3.484 Å (Figure S5).

In **3·CHCl**_**3**_, the two nearly identical molecules in the asymmetric unit form head-to-head dimers (AB), with a slightly staggered and *anti*-conformation (O–Pt–Pt-O torsion angles of 74.3 and 73.2°), which stack along the *a-*axis giving rise to extended columns with very short interplanar π···π [3.523 (dimer)/3.390 Å (interdimer)] and Pt···Pt [3.375 (dimer)/3.550 Å (interdimer)] distances,^26^ in accordance with the black color of the crystals. The Pt atoms in the columns are rather aligned with a Pt–Pt–Pt angle of 171.80°. The CHCl_3_ solvent molecules create channels along the *a-*axis, which are parallel to the Pt–[Pt]_n_–Pt arrangement, supported by C–H···O_pic_ (2.056 Å) and C–Cl···H–C_Ph/pic_ (2.753/2.923 Å) contacts (Figures 1c and S6). The total solvent volume occupies 24.9% of the total volume of the unit cell (1153.51 Å^3^ occupied by CHCl_3_ molecules in the unit cell). Additionally, an intramolecular hydrogen bond interaction between the carboxylate and the hydroxyl substituent of the picolinate ligand (O/H···O = 1.747 Å; O–H–O 152.45°) is also observed.

### Optical Properties

#### Absorption Properties and TD-DFT Calculations

The UV–vis absorption spectra of all complexes were recorded in solution (CH_2_Cl_2_ **1–3**; DMSO **4**; 5 × 10^–5^ M) and in the solid state at 298 K (Figures 2 and S8–S10 and Table S3). All assignments were done based on TD-DFT calculations in solution (PCM model) (see details in the Supporting Information, Tables S4–S7 and Figures S11–S12). The calculated bond distances and angles for complexes **1** and **3** are similar to those found by X-ray crystallography (Tables S4, S5), indicating the accuracy of the calculations at this level of theory (B3LYP). In solution, they show absorptions in the high-energy region (λ < 330 nm; ε = 10^4^ L·mol^–1^·cm^–1^) attributed to intraligand transitions (^1^IL, π–π*) located on the cyclometalated Me_2_N-pbt ligand with some charge transfer from the Pt­(Me_2_N-pbt) core to the picolinate ligand ^1^(M + L)­L′CT (L′ = pic). They exhibit an additional band at around 370 nm and a characteristic low-energy feature (λ > 425 nm; ε ∼ 1.5 × 10^4^ L·mol^–1^·cm^–1^), similar in shape for all of the complexes and only slightly blue-shifted for the carboxylic complex **4** (460 **1**, 461 **2**, 464 **3**, and 453 nm **4**), suggesting that the functionalization of the picolinate ligand has a minor influence on the transition (Figure 2). The UV–vis spectra of complex **1** were examined in solvents with different polarities, with minimal differences found between CH_2_Cl_2_ and THF; whereas in MeOH, the lowest-energy band became broader (Figure S8), probably due to partial protonation of the NMe_2_ group.

**Figure 2:**
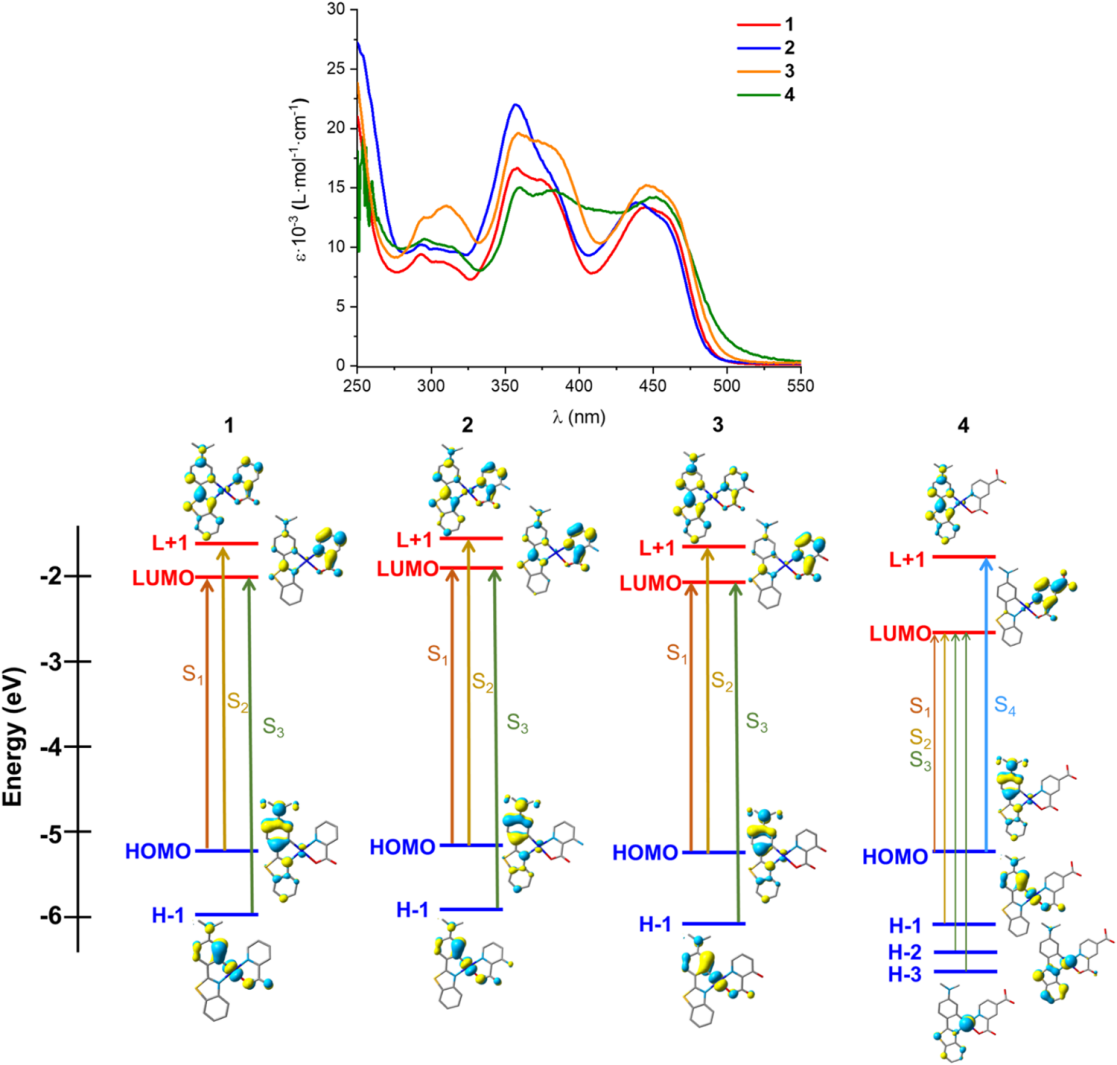
UV–vis absorption spectra in solution (CH_2_Cl_2_ **1**–**3**) and DMSO (**4**) (5 × 10^–5^ M), and schematic representation of the low-energy calculated excitations.

Theoretical calculations indicate that HOMOs are located on Me_2_N-pbt (∼95%), particularly on the dimethylaminophenyl group, with minor Pt contribution (∼4%), which considerably increases in H-1 (Pt 44% **1**, 42% **2**, 43% **3**, **4**). LUMOs are mainly located on the picolinate ligand (67% **1**; 58% **2**; 73% **3**; 92% **4**) with the contribution of the cyclometalated ligand being greater in complexes **1–3** (28% **1**; 37% **2**; 22% **3**; 4% **4**), whereas the contribution of the cyclometalated ligand increases in L+1 (69% **1**, 60% **2**, 75% **3**, 91% **4**). For complexes **1–3**, the calculated low-energy S_1_ excitation (458 **1**, 448 **2**, and 468 nm **3**) is dominated by the HOMO–LUMO transition (96–99%). Therefore, the low-energy absorption band is mainly attributed to Me_2_N-pbt to picolinate charge transfer (^1^LL′CT) with an intraligand charge-transfer contribution (^1^ILCT; Me_2_N-Ph→bt). The absorption at ∼370 nm can be ascribed to the calculated S_2_ and S_3_ excitations, having a ^1^(M + L)­L′CT mixed characteristics with some ^1^IL nature (Figure 2). In **4**, the S_1_–S_3_ transitions, with low oscillator strength, are derived from HOMO/H-1/H-2/H-3 → LUMO transitions and have mainly ^1^LL′CT (S_1_) and a mixture of ^1^(M + L)­L′CT (S_2_, S_3_) characteristics. The next most intense transition (S_4_, 404 nm, HOMO → LUMO+1 84%) has mainly intraligand ^1^ILCT contributions.

Based on each UV–vis absorption spectrum, the optical gap (*E*_optical gap_) can be estimated by using eq 1:^27^

1 $$E_{\mathrm{optical gap}}=\mathrm{hc}/\lambda_{\mathrm{onset}}∼1240/\lambda_{\mathrm{onset}\left(\mathrm{nm}\right)}$$

where λ_onset_ denotes the absorption edge wavelength expressed in nm, obtained from the offset wavelength derived from the lowest-energy absorption band, as shown in Figure S9 for **1**–**4**. According to the experimental results, band gap energies (*E*_gap_) were calculated for all complexes (2.54 **1**, 2.55 **2**, 2.51 **3**, 2.47 eV **4**, Table 2). They follow the same tendency as the energy difference HOMO–LUMO calculated by TD-DFT (3.21 **1**, 3.26 **2**, 3.15 **3**, 2.60 eV **4**).

**Table 2:** Absorption and Emission Data in Solution for Complexes **1–3** in CH_2_Cl_2_ and **4** in DMSO

		**emission 298 K**					**emission 77 K**		
		**5 × 10^–^**^**5**^ **M**				**5 × 10^–^**^**4**^ **M**	**5 × 10^–^**^**5**^ **M**		
	absorption λ_**max**_ **/nm** ^a^	**λ**_**em**_ **/nm (λ**_**exc**_ **/nm)**	**ϕ** ^b^	**ϕ** ^c^	**τ** ^d^ **/μs [λ**_**em**_ **/nm]**	**λ**_**em**_ **/nm (λ**_**exc**_ **/ nm)**	**λ**_**em**_ **/nm (λ**_**exc**_ **/ nm)** ^e^	*E*_**optic gap**_ **/eV** ^f^	*E*_**HOMO–LUMO–DFT**_ **/eV**
**1**	247, 293, 315_sh_, 358, 376, 445, 460	409, 575, 613, 679_sh_ (365)	0.41	<0.01	0.0016 [409]^g^	576, 619, 680_sh_ (480)	568, 622, 670_sh_ (460)	2.54	3.21
					14.1 [575]^h^				
**2**	250, 293, 313_sh_, 357, 381_sh_, 439, 461	409, 574, 617, 676_sh_ (365)	0.33	<0.01	0.0016 [409]^g^	576, 618, 680_sh_ (478)	564, 616, 670_sh_ (460)	2.55	3.26
					12.7 [574]^h^				
**3**	243, 295, 310, 322_sh_, 359, 385_sh_, 446, 464	409, 577, 619, 678_sh_ (365)	0.12	<0.01	0.0016 [409]^g^	579, 619, 684_sh_ (485)	570, 623, 680_sh_ (460)	2.51	3.15
					12.4 [577]^h^				
**4**	295, 315, 360, 384, 453	428, 492, 590, 625 (365)	0.04^i^	<0.01^i^	0.0015 [492]^g^;	430, 520, 590, 628 (365)	568, 610, 667_sh_ (456)	2.46	2.60
		492, 590, 625 (450)	0.08^j^	0.02^j^	3.8 (77%), 12.0 (23%) [588]^h^	517, 590, 628 (443)			

a **1–3** in CH_2_Cl_2_ and **4** in DMSO (5 × 10^–5^ M). The ε values are given in Table S3 in the SI.

b Deoxygenated conditions.

c Oxygenated conditions.

d λ_exc_ 390 nm (LED). Decays are shown in Figure S22.

e **1**–**3** in CH_2_Cl_2_/MeOH 1:1.

f Values calculated according to eq 1: *E*_optical gap_ = *hc*/λ_onset_ ∼ 1240/λ_onset (nm)_.

g Lifetimes measured in the fluorescence band.

h Lifetimes measured in the phosphorescence band.

i λ_exc_ 365–390 nm.

j λ_exc_ 400–450 nm.

The solid-state absorption (diffuse reflectance) spectra of the *as-obtained* powders of **1**–**4** were also examined (Figure S10). Complexes **1**–**3** show similar lowest-energy bands at 472–498 nm, in agreement with their orange color, with low intense features in the tails (562–575 nm), probably of spin-forbidden nature. However, **4** displays an intense absorption band in the NIR region (725 nm with a tail to 800 nm), clearly red-shifted in relation to **1**–**3** and in agreement with its black color. This black color is likely associated with strong Pt···Pt bonding interactions in the solid.

#### Emission Properties and DFT Calculations

As mentioned in the Introduction, Pt­(II) derivatives show advantageous photophysical properties. Interactions between square planar Pt­(II) complexes, particularly those with rigid and planar skeletons, lead to assemblies that modulate their excited state properties as a function of their π···π and/or Pt···^.^Pt interactions, giving lower-energy emissions than the isolated molecules.^28^ Notwithstanding, aggregation and self-assembly through intermolecular interactions can give rise to aggregation-induced emission,^29^ with improved quantum yields or a quenched emission in the aggregate state in an aggregation-induced quenching (AIQ) effect.^30^

The emission spectra of complexes **1**–**4** were registered in solution (**1**–**3** CH_2_Cl_2_, **4** DMSO) at 5 × 10^–4^ and 5 × 10^–5^ M, in the solid state at 298 and 77 K, and in the polystyrene (PS) films at 1% *wt* (**1**–**3**) (Figures S13–S22 and Tables 2 and 3). In PS at 298 K, complexes **1**–**4** exhibit vibronically structured profiles with a minor change in their λ_em_ maxima (568–580 nm) (Figure S13a for **3**, Table 3) and long radiation decay times (τ = 17–32.4 μs), which are ascribed to an intraligand ^3^ILCT excited state localized on the benzothiazole group (Me_2_N-ph→bt) for **1**–**3** and ^3^LL′CT/^3^ILCT for **4**, based on DFT calculations (see below). A concentration-dependent study was made for complexes **1** and **3**, studying the concentration series of PS films at 50, 20, 10, 5, and 1% (Table 3). Upon increasing the concentration, a clear aggregation quenching effect appeared with a slight change in the emission profiles (from yellow to orange in **3**, Figure S13b) due to the increasing contribution of excimer-like low-energy emission. This is accompanied by a drastic reduction in quantum yield in deoxygenated conditions from 0.21 (1%) to 0.05 (20%) for **1** and from 0.25 (1%) to 0.02 (50%) for **3** (Table 3). As is well known, due to the long emission lifetimes, severe triplet–triplet annihilation (TTA) will occur at high concentrations in films where the formation of aggregate states through π···π interactions usually quench the phosphorescence of organometallic complexes.^31^ Indeed, a similar quenching effect was observed in the solid state (see below). Furthermore, it is worth noting that the emission intensity of the films also decreases notably in aerated conditions (see Table 3) for the same concentration.

**Table 3:** Photophysical Data in Solid State and PS Films for Complexes **1–4**

**compound**	**media**	***T*/K**	**λ**_**em**_ **/nm (λ**_**ex**_ **/nm)**	**ϕ** ^**deox**^ **(ϕ** ^**ox**^ **)** ^a^	**τ/μs [λ**_**em**_ **/nm]** ^b^
**1**	solid	298	595, 638, 720_sh_ (530)	0.01	2.5 (49%), 17.2 (51%) [595]
		77	615, 665 (515)		35.3 [615]
	PS 1%^c^	298	572, 612, 673_sh_ (443)	0.21 (0.13)	17.4 [572]
**2**	solid	298	655 (525)	<0.01	1.9 (40%), 15.2 (60%) [655]
		77	660, 724 (520)		17.9 [660]
	PS 1%	298	568, 611, 672_sh_ (444)	0.18 (0.09)	17.5 [568]
**3**	solid	298	576, 625, 675_sh_ (500)	<0.01	2.2 (48%), 17.4 (52%) [576]
		77	600_sh_, 625, 650 (500)		28.7 (80%), 65.1 (20%) [600]; 31.3 (73%), 65.7 (27%) [625]
	PS 1%^d^	298	580, 611, 679_sh_ (450)	0.25 (0.17)	17.0 [580]
**4**	solid	298	630, 860 (420–540)	<0.01	2.9 (24%), 14.7 (76%) [630]; 2.4 (38%), 12.2 (62%) [880]
	PS 1%	298	579, 615, 670_sh_ (450)	0.18 (0.12)	32.4 [580]

a λ_ex_ 450 nm.

b Decays are shown in Figure S22.

c Increasing complex/PS ratio; peak maxima do not change, but ϕ^deox^ decreases: 0.10 (5%), 0.07 (10%), and 0.05 (20%).

d Increasing complex/PS ratio; peak maxima do not change, but ϕ^deox^ decreases: 0.12 (5%), 0.05 (10%), 0.05 (20%), and 0.02 (50%).

In the CH_2_Cl_2_ solution, the effect of oxygen is remarkable. Thus, in deaerated solutions (5 × 10^–4^ M) at 298 K, **1**–**3** display, upon excitation in the low-energy band, a similar structured phosphorescence emission (λ_em_ 576–579 nm) (Figures 3 and S15, Table 2) with quantum yields (ϕ) of 41% **1**, 33% **2**, and 12% **3**, which decreased considerably under aerated conditions (ϕ < 1%) (Figure S14), suggesting the strong occurrence of energy transfer to ^3^O_2_ ^10,24a,32^ among other quenching processes. Calculations confirm that the HSOMO and LSOMO and the spin density surface for the optimized T_1_ of **1**–**3** are located mainly on the Me_2_N-pbt cyclometalated ligand (the Pt character is only around 0.07 in the spin density), confirming a metal-perturbed ^3^ILCT character for the emission (Table S8 and Figure 4). Curiously, the complexes emit a visible blue light in solution with a UV–vis hand-lamp; therefore, the emission upon excitation at 365 nm was also examined. Upon excitation at 365 nm (5 × 10^–5^ M solution), corresponding to S_2_ and S_3_ excitations, a dual emission is observed formed by moderate short lifetime fluorescence in the blue region (409 nm; τ 1.6 ns, Figure 3b for **2**, and S15 for **1** and **3**) and the phosphorescence band in the yellow region (∼575 nm), which reduces notably under air-equilibrated solutions. The fluorescence observed upon excitation at 365 nm has ^1^(M + L)­L′CT character. Excitation in this region is mainly related to strong S_2_ and S_3_ excitations, which involve transitions from low-lying HOMO and H-1 orbitals having remarkable NMe_2_-pbt (HOMO ∼ 95%) and platinum (H-1, 42–44%) weight to LUMO and L+1 located on the picolinate and pbt (see Tables S6, S7 for details). Therefore, the direct deactivation from this state [^1^(M + L)­L′CT]* seems to compete with the internal conversion (IC) to the low-lying intraligand excited state (S_1_*, ^1^ILCT/^1^LL′CT)*, which finally leads to T_1_ of ^3^ILCT character. We also note that this blue fluorescence at 409 nm overlaps with the low-lying absorption band of ^1^ILCT/^1^LL′CT (Me_2_N→pbt/Me_2_N-pbt→pic), and, under these conditions, it is likely that the emitted fluorescence sensitizes molecules in the S_0_ state to the low-lying metal-perturbed ^3^ILCT. According to the F:P relation, this process seems to be similar for **2** and **3** and to a lesser extent for **1**. An additional solvent-dependent emission study at 298 K in a diluted solution (5 × 10^–5^ M) was carried out for **1** in other solvents with different polarities (THF and MeOH), and the results are shown in Figure S16. Upon excitation to 450 nm, **1** shows identical phosphorescence with negligible influence on the maxima in THF and CH_2_Cl_2_. However, in MeOH, the emission is dominated by the high-energy band with the phosphorescence maxima slightly blue-shifted and decreased, probably due to the quenching effect favored by the protonation of the NMe_2_ group.

**Figure 3:**
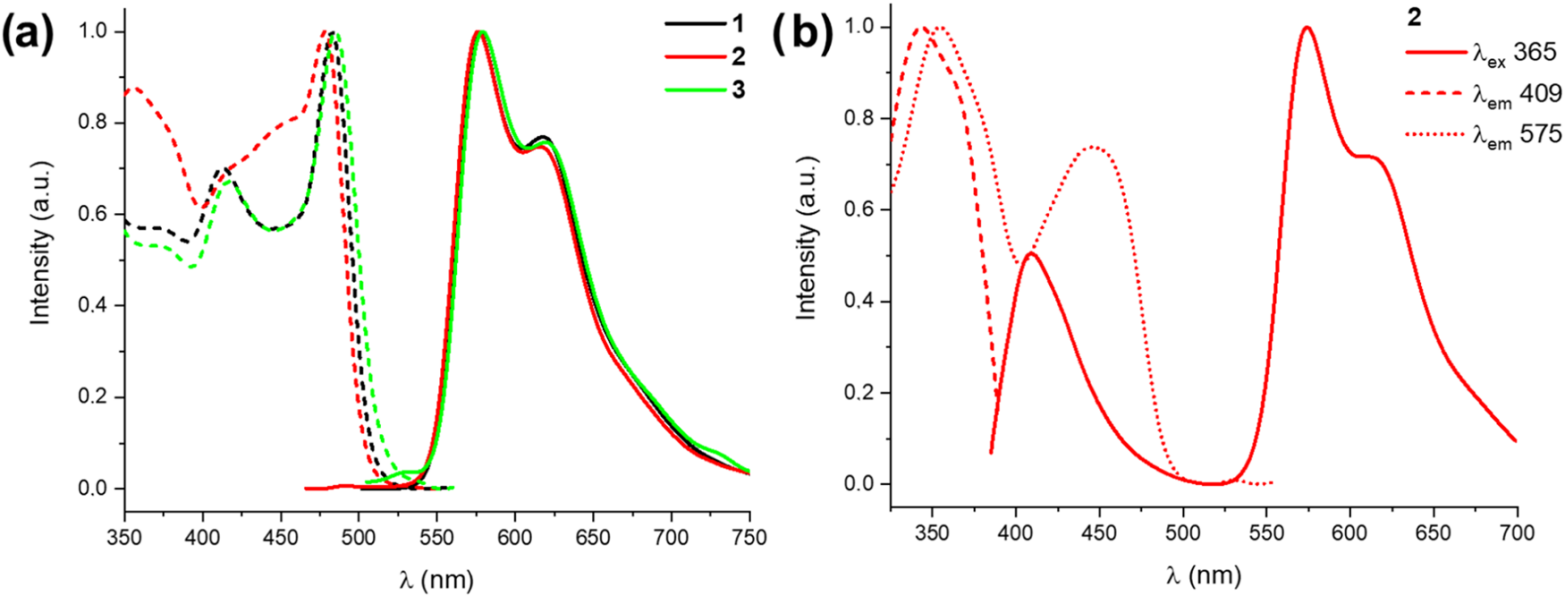
Normalized excitation and emission spectra of (a) complexes **1**–**3** in deaerated CH_2_Cl_2_ solution (5 × 10^–4^ M, λ_ex_ ∼ 455 nm), and (b) complex **2** (CH_2_Cl_2_, 5 × 10^–5^ M, λ_ex_ ∼ 365 nm).

**Figure 4:**
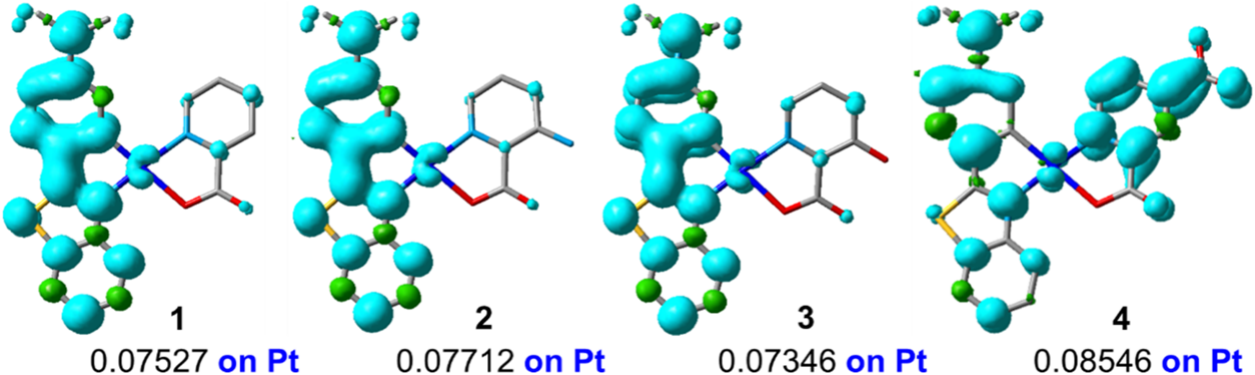
Spin density distribution of the optimized T_1_ triplet state in CH_2_Cl_2_ (**1**–**3**) and DMSO (**4**).

In a glassy solution (CH_2_Cl_2_/MeOH 1:1, 5 × 10^–5^ M, 77 K), they show a more structured ^3^ILCT emission, with the highest energy peak slightly blue-shifted in relation to 298 K, regardless of the excitation wavelength used (Figure S17). This rigidochromic effect is not unusual and has been related to CT transitions.^33^

In the solid state at room temperature, the emission of complexes **1**–**3** is notably quenched, likely due to easy triplet–triplet annihilation facilitated by closer intermolecular interactions (Table 3).^34^ Thus, **1** and **3** exhibit a very weak (**ϕ <** 1%) vibronic emission at 298 K, which is slightly red-shifted to that observed in solution (Figure S18a,b), whereas **2** displays a broader excimer-type emission located at 655 nm, likely driven by π···π and/or Pt···Pt interactions in the ground state (Figure S18c). As expected, the intensity is enhanced at 77 K. Their lifetimes at room temperature fit to two components with a τ_average_ of ∼10 μs, indicating the existence of close emissive excited states, which prolong notably at 77 K (τ = 35.3 **1**, 17.9 **2**, 36 μs **3**).

In the solid state at 298 K, complex **4**, bearing the COOH group, has a weak dual emission in the red (λ 630 nm) and NIR regions (860 nm), whose intensity depends on the excitation wavelength (Figure S19). The band at 630 nm is ascribed to a mixed ^3^LL′CT/^3^ILCT excited state on the monomer, whereas the NIR emission at 860 nm is likely due to a mixed metal–metal charge transfer to both ligands [^3^MM­(L+L′)­CT] caused by the strong interaction of monomers through short Pt··Pt interactions.

Complex **4** is only soluble in DMSO and, in deoxygenated DMSO solution, it exhibits, upon excitation of the LE band (λ_exc_ 440–475 nm), a strong long-lived LE band in the yellow-orange region (∼590 nm) red-shifted in relation to **1**–**3** (575 nm), pointing to a different emitting state. According to calculations [HSOMO: Me_2_N-pbt, 11%/4-COOH-pic, 85%/Pt, 4%; LSOMO: Me_2_N-pbt 93%/Pt 6%] (Table S8) and the T_1_ spin density distribution (Figure 4), the LE-structured phosphorescence band is ascribed to a mixed ^3^LL′CT/^3^ILCT excited state. A minor high-energy (HE) feature with a short lifetime (τ 1.5 ns) in the blue-green region (∼ 492 nm) is also observed (Figure 5). This HE band exhibits a characteristic mirror band shape with the longest wavelength absorption band and is thus assigned to the fluorescence of metal-perturbed ^1^ILCT/^1^LL′CT (Me_2_N→pbt/Me_2_N-pbt→pic) character. As expected, the fluorescence clearly increased in relation to the phosphorescence in oxygenated solutions due to the partial quenching of the LE phosphorescence band (Figure 5). The quantum yield, upon excitation at 450 nm in degassed solution, is 8%, and it is reduced to 2% in oxygenated solutions. The excitation spectra monitoring in both bands reproduces approximately the absorption spectrum in the LE region, indicating that both emissions come from the same complex. The observed emission is dependent on the excitation wavelength. Upon excitation at 365–390 nm, dual fluorescence is seen in the HE region with two maxima (λ 428, 492 nm), and the structured LE phosphorescence band at 590 nm is also sensitive to the air in solution (ϕ_deoxygenated_ 4% vs ϕ_oxygenated_ < 1%). As discussed, the peak at 492 nm which is ascribed to ^1^ILCT with ^1^LL′CT contribution. The new F band at 428 nm is related to an excitation peak in the 350–375 nm region, which can be correlated with S_6,7_ having a mixed ^1^LL′CT/^1^ML′CT nature. The excitation spectrum of the phosphorescence is identical to that observed for the LE band upon excitation in the LE region (440–475 nm; Figures 5 and S20). This wavelength dependence suggests the occurrence of hyperintersystem crossing (HISC) from S_6,7_ (^1^LL′CT/^1^ML′CT) to T_1_, competing with internal conversion (IC) to S_1_. This rare behavior has been previously observed in some related complexes in which the relaxed S_1_ states have strong ππ* character and negligible metal contribution.^10^ At 77 K, only a blue-shifted (568 nm) phosphorescence is observed regardless of the excitation wavelength used (Figure S21).

**Figure 5:**
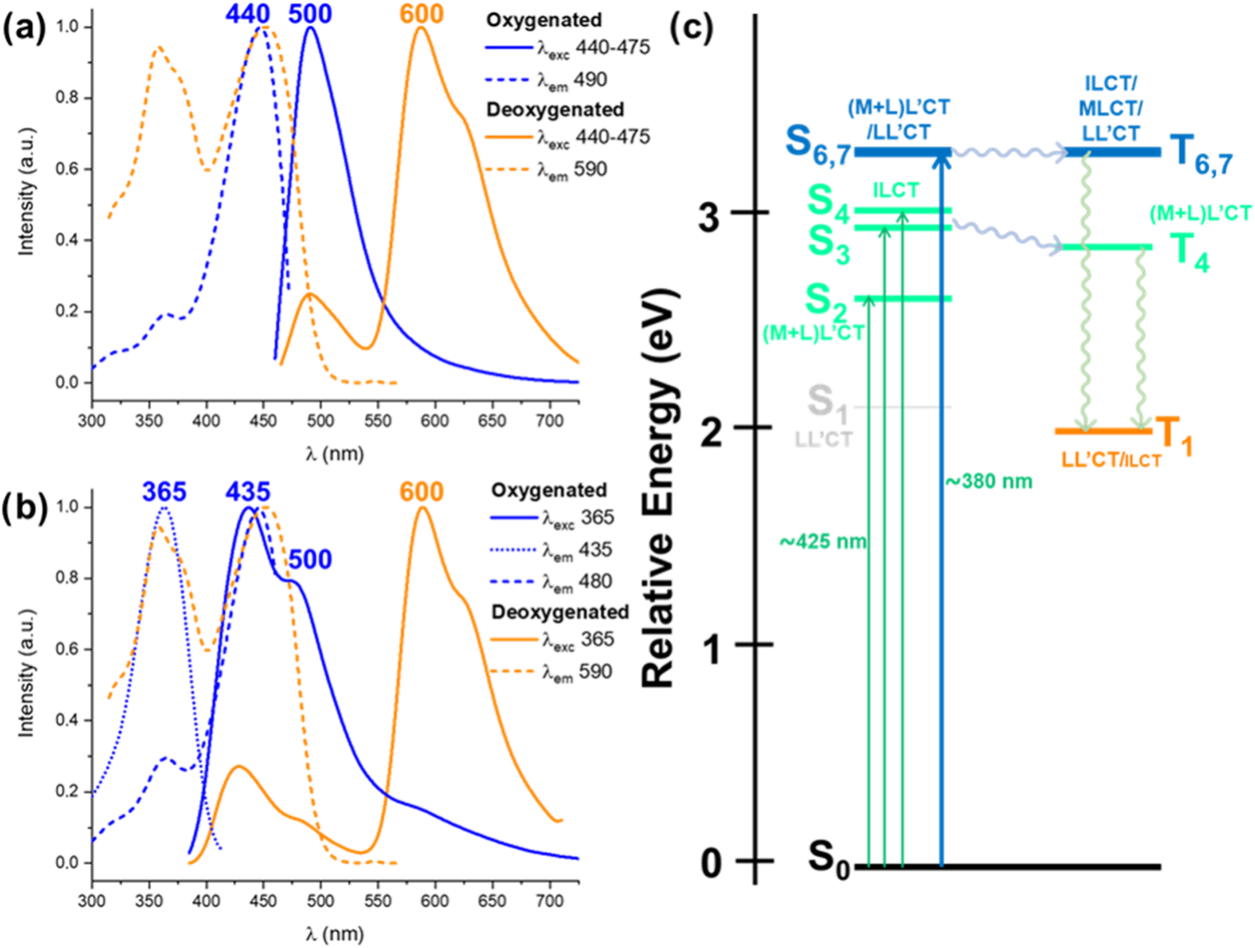
Excitation and emission spectra of complex **4** in oxygenated and deoxygenated DMSO solutions (5 × 10^–5^ M) at 298 K upon excitation at (a) 440–475 nm and (b) 365 nm. (c) Relative energy and character of the more intense vertical singlet and triplet excitations in the S_0_ geometry of **4**.

In aerated DMSO solutions, complexes **1**–**3** display a fluorescence band only at *ca* 510 nm, attributed to ^1^LL′CT/^1^ILCT, due to easy quenching of the phosphorescence by oxygen. However, upon prolonged photoexcitation at 365 nm, a continuous enhancement of the phosphorescence band is observed. Simply irradiating with a hand UV–vis lamp, the initial blue-green emission gradually shifts to the orange enhanced emission. The phosphorescence increases its maximum intensity in *ca* 15 **1**, 8 **2**, or 12 min **3** (Figures 6 and S23). The intense orange emission was reversibly switched off by simply shaking the solution, allowing its oxygenation, and can be repeated several times, although it does not take place in other solvents such as acetonitrile, THF, or CH_2_Cl_2_. This relatively rare behavior has been previously observed by us^10,24b^ and other groups,^35^ and is explained by the occurrence of a local sensitization caused by energy transfer from the low-energy triplet to ^3^O_2_-producing singlet ^1^O_2_, which is able to selectively react with the solvent creating a free oxygen microenvironment that *switches-on* a phosphorescence band due to ^3^ILCT at 580 nm. The strong sensitivity of the phosphorescence emission in these complexes encourages us to determine their efficiency as ^1^O_2_ sensitizers. The singlet oxygen generation of complexes **1**–**3** was examined in acetonitrile solution (5 × 10^–5^ M) using a near-infrared detector upon excitation at 365 nm, detecting the characteristic emission profile of ^1^O_2_ at λ_em_ ∼ 1274 nm (Figure 7). The measured quantum yield (ϕ_Δ_) of ^1^O_2_ was calculated at 298 K in aerated acetonitrile solution (5 × 10^–5^ M) in comparison with free phenalenone (PN), a universal reference compound that can be used in various solvents.^10,32^ The measured quantum yields (ϕ_Δ_) of ^1^O_2_ upon excitation at 365 nm were 13.6 **1**, 17.4 **2**, and 16.2% **3**, suggesting that these complexes can be used as photosensitizers.

**Figure 6:**
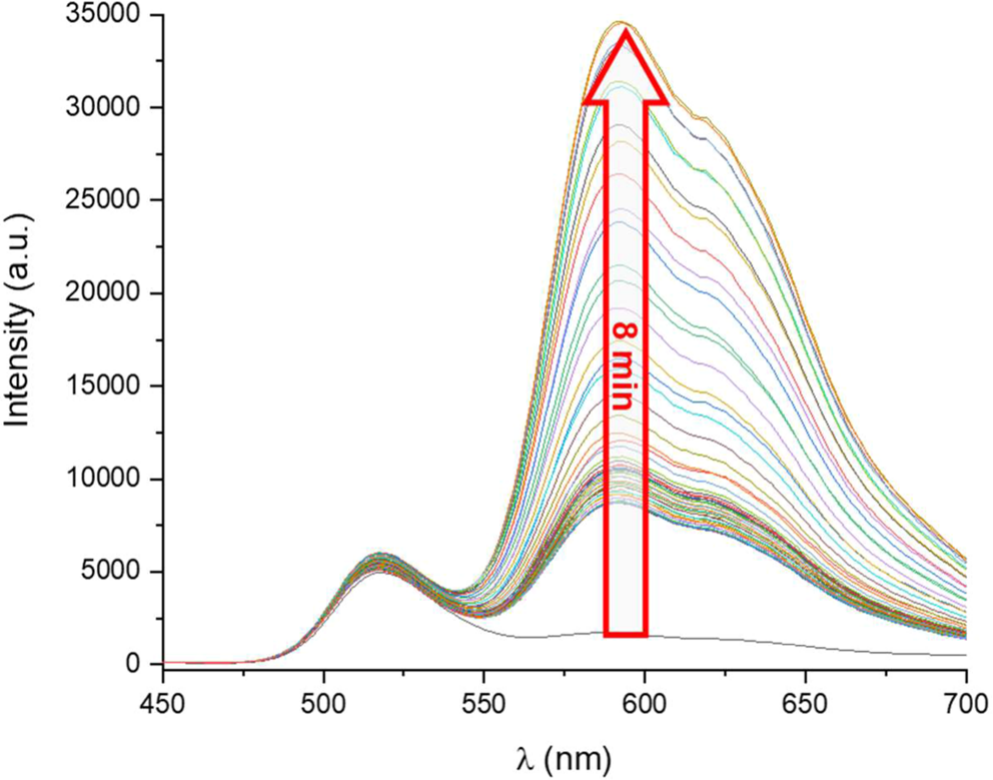
Enhancement of phosphorescence emission of **2** in an aerated DMSO solution upon excitation at 365 nm and emission spectra in DMSO 5 × 10^–4^ M solution in the presence of O_2_ at different irradiation times.

**Figure 7:**
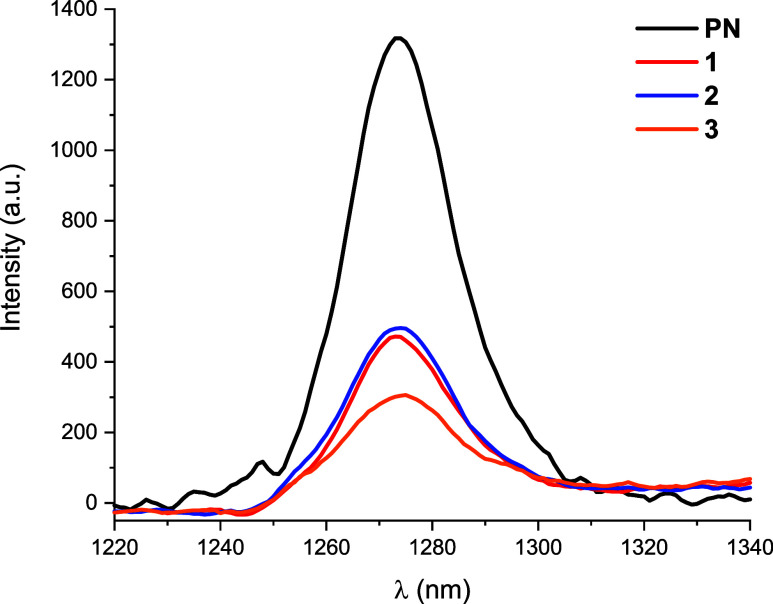
Emission band of singlet oxygen from fresh solutions of phenalenone (PN) and complexes **1**–**3** in CH_3_CN solution (5 × 10^–5^ M) (λ_ex_ 365 nm).

Additionally, the aggregation ability of these derivatives was studied by comparing the emission spectra in aerated DMSO–H_2_O mixtures, keeping the concentration constant, with different H_2_O fractions. Taking complex **3** in DMSO solution (2 × 10^–4^ M) as an example, as shown in Figure 8, only ^1^LL′CT/^1^ILCT fluorescence at 510 nm is seen upon excitation to 450 nm. Phosphorescence is quenched under these conditions. As the water content increases from 0 to 40%, the fluorescence band remains, and a very broad phosphorescence (P) band at ∼720 nm begins to appear. When the water fraction is above 40%, the fluorescence band is slightly red-shifted to 525 nm and then slowly decreases in intensity, and P at 720 nm in DMSO–H_2_O mixtures increases significantly to yield approximately 10-fold enhancement. The development of this broad emission, which is notably red-shifted with respect to the structured band observed in deaerated solutions at *ca* 600 nm, is attributed to mixed ^3^MMLCT/^3^ππ* due to the formation of small nanoaggregates (Figure S24) upon increasing the water content, in agreement with the aggregation observed in the X-ray structure of **3**·**CHCl**_**3**_. For example, when the water fraction reaches 50%, the fluorescence quantum yield is ca. 0.4%, and in the red phosphorescence band reaches ϕ = 0.3%, whereas with further amounts of water (*f*_w_ 90%), P reaches a quantum yield of 0.5% and the fluorescence practically disappears. We note that the contribution of these aggregates decreases notably when using a lower concentration of **3** in the initial solution (5 × 10^–5^ M, Figure S25a), with P enhanced from *f*_w_ 50%. This is confirmed by the red-shifted tails in the visible region of the absorption spectra of complex **3** in aqueous mixtures with a high water content (*f*_w_ > 40%) (Figure S25b).

**Figure 8:**
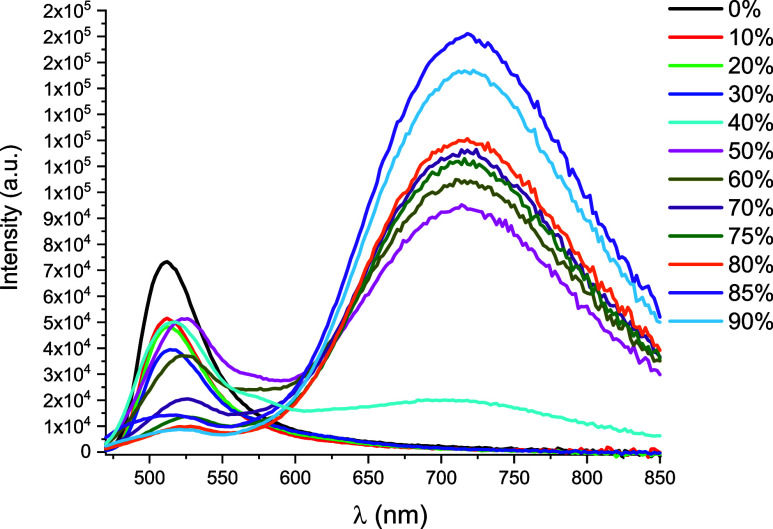
Emission spectra of **3** (2 × 10^–4^ M) in DMSO–H_2_O mixtures with different H_2_O fractions (0–90%) at 298 K (λ_exc_ = 450 nm).

### Biological Studies

#### Stability Tests

First, the stability of complexes **1**–**4** was evaluated by ^1^H NMR in DMSO-*d*_6_ solution and DMSO-*d*_6_/D_2_O (9/1) (Figures S26–S27). ^1^H NMR stability studies showed that all complexes are stable in DMSO or DMSO/D_2_O (9/1) after 72 h at room temperature. Also, the photostability of all derivatives was assessed by ^1^H NMR (Figures S28–S29), which showed good photostability upon 30 min irradiation at 460 nm in DMSO-*d*_6_ and DMSO-*d*_6_/D_2_O (9/1), conditions that exceed those used in the photoinduced activity assays (5 min). The stability of complexes **1**–**4** was then investigated in more biologically relevant media by UV–visible spectroscopy. First, the stability of the complexes was studied in phosphate-buffered saline (PBS) containing 5% DMSO (Figure S30). In this case, no particular spectral changes could be observed after 24 h at 37 °C in the dark, suggesting the stability of the complexes in these conditions. Only a decrease in the intensity of all bands was observed, which could be attributed to partial aggregation and/or precipitation. Likewise, after 4 h of incubation followed by 5 min of irradiation at 450 nm, the complexes did not undergo any spectral changes, indicating an absence of structural modification. The stability of complexes **1**–**4** was next studied in serum-free DMEM cell culture containing 5% DMSO (Figure S31). In this medium, the UV–vis spectrum of complex **4**, with the carboxylic acid substituent, showed clear modifications with the appearance of an intense band at 415 nm, suggesting a quantitative transformation within the first 4 h of incubation, which appeared insensitive to irradiation (Figure S31D). The UV–vis spectrum of complex **1** with no substituent on the pyridine ring showed a first spectral modification with a red shift of the maximum absorption to 390 nm occurring within the first 4 h of incubation, which was similar in dark and irradiation conditions. After 24 h, the UV–vis spectrum of **1** further evolved with the appearance of an intense band at 418 nm, suggesting, along with the shoulder at around 390 nm, an additional transformation of the transient species observed after 4 h of incubation at longer incubation times. This final species seemed to have spectral features similar to those observed for complex **4** (Figure S31A). Finally, no spectral changes in the first 4 h of incubation in the dark or irradiation for complexes **2** and **3** featuring an NH_2_ or an OH substituent on the pyridine ring, respectively, suggest their stability at short incubation times. After 24 h of incubation, the spectral features of both complexes changed, leading to a broader band at higher wavelengths (between 390 and 415 nm), suggesting the transformation of both complexes to similar species, i.e., a mixture of the transient and final species observed for complexes **1** and **4** (Figure S31B/C). The similarity of the spectral features of the transformed species in all cases seemed to point to the formation of a common species upon incubation in DMEM, which we tentatively attribute to the decoordination of the picolinate ligands with a substituent-dependent kinetics; the COOH group favored the release of the picolinate ligand, while both NH_2_ and OH substituents seemed to slow down this process.

#### Binding to BSA

The possible interaction of these Pt­(II) complexes with the model protein bovine serum albumin (BSA) was studied by fluorescence and UV–vis spectroscopy.^36^ For example, complex **3**, in an aerated DMSO solution (10 μM), only shows an emission band at 510 nm by excitation at 450 nm. The effect of increasing the amount of BSA (from 0 to 50 μM) on solutions of complex **3** in mixtures of H_2_O/DMSO (95/5 v/v) while keeping the concentration of **3** (10 μM) constant was evaluated. Upon addition of BSA (**3**/BSA 1:0.25), a slight blue shift of the emission band from 510 to 495 nm is observed, and a clear *switch-on* of the monomer phosphorescence (^3^ILCT), as a structured band slightly blue-shifted (*ca*. 574 nm) in relation to the complex in DMSO is observed. The intensity of this latter band reaches a maximum when the **3**/BSA ratio was *ca* 1:1 (Figure 9). The UV–vis spectra of the mixtures of BSA and **3** show, as expected, an increase of the absorption peak of BSA at 278 nm and a change in the low-energy absorption bands of **3**, as the concentration of BSA increased (from 2.5 to 50 μM, Figure S32). Thus, as the concentration of BSA increases, the low-energy band becomes slightly blue-shifted, and a new band appears at ca. 405 nm. These specific changes in the photophysical properties indicate that complex **3** interacts strongly with BSA. In particular, the decrease in fluorescence emission and the *switch-on* of the monomer phosphorescence can be attributed to the binding of **3** to the hydrophobic pockets of the protein, which increases the rigidity of its environment and decreases the nonradiative constant.

**Figure 9:**
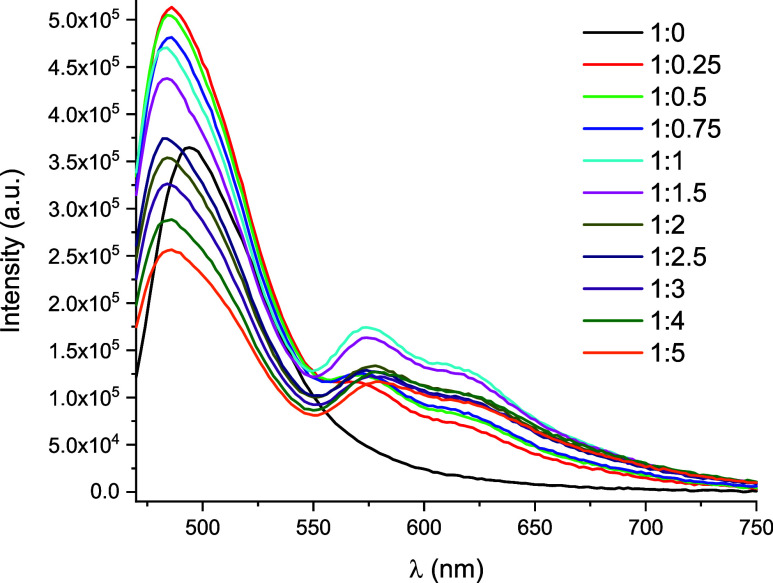
Emission spectra of solutions of complex **3** and BSA in different proportions (from 1:0 to 1:5) in DMSO/H_2_O (5/95 v/v; λ_exc_ = 450 nm).

In order to further characterize the interactions between complex **3** and BSA, we monitored the evolution of the intrinsic emission band of BSA upon excitation at 290 nm, attributed to the Trp213 residue located in the hydrophobic domain IIA,^37^ in the presence of an increasing concentration of complex **3**. Briefly, the emission spectra of solutions containing 5 μM BSA dissolved in 20 mM PB buffer (pH 7.4) containing 1% DMSO incubated for 1 h at room temperature with concentrations of **3** ranging from 1 to 100 μM (Figure S33A) were recorded. A concentration-dependent decrease in the intensity of the emission band at 345 nm was observed, which suggests the quenching of BSA fluorescence by **3**. The kinetics of the intermolecular quenching processes follow the Stern–Volmer eq 2:

2 $$\frac{F_{0}}{F}=1+K_{\mathrm{sv}}\left[3\right]$$

where *F*_0_ is the fluorescence intensity of pure BSA, *F* is the fluorescence intensity after the addition of **3**, and *K*_SV_ is the Stern–Volmer dynamic quenching efficiency (Figure S33B). *K*_SV_ (eq 3) can be expressed as the product of the quenching rate *k*_q_ and the average lifetime of the fluorophore τ_0_ in the excited state, which is typically 10^–8^ s.

3 $$K_{\mathrm{SV}}=k_{q}\tau_{0}$$

The linear Stern–Volmer plot can be indicative of either a dynamic or static quenching mechanism. However, the *k*_q_ value of 2.21 ± 0.12 × 10^12^ M^–1^.s^–1^, which is higher than the maximum scattering collision quenching constant (*K*_D_ = 2.00 × 10^10^ M^–1^.s^–1^), is indicative of a static quenching mechanism.

It is further possible to determine the number of binding sites (n) and the binding constant (K_b_) by plotting log­((F_0_-F)/F) vs log­([**3**]) (Figure S33C) according to eq 4.

4 $$\log\left(\frac{F_{0}-F}{F}\right)=\log K_{b}+n\;\log\left[3\right]$$

The value of *n* = 0.9 ± 0.07 suggests that complex **3** interacts with BSA. From this graph, a *K*_b_ value of 7.73 ± 0.77 × 10^5^ M^–1^ was calculated, which indicates a strong interaction between complex **3** and BSA.

### NADH Photooxidation

NADH is a key coenzyme involved in many biocatalyzed redox reactions in living cells, and the adequate redox balance of the NADH/NAD^+^ redox couple is an important indicator of the metabolic state of the cell.^38^ Indeed, the overall in-cell NADH level is higher in cancerous cells than in normal cells to ensure high cell proliferation.^39^ Therefore, any selective catalysis of intracellular NADH oxidation to NAD^+^ by a metal complex in cancer cells can disrupt its intracellular redox balance, causing cell death.^38b,40^ The catalytic oxidation and photooxidation of NADH (2.8 mM) by **3** (0.8 mM) in DMSO/D_2_O (5/95 v/v) was monitored by ^1^H NMR spectroscopy at 298 K (Figure S34). In the dark (NADH + **3** + dark), a new set of characteristic peaks at 9.33, 9.17, 8.81, 8.48, 8.43, 8.20, 8.16, 6.04, and 5.97 ppm, ascribed to the protons of NAD^+^ (∼8%), were detected after 72 h. Light irradiation greatly accelerates the oxidation process. In fact, ∼33% of NADH was oxidized to NAD^+^ after irradiation (460 nm, 2 h), with an oxidation turnover number (TON) of 1.4 after 2 h (TOF 0.7 h^–1^). In contrast, no NAD^+^ peaks were observed for NADH alone in the dark or upon irradiation for 2 h. This study suggests that **3** could favor the endogenous oxidation of NADH to NAD^+^, both in the dark (slowly) and upon light irradiation, thus altering redox homeostasis and causing cell death. It should be noted that platinum­(II) complexes showing biocatalytic activity on the NADH/NAD^+^ redox couple are very scarce.^41^

#### Antiproliferative Studies

(C∧N) cyclometalated Pt­(II) complexes have been previously reported to have strong antiproliferative activities when associated with phenanthroline-based,^42^ diphosphine,^43^ acyclic diamino carbenes,^33b^ or picolinate^19^ ligands. In particular, (Me_2_N-pbt)-cyclometalated platinum complexes have been previously reported by some of us^24b^ to have antiproliferative properties in the micromolar range on a panel of cancer cell lines, prompting us to investigate the anticancer properties of complexes **1**–**4** in dark conditions and, considering their high quantum yield (ϕ_Δ_) of ^1^O_2_ generation, under irradiation conditions. Due to the poor solubility of the complexes in an aqueous environment, complexes **1**–**4** were first dissolved in DMSO and diluted in the culture media to reach a final concentration of 1% DMSO when incubated with the cells. All complexes were screened against the so-called “triple-negative” breast cancer cell line MDA-MB-231, lung adenocarcinoma cell line A549, and the noncancerous breast cell line MCF-10A for 72 h in dark conditions and under 5 min irradiation at 450 nm (see Experimental Section for details). The irradiation time was kept short to prevent light-induced toxicity of the energetic wavelength observed at longer irradiation times (data not shown). The measurement of the effective concentration at 50% (EC_50_) was carried out using the classical MTT assay, and the data are provided in Table 4.

**Table 4:** Effect of Compounds **1**–**4** in Dark Conditions and after 5 min Irradiation at 450 nm on Cell Viability of a Panel of Human Cancerous and Noncancerous Cell Lines after 72 h of Incubation at 37 °C^a^

	EC_50_ (μM)					
	MDA-MB-231		A549		MCF-10A	
complexes	dark	light	dark	light	dark	light
**1**	0.50 ± 0.06	0.17 ± 0.05	1.5 ± 0.1	0.57 ± 0.03	0.47 ± 0.05	0.3 ± 0.1
**2**	0.8 ± 0.2	0.26 ± 0.05	1.9 ± 0.4	1.0 ± 0.2	0.7 ± 0.2	0.4 ± 0.1
**3**	2.6 ± 0.8	0.22 ± 0.03	6.1 ± 0.2	1.10 ± 0.08	1.0 ± 0.2	0.52 ± 0.05
**4**	42.4 ± 4.2	15.9 ± 3.6	>100	>100	21.7 ± 4.2	15.0 ± 0.9
**Cisplatin** ^b^	20.4 ± 3.4		1.7 ± 0.5		2.9 ± 0.8	

a Each data represents the average of three experiments ± standard error.

b Values taken from ref 44.^44^

In dark conditions, we could notice a strong influence of substituents on the picolinate ligands. Indeed, the presence of a carboxylic acid substituent on the picolinate ligand led to a reduction in the activity of the complex (EC_50_(MDA-MB-231) = 42.4 ± 4.2 and 0.50 ± 0.06 μM for complexes **4** and **1**, respectively, and EC_50_(A549) > 100 and 1.5 ± 0.1 μM for complexes **4** and **1**, respectively). This can be correlated with its poor stability in the culture medium mentioned above, which might reduce its cellular uptake. On the other hand, introduction of an amino group on picolinate did not reduce the activity of the complexes (EC_50_(MDA-MB-231) = 0.8 ± 0.2 and 0.50 ± 0.06 μM for **2** and **1**, respectively, and EC_50_(A549) = 1.9 ± 0.4 and 1.5 ± 0.1 μM for **2** and **1**, respectively). On the contrary, introduction of a hydroxy group on the picolinate ligand led to almost 5-times reduction of the antiproliferative activity in dark conditions against both cancerous cell lines (EC_50_(MDA-MB-231) = 2.6 ± 0.8 and 0.50 ± 0.06 μM for **3** and **1**, respectively, and EC_50_(A549) = 6.1 ± 0.2 and 1.5 ± 0.1 μM for **3** and **1**, respectively). Moreover, complexes **1**–**3** appeared 8 and 40 times more active than cisplatin against the “triple-negative” breast cancer cells MDA-MB-231. However, in dark conditions, no particular selectivity for cancer cells could be observed with similar activity measured against the noncancerous cell line MCF-10A. For the “light” experiments, the cells were preincubated with the complexes for 4 h in the dark, irradiated at 450 nm for 5 min, and then put back in the dark until 72 h. The photoactivity index (PI) values, defined as the ratio between the EC_50_ in dark conditions and the EC_50_ after irradiation for each cell line, are shown in Figure 10.

**Figure 10:**
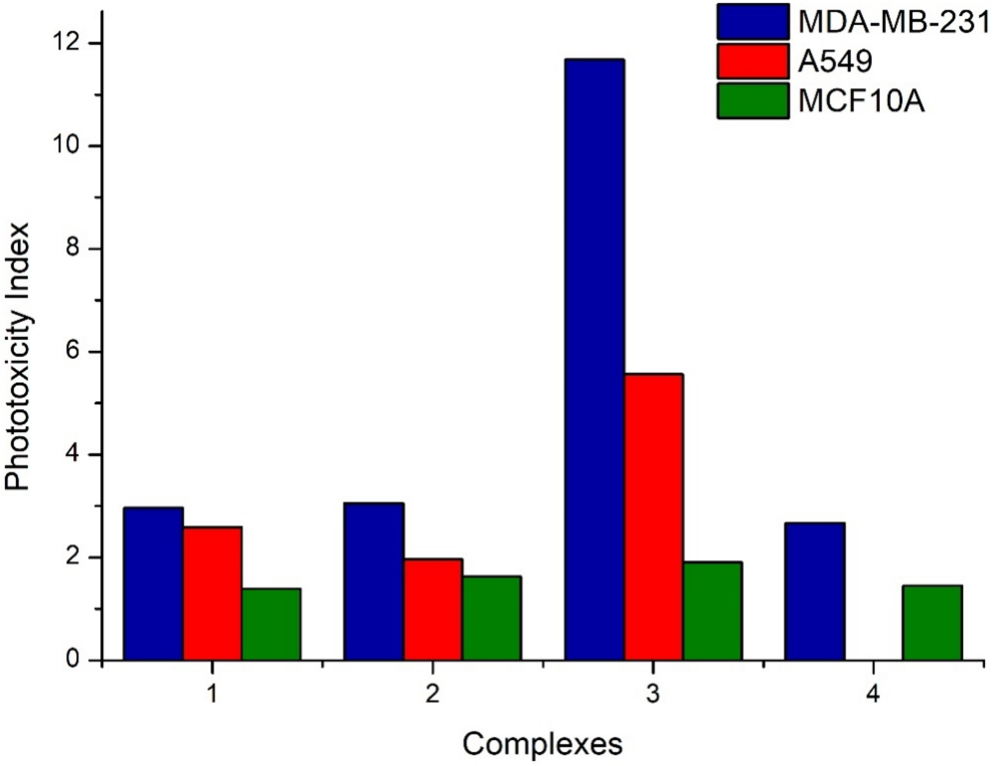
PI values of complexes **1**–**4** for MDA-MB-231, A549, and MCF-10A cells upon 5 min of irradiation at 450 nm.

Similar to the dark experiments, complex **4** appeared the least active on all cell lines with very limited phototoxicity, which might be rationalized considering its poor stability in DMEM. Interestingly, after irradiation, complexes **1**–**3** demonstrated similarly strong activities in the low to submicromolar range. As such, complexes **1** and **2**, which were already highly active in the dark, demonstrated only limited phototoxic properties (PI values between 2 and 3 for both cancer cell lines); whereas, complex **3** with a hydroxy substituent on picolinate demonstrated the highest photoactivity with PI values of 11.7 and 5.6 on MDA-MB-231 and A549 cells, respectively, associated with a limited phototoxic activity on the noncancerous cell line (PI < 2). The higher phototoxic activity of complex **3** resulted in much improved selectivity for cancer cells. Indeed, while in dark conditions, **3** showed a 2-fold lower activity toward MDA-MB-231 cells compared to the noncancerous cells upon irradiation, complex **3** appeared twice as active on the same cancer cell line compared to the noncancerous one.

#### Mechanistic Studies

Platinum­(II) complexes, such as cisplatin, presenting exchangeable ligands are well known to interact with puric bases of DNA, leading to the formation of intra- and interstrand cross-links and in turn to transcription inhibition, cell cycle arrest, and ultimately cell death.^45^ Based on the reactivity in the cell culture medium, the interaction of complexes **1**–**4** and cisplatin with CT-DNA was investigated by circular dichroism (CD) spectroscopy after incubation at 37 °C for 24 h in the dark or for 4 h incubation and 5 min irradiation at 450 nm, followed by 20 h incubation, following a reported protocol.^46^ The CD spectrum of CT-DNA presents a positive peak at 280 nm, assigned to base stacking, and a negative peak at 245 nm, assigned to helicity.^47^ While incubation with cisplatin led to a decrease of the band at 245 nm and an increase of the band at 280 nm in agreement with literature data,^46,47^ incubation with complexes **1**–**4** did not lead to modifications of the CD spectrum of CT-DNA in both dark and irradiation conditions, suggesting that their mechanism of action might not involve cisplatin-like DNA interactions (Figure S35) in contrast to cyclometalated Pt­(II) complexes presenting chloride and acyclic diaminocarbene ligands.^33b^

Cyclometalated complexes of various metals, including Ir­(III),^48^ Rh­(III),^49^ Pt­(II),^50^ and Au­(III),^51^ have been reported to induce overproduction of reactive oxygen species (ROS), inducing oxidative stress leading to cell death. Intracellular ROS production in MDA-MB-231 cells was investigated with complex **3**, which showed the highest phototoxicity index. Following the protocol used for cytotoxicity assays, cells were incubated with **3** for 4 h in the dark at 37 °C (dark conditions) or incubated with **3** for 4 h in the dark at 37 °C, followed by 5 min of irradiation at 450 nm (irradiation conditions). ROS production was measured using the nonspecific ROS probe 2′,7′-dichlorodihydrofluorescein diacetate (H_2_DCFDA) (Figures 11 and S36).

**Figure 11:**
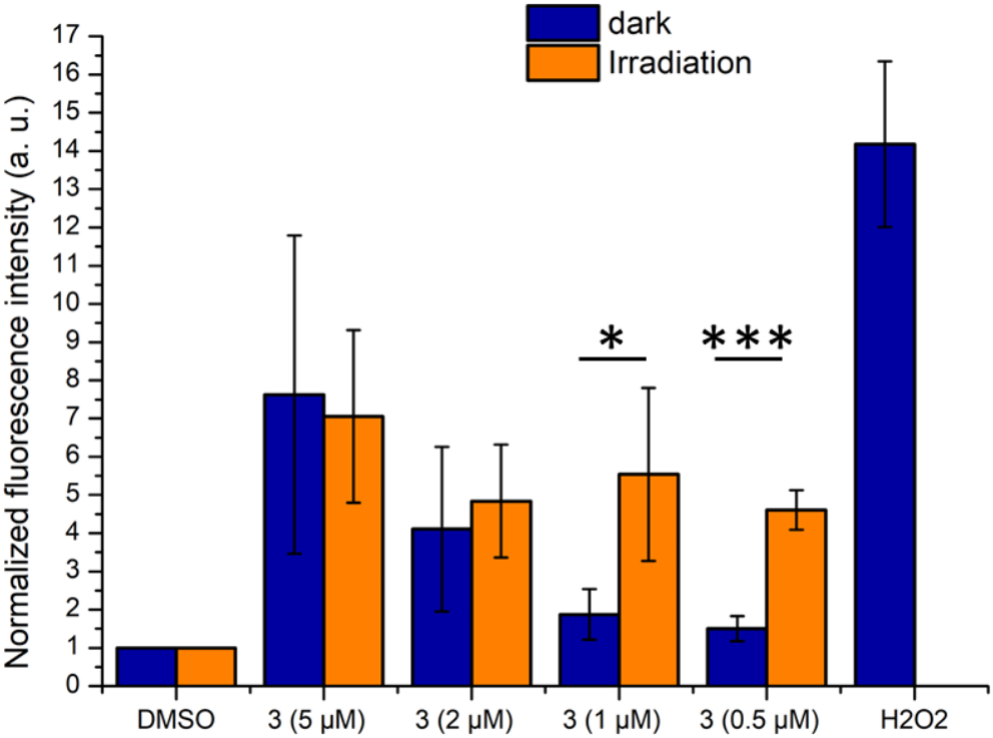
ROS production upon incubation of MDA-MB-231 cells for 4 h at 37 °C (dark) or for 4 h at 37 °C + 5 min irradiation at 450 nm (irradiation) with complex **3** (5, 2, 1, or 0.5 μM), expressed as the mean fluorescence intensity normalized to DMSO negative controls. Each data point represents the average of three experiments ± standard deviation. Statistical analysis was performed using the Student’s *t-*test. *p ≤ 0.05 and ***p ≤ 0.001.

In dark conditions, complex **3** induces a dose-dependent increase of green fluorescence, indicative of dose-dependent ROS production, in good agreement with its low-micromolar anticancer activity and NADH oxidation. Upon irradiation at 450 nm, a statistically significant increase of ROS production was observed at the lowest concentrations (0.5 and 1 μM). This increase of ROS production is in good agreement with its enhanced phototoxicity and faster NADH oxidation. Altogether, these data suggest that complex **3** can chemically induce ROS production, which can be further stimulated upon blue light irradiation, similar to that observed previously for luminescent Ir­(III) complexes.^48b^

## Conclusions

The synthesis and detailed photophysical study were undertaken for four (Me_2_N-pbt) cyclometalated Pt­(II) complexes containing picolinate ligands (**1**- **4**). In agreement with the color of solids **1** (orange) and **3** (black), their crystal packing shows extensive intermolecular π···π (**1**) or Pt···^.^Pt and π···π (**3**·CHCl_3_) interactions, thus generating 1D stacking. This fact is reflected in the emission of **4** in the solid state at 298 K, which shows the dual emission of ^3^LL′CT/^3^ILCT (630 nm) and ^3^MM­(L+L′)­CT in the NIR (860 nm) (L = Me_2_N-pbt, L′ = pic). DFT calculations allowed us to assign the vibronic structured phosphorescence emission bands in **1**–**3** to ^3^ILCT and in **4** to ^3^LL′CT/^3^ILCT. In solution at room temperature (CH_2_Cl_2_ **1**–**3**, DMSO **4**), they display dual emission, fluorescence [^1^(M + L)­L′CT (**1**–**3**) and ^1^LL′CT/^1^ILCT or ^1^(M + L)­L′CT (**4**), depending on the excitation wavelength], and phosphorescence. In aerated DMSO solutions and under continuous photoexcitation at 365 nm, the phosphorescence of **1**–**3** is photoinduced, and their triplet states efficiently sensitize molecular oxygen with values ranging from 13.6 to 17.4%. The aggregation quenching effect due to molecular aggregation of **1** and **3** was evidenced in PS films by increasing the doping concentration, and for **3** in DMSO–H_2_O mixtures with a high content of H_2_O. While all complexes appeared stable in DMSO, DMSO/water, and PBS/DMSO mixtures, they underwent transformations in cell culture medium, with rates depending on the substituents of the picolinate ligand, and complexes **2** and **3** being the most stable. Experimental studies indicate that complex **3** catalyzes the photooxidation of NADH to NAD^+^ and binds strongly to BSA, with only one binding site. Besides, the *in vitro* cytotoxicity of the four complexes toward triple-negative breast cancer cells MDA-MB-231, A549 lung adenocarcinoma, and noncancerous breast cells MCF-10A in dark conditions and under irradiation (450 nm, 5 min) was measured, demonstrating a promising phototoxicity against MDA-MB-231 cells for complex **3**, with a 12-fold increase of the anticancer activity upon irradiation associated with an enhancement of the selectivity for the cancer cells. Mechanistic studies suggested that the complexes do not bind to DNA as cisplatin does, but could trigger the overproduction of ROS in the dark, which is further enhanced upon irradiation. Although the phototoxicity index remains to be improved, it opens the way for the development of nontoxic PDT agents for the treatment of “triple-negative” breast cancer.

## Experimental Section

### General Comments

All reactions were carried out under a dry argon atmosphere using standard Schlenk techniques. The solvents were obtained from a solvent purification system (M-BRAUN MS SPS-800). Elemental analyses were carried out with a Leco TruSpec Micro CHNS microanalyzer. Mass spectra were recorded with electrospray ionization on an interphase ESI/APCI Bruker Microtof-Q spectrometer with positive ion mode, with MeOH/H_2_O 90/10 and 0.1% formic acid as a mobile phase. IR spectra of the powders were obtained on a PerkinElmer Spectrum UATR Two FT-IR spectrophotometer, with the diamond crystal ATR accessory, covering the region between 4000 and 450 cm^–1^; data processing was carried out with an Omnic. NMR spectra were recorded on a Bruker AVANCE ARX 300 or 400 spectrometer at 298 K. Chemical shifts are reported in parts per million (ppm) relative to external standards (SiMe_4_ for ^1^H and ^13^C­{^1^H}), and all coupling constants are given in hertz (Hz). The UV–vis absorption spectra were measured with a Hewlett-Packard 8453 spectrophotometer. Diffuse reflectance UV–vis (DRUV) spectra were obtained with SiO_2_ pellets, using a Shimadzu UV-3600 spectrophotometer with a Harrick Praying Mantis accessory, and recalculated following the Kubelka–Munk function. Excitation and emission spectra were obtained at room temperature with an Edinburgh FLS 1000 spectrofluorimeter and at 77K with a Shimadzu RF-60000 spectrofluorimeter. Lifetime measurements on the solid state and PS films (>10 μs) were performed with an Edinburgh FLS 1000 spectrofluorimeter with a μF2 pulse lamp (power: 100 W, fuse: 3.15 Amp A/S). The lifetimes below 10 μs at 298 K were measured with a Datastation HUB-B with a nanoLED controller using the technique “time correlated single photon counting” (TCSPC). The nanoLEDs employed for lifetime measurements were 390 nm with pulse lengths of 1.2 ns. The decay data were analyzed using the software DAS6 (Jobin Yvon-Horiba). The absolute quantum yields were determined with a Hamamatsu Absolute PL Quantum Yield Measurement System. The starting material [Pt­(Me_2_N-pbt)­Cl­(DMSO)][Bibr cit24a] was prepared according to a published procedure. All other commercially available reagents were used as received.

#### Synthesis of [Pt­(Me_2_N-pbt)­(pic-κ*N,O*)] (**1**)

A mixture of [Pt­(Me_2_N-pbt)­Cl­(DMSO)] (0.121 g, 0.215 mmol), 2-picolinic acid (0.027 g, 0.215 mmol), and an excess of Na_2_CO_3_ (∼0.25 g) in 20 mL of acetone was stirred for 5 h. The resulting suspension was evaporated to dryness, and the residue was treated with CH_2_Cl_2_ (30 mL) and deionized water (30 mL). The organic phase was extracted, dried with anhydrous MgSO_4_, and filtered through Celite. The filtrate was dried, and the residue was treated with *n*-hexane (10 mL) to give **1** as an orange solid (0.099 g, 81%). Anal. Calcd for C_21_H_17_N_3_O_2_PtS (570.073): C, 44.21; H, 3.00; N, 7.37; S, 5.62. Found: C, 44.02; H, 3.21; N, 7.14; S, 5.49%. ESI (+): *m*/*z* (%): 571.08 [M + H]^+^ (100). IR (ATR) (cm^–1^): ν­(C=O) 1661 (s), ν­(C–O) 1336 (s). ^1^H NMR (400 MHz, CDCl_3_): δ = 9.29 (d, ^*3*^ *J*_*H–H*_ = 5.2, ^*3*^ *J*_*Pt–H*_ = 39, H^6′^), 9.08 (d, ^*3*^ *J*_*H–H*_ = 8.4, H^7^), 8.21 (d, ^*3*^ *J*_*H–H*_ = 7.5, H^4^), 8.14 (t, ^*3*^ *J*_*H–H*_ = 7.5, H^5^), 7.70 (d, ^*3*^ *J*_*H–H*_ = 8, H^3′^), 7.61 – 7.54 (m, 2H, H^5′,6^), 7.36 (t, ^*3*^ *J*_*H–H*_ = 8, H^4′^), 7.33 (d, ^*3*^ *J*_*H–H*_ = 8, H^8^), 6.58 (s, ^*3*^ *J*_*Pt–H*_ = 41, H^11^), 6.46 (d, ^*3*^ *J*_*H–H*_ = 8, H^9^), 3.11 (s, 6H, -NMe_2_).

#### Synthesis of [Pt­(Me_2_N-pbt)­(3-NH_2_-pic-κ*N,O*)] (**2**)

Complex **2** was obtained as an orange solid (0.091 g, 70%), following the same procedure as that for **1**, starting from [Pt­(Me_2_N-pbt)­Cl­(DMSO)] (0.126 g, 0.224 mmol) and 3-amine-2-picolinic acid (0.031 g, 0.224 mmol). Anal. Calcd for C_21_H_18_N_4_O_2_PtS (585.08): C, 43.08; H, 3.10; N, 9.57; S, 5.48. Found: C, 43.15; H, 3.01; N, 9.41; S, 5.23%. ESI (+): *m*/*z* (%): 586.09 [M + H]^+^ (100), 840.18 [M+Me_2_N-pbt+H]^+^ (52%). IR (ATR) (cm^–1^): ν­(N–H) 3383 (s), ν­(C=O) 1580 (s), and ν­(C–O) 1357 (s). ^1^H NMR (400 MHz, CDCl_3_): 9.11 (d, ^*3*^ *J*_*H–H*_ = 8.2, H^7^), 8.58 (d, ^*3*^ *J*_*H–H*_ = 5.1, ^*3*^ *J*_*Pt–H*_ = 46.5, H^6′^), 7.70 (d, ^*3*^ *J*_*H–H*_ = 7.9, H^4^), 7.57 (t, ^*3*^ *J*_*H–H*_ = 7.9, H^6^), 7.38 (d, ^*3*^ *J*_*H–H*_ = 8.5, H^8^), 7.33 (t, ^*3*^ *J*_*H–H*_ = 7.9, H^5^), 7.29 (d, ^*3*^ *J*_*H–H*_ = 8.3, H^4′^), 7.21 (dd, ^*3*^ *J*_*H–H*_ = 5.1, ^*3*^ *J*_*H–H*_ = 8.3, H^5′^), 6.61 (d, ^*4*^ *J*_*H–H*_ = 1.6, ^*3*^ *J*_*Pt–H*_ = 41.1, H^11^), 6.47 (d, ^*3*^ *J*_*H–H*_ = 8.5, ^*4*^ *J*_*H–H*_ = 1.6, H^9^), 6.43 (s_*br*_, 2H, -NH_2_), 3.10 (s, 6H, -NMe_2_). ^13^C­{^1^H} NMR (100.6 MHz, CDCl_3_): δ = 180.9 (s, C^2^) 176.6 (s, C=O), 151.4 (s, C^2′^), 150.4 (s, C^7a^), 147.8 (s, C^12^), 139.5 (s, C^10^), 139.2 (s, C^6′^), 132.9 (s, C^3′^), 129.6 (s, C^3a^), 129.5 (s, C^13^), 128.1 (s, C^6^), 127.4 (s, C^8^), 127.3 (s, C^5′^), 126.4 (s, C^4′^), 124.6 (s, C^5^), 121.7 (s, C^4^), 121.1 (s, C^7^), 115.1 (s, C^11^), 107.4 (s, C^9^), 40.3 (s, -NMe_2_).

#### Synthesis of [Pt­(Me_2_N-pbt)­(3-OH-pic-κ*N,O*)] (**3**)

Complex **3** was obtained as an orange solid (0.124 g, 72%), following the same procedure as for **1**, starting from [Pt­(Me_2_N-pbt)­Cl­(DMSO)] (0.164 g, 0.292 mmol) and 3-hydroxy-2-picolinic acid (0.041 g, 0.292 mmol). Anal. Calcd for C_21_H_17_N_3_O_3_PtS (586.06): C, 43.00; H, 2.92; N, 7.16; S, 5.47. Found: C, 43.23; H, 3.12; N, 7.04; S, 5.52%. ESI (+): *m*/*z* (%): 587.07 [M + H]^+^ (100). IR (ATR) (cm^–1^): ν­(O–H) 3084 (s), ν­(C=O) 1640 (s), ν­(C–O) 1326 (s). ^1^H NMR (400 MHz, CDCl_3_): 13.21 (s, −OH), 8.86 (d, ^*3*^ *J*_*H–H*_ = 8.3, H^7^), 8.74 (d, ^*3*^ *J*_*H–H*_ = 5, ^*3*^ *J*_*Pt–H*_ = 38, H^6′^), 7.66 (d, ^*3*^ *J*_*H–H*_ = 7.9, H^4^), 7.59 (t, ^*3*^ *J*_*H–H*_ = 8.3, H^4′^), 7.48 (d, ^*3*^ *J*_*H–H*_ = 7.9, H^6^), 7.38 (dd, ^*3*^ *J*_*H–H*_ = 8.3, ^*3*^ *J*_*H–H*_ = 5, H^5′^), 7.30 (t, ^*3*^ *J*_*H–H*_ = 7.9, H^5^), 7.23 (d, ^*3*^ *J*_*H–H*_ = 8.6, H^8^), 6.49 (d, ^*4*^ *J*_*H–H*_ = 1.3, ^*3*^ *J*_*Pt–H*_ = 38.2, H^11^), 6.31 (d, ^*3*^ *J*_*H–H*_ = 8.6, ^*4*^ *J*_*H–H*_ = 1.3, H^9^), 3.06 (s, 6H, -NMe_2_). ^13^C­{^1^H} NMR (100.6 MHz, CDCl_3_): δ = 180.9 (s, C^2^) 176.6 (s, C=O), 151.4 (s, C^2′^), 150.4 (s, C^7a^), 147.8 (s, C^12^), 139.5 (s, C^10^), 139.2 (s, C^6′^), 132.9 (s, C^3′^), 129.6 (s, C^3a^), 129.5 (s, C^13^), 128.1 (s, C^6^), 127.4 (s, C^8^), 127.3 (s, C^5′^), 126.4 (s, C^4′^), 124.6 (s, C^5^), 121.7 (s, C^4^), 121.1 (s, C^7^), 115.1 (s, C^11^), 107.4 (s, C^9^), 40.3 (s, -NMe_2_).

#### Synthesis of [Pt­(Me_2_N-pbt)­(4-COOH-pic-κ*N,O*)] (**4**)

Pyridin-2,4-dicarboxylic acid (0.043 g, 0.255 mmol) was added to a yellow solution of [Pt­(Me_2_N-pbt)­Cl­(DMSO)] (0.143 g, 0.255 mmol) in toluene (20 mL). After 8 h of refluxing, the suspension was filtered and washed with hexane (10 mL) to obtain complex **4** as a black solid (0.954 g, 61%). Anal. Calcd for C_22_H_17_N_3_O_4_PtS (614.06): C, 43.00; H, 2.79; N, 6.84; S, 5.22. Found: C, 43.20; H, 2.53; N, 6.69; S, 5.10%. ESI (+): *m*/*z* (%): 615.07 [M + H]^+^ (100); ESI(−): *m*/*z* (%): 613.00 [M-H]^−^ (100). IR (ATR) (cm^–1^): ν­(O–H) 3455 (s), ν­(C=O) 1584 (s), ν­(C–O) 1363 (s). ^1^H NMR (400 MHz, DMSO-d^6^): δ = 9.46 (d, ^*3*^ *J*_*H–H*_ = 6.0, H^6′^), 8.84 (d, ^*3*^ *J*_*H–H*_ = 8.0, H^7^), 8.31 – 8.26 (m, 2H, H^3′,5′^), 8.07 (d, ^*3*^ *J*_*H–H*_ = 8.0, H^4^), 7.59 (td, ^*3*^ *J*_*H–H*_ = 8.0, ^*4*^ *J*_*H–H*_ = 1.0, H^6^), 7.51 (d, ^*3*^ *J*_*H–H*_ = 8.0, H^8^), 7.43 (td, ^*3*^ *J*_*H–H*_ = 8.0, ^*4*^ *J*_*H–H*_ = 1.0, H^5^), 6.57 – 6.52 (m, 2H, H^9,11^), 3.13 (s, 6H, -NMe_2_).

### X-ray Structure Determination

X-ray crystallographic data and selected bond lengths and angles for **1** and **3·CHCl**_**3**_ are summarized in Tables S1 and S2. Orange and black crystals of **1** and **3·CHCl**_**3**_were obtained by slow diffusion of *n*-hexane into a solution of the corresponding complex in CHCl_3_ at room temperature (**1**) or low temperatures (**3·CHCl**_**3**_). X-ray intensity data were collected using molybdenum graphite monochromatic (Mo–Kα) radiation with a Bruker APEX-II diffractometer at a temperature of 120 K with an Oxford Cryosystem temperature controller using the APEX-II software. Structures were solved by Intrinsic Phasing using SHELXT^52^ with the WinGX graphical user interface.^53^ Multiscan absorption corrections were applied to all the data sets and refined by full-matrix least squares on *F* ^*2*^ with SHELXL.^54^ Hydrogen atoms were positioned geometrically, with isotropic parameters *U*_*iso*_ *= 1.2 U*_*eq*_ (parent atom) for aromatic hydrogens and CH_2_ and *U*_*iso*_ *= 1.5 U*_*eq*_ (parent atom) for methyl groups. Finally, the structures show some residual peaks greater than 1 e Å^–3^ in the vicinity of the platinum atoms, but with no chemical meaning.

### Theoretical Calculations

Calculations were carried out with the Gaussian 16 package^55^ for **1**–**4**, using Becke′s three-parameter functional combined with Lee–Yang–Parr′s correlation functional (B3LYP)^56^ and the Becke-Johnson D3BJ correction.^57^ Optimizations on the singlet state (S_0_) were performed using as a starting point the molecular geometry obtained through X-ray diffraction analysis for all complexes. No negative frequency was observed in the vibrational frequency analysis of the final equilibrium geometries. The basis set used was the LanL2DZ effective core potential for Pt and 6–31G­(d,p) for the ligand atoms.^58^ DFT and TD-DFT calculations were carried out using the polarized continuum model approach^59^ (PCM) implemented in the Gaussian 16 software. The predicted emission wavelengths were obtained by the energy difference between the triplet state at its optimized geometry and the singlet state at the triplet geometry. The results were visualized with GaussView 6. Overlap populations between molecular fragments were calculated using the GaussSum 3.0 software.^60^

### General Procedure of Stability and Photostability by NMR Spectroscopy

Complexes were dissolved in DMSO-d^6^ (300 μL, 1.5 × 10^–3^ M) or in DMSO-d^6^/D_2_O (9/1, complexes were dissolved first in DMSO-d^6^, and water was added), and ^1^H NMR spectra were recorded from 0 to 72 h in a 400 MHz spectrometer. Photostability in DMSO-d^6^ and DMSO-d^6^/D_2_O: complexes were dissolved in DMSO-d^6^ (300 μL, 1.5 × 10^–3^ M) or in DMSO-d^6^/D_2_O (9/1) and the ^1^H NMR spectra were recorded after 15 and 30 min of irradiation at 298 K with λ = 396 nm (15 W) in a 400 MHz spectrometer.

### Stability in PBS and Serum-Free DMEM

The UV–vis spectra of complexes **1**–**4** were recorded in PBS:DMSO (95/5) and serum-free DMEM:DMSO (95/5) at a concentration of 25 μM on a Cary 50 spectrophotometer (Varian).

### BSA Binding

For the BSA binding experiment, solutions of complex **3** with BSA in mixtures of DMSO/H_2_O (5/95 v/v) were prepared, keeping the concentration of **3** (10 μM) constant and increasing the amount of BSA (from 0 to 50 μM). Qualitative analyses of the interaction between **3** and BSA were performed by fluorometric measurements. For each addition, the mixing solution was stirred for 5 min and allowed to stand for 5 min. The fluorescence intensities were then measured at λ_exc_ = 450 nm, and the emission fluorescence spectra were recorded in the wavelength range λ = 400–800 nm.

For the determination of the number of complexes **3** interacting with BSA and K_b_ calculations, BSA was dissolved at a concentration of 5 μM in 20 mM PB buffer (pH 7.4) containing 1% DMSO. BSA was incubated for 1 h at room temperature alone or in the presence of 1 to 100 μM of complex **3.** The fluorescence spectra of BSA (λ_exc_ = 290 nm, λ_em_ = 345 nm) were recorded using a FP-6200 fluorimeter (Jasco).

### NADH Photooxidation

The time dependence of the catalytic oxidation and photooxidation of complex **3** (0.8 mM) with NADH (2.8 mM) in DMSO/D_2_O (5/95 v/v) was studied for 72 h in the dark or 2h with light irradiation (λ_exc_ = 460 nm) by ^1^H NMR at 298 K. The conversion of NADH to NAD^+^ was approximately calculated from the NMR data.

### Cell Culture and Cell Growth Inhibition

The human lung adenocarcinoma cell line A549 and human breast cancer cell line MDA-MB-231 were cultivated in DMEM (Dulbecco’s modified Eagle’s medium) containing GlutaMax I supplemented with 10% FBS at 37 °C in a humidified atmosphere and 5% CO_2_. The noncancerous cell line MCF-10A was maintained in DMEM:F12 (1:1) cell culture media, 5% heat-inactivated horse serum, supplemented with HEPES (20 mM), l-glutamine (2 mM), epidermal growth factor (20 ng/mL), hydrocortisone (500 ng/mL), cholera toxin (100 ng/mL), and insulin (10 μg/mL). Cell viability was evaluated by using a colorimetric method based on the tetrazolium salt MTT [3-(4,5-dimethylthiazol-2-yl)-2,5-diphenyltetrazolium bromide], which is reduced by viable cells to yield purple formazan crystals. Cells were seeded in 96-well plates at a density of 40000 cells/mL (100 μL per well). Stock solutions were prepared in DMSO for complexes **1–4** and in water for cisplatin. The percentage of DMSO in the culture medium did not exceed 1%. For dark experiments, after overnight attachment, a dilution series of the compounds was added to the medium, and the cells were incubated for a further 72 h. For the irradiated experiments, after overnight attachment, a dilution series of the compounds was added to the medium, and the cells were preincubated for 4 h. The cells were then irradiated at 450 nm for 5 min with a Kessil Lamp (power: 3 mW/cm^2^, 1.13 × 10^–8^ Einstein/s at 20 cm distance between the light source and 96-well plate) and the incubation was continued until 72 h. After 72 h, the medium was removed and the cells were incubated with MTT solution in PBS (10 μL of 5 mg/mL) for 2–3 h. The formed purple formazan crystals were dissolved in 100 μL of DMSO by thorough shaking, and the absorbance at 580 nm was read using a microplate reader (FLUOstar OPTIMA, BMG Labtech). Each test was performed with at least 3 replicates and repeated at least 3 times. The EC_50_ values were determined using GraphPad Prism 8.0 software.

### Interaction of Complexes **1**–**4** with CT-DNA by Circular Dichroism

A stock solution of calf thymus DNA (CT-DNA) was prepared in 5 mM TRIS·HCl, and 50 mM NaCl (pH 7.4). The concentration of nucleotides was assayed at 260 nm using ε = 6600 M^–1^.cm^–1^ (*c* = 3.38 mM). Mixtures of CT-DNA (50 μM) and complexes (50 μM, i.e. Pt/nucleotide = 1) in 10 mM PB (pH 7.4) containing 10% DMSO were incubated for 24 h at 37 °C in the dark or 4 h in the dark, followed by 5 min irradiation at 450 nm with a Kessil lamp (power: 3 mW/cm^2^, 1.13 × 10^–8^ Einstein/s at 20 cm distance between the light source and samples), and 20 h in the dark again. As a positive control, a mixture of CT-DNA (50 μM) and cisplatin (50 μM, i.e., Pt/nucleotide = 1) in 10 mM PB (pH 7.4) was incubated at 37 °C for 24 h in the dark. Circular dichroism spectra (238–340 nm, 5 scans) were recorded in a 1 cm path length cell on a J-815 CD spectrometer (JASCO) at 20 °C with 0.1 nm data pitch, 50 nm/min scan speed, and 1 nm bandwidth.

### Measurement of Reactive Oxygen Species (ROS)

Triple-negative breast cancer cells MDA-MB-231 were seeded in 6-well plates at a concentration of 150 000 cells/well in 1.5 mL DMEM (Dulbecco’s modified Eagle’s medium) containing GlutaMax I supplemented with 10% FBS. After an overnight recovery at 37 °C and 5% CO_2_, the cells were incubated in the dark with complex **3** at 0.5, 1, 2, or 5 μM for 4 h at 37 °C and 5% CO_2_. Negative controls were prepared with 1% DMSO (vehicle solvent). After incubation, cells were irradiated for 5 min at 450 nm using a Kessil lamp (power: 3 mW/cm^2^, 1.13 × 10^–8^ Einstein/s at 20 cm distance between the light source and 6-well plate) (light samples) or not irradiated (dark samples). For both light and dark samples, the culture medium was removed, the cells were washed with 2 × 1 mL of HBSS buffer, and 1.5 mL of HBSS buffer was added to each well. The fluorogenic ROS probe 2′,7′-dichlorodihydrofluorescein diacetate (H_2_DCFDA, TCI Chemicals) was added at a concentration of 10 μM to each well (15 μL of a 1 mM stock solution in DMSO). Positive controls were prepared by simultaneous addition of 100 μM H_2_O_2_ to H_2_DCFDA. The cells were incubated for 1 h in the dark at 37 °C and 5% CO_2_. Microscopy images were recorded in brightfield and GFP filter modes using an EVOS M5000 microscope with a 10× objective. Cell confluency was assessed on the brightfield images using EVOS M5000 microscope software, and quantification of green fluorescence was performed using Fiji software. After correction of the fluorescence according to confluency, results are presented relative to the negative controls.

## Supplementary Material



## Notes

Characterization of complexes (NMR spectra, crystal data), photophysical properties, computational details, and biological and stability studies (PDF)
