# Supplementary material for: Structural Features and Photophysical and Antiproliferative Properties of Me2N‑pbt-Cycloplatinated Complexes with Picolinate Ligands
Source: Inorg Chem. 2025 May 13;64(20):10243–59. doi: 10.1021/acs.inorgchem.5c01175 (PMC12176268; doi:10.1021/acs.inorgchem.5c01175)
Supplement: Supplementary file 1 [file ic5c01175_si_001.pdf]

Supporting Information

Structural Features, Photophysical and Antiproliferative Properties of Me_2N-pbt-Cycloplatinated Complexes with Picolinate ligands

David Gómez de Segura,[a] Michèle Salmain,[b] Benoît Bertrand,[b] Julio Fernández-Cestau,[a] Elena Lalinde*[a] and M. Teresa Moreno*[a]*

[a] Departamento de Química-Instituto de Investigación en Química (IQUR), Universidad de La Rioja, 26006, Logroño, Spain. E-mail: elena.lalinde@unirioja.es; teresa.moreno@unirioja.es

[b] Sorbonne Université, CNRS, Institut Parisien de Chimie Moléculaire (IPCM UMR 8232), F-75005 Paris, France. Email: benoit.bertrand@sorbonne-universite.fr

Contents: Page

1.- NMR Spectra……………………………………………………………………….. S2
2.- Crystal Structures……………………………………………………………….. S6
3.- Photophysical Properties and Theoretical calculations………………………….. S10
4.- Biological Properties…………………………………………………………….. S32

1.- NMR Spectra

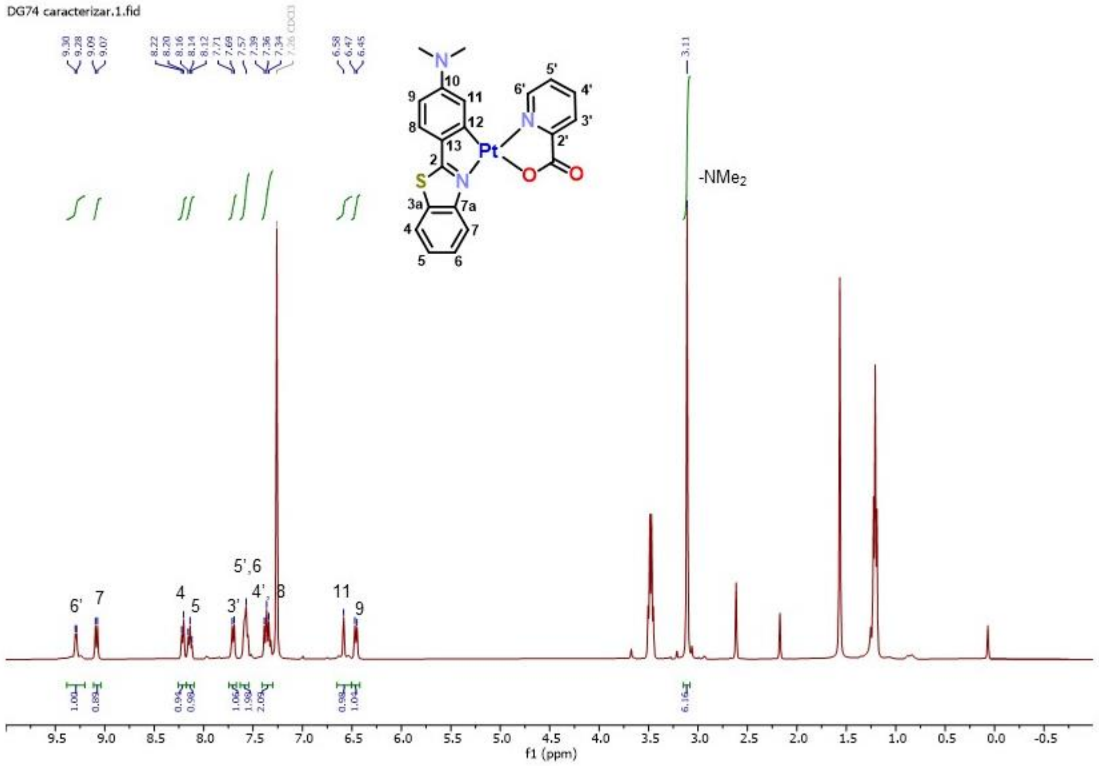

Figure S1. ^{1}H NMR spectra of **1** in $CDCl_3$ at 298 K

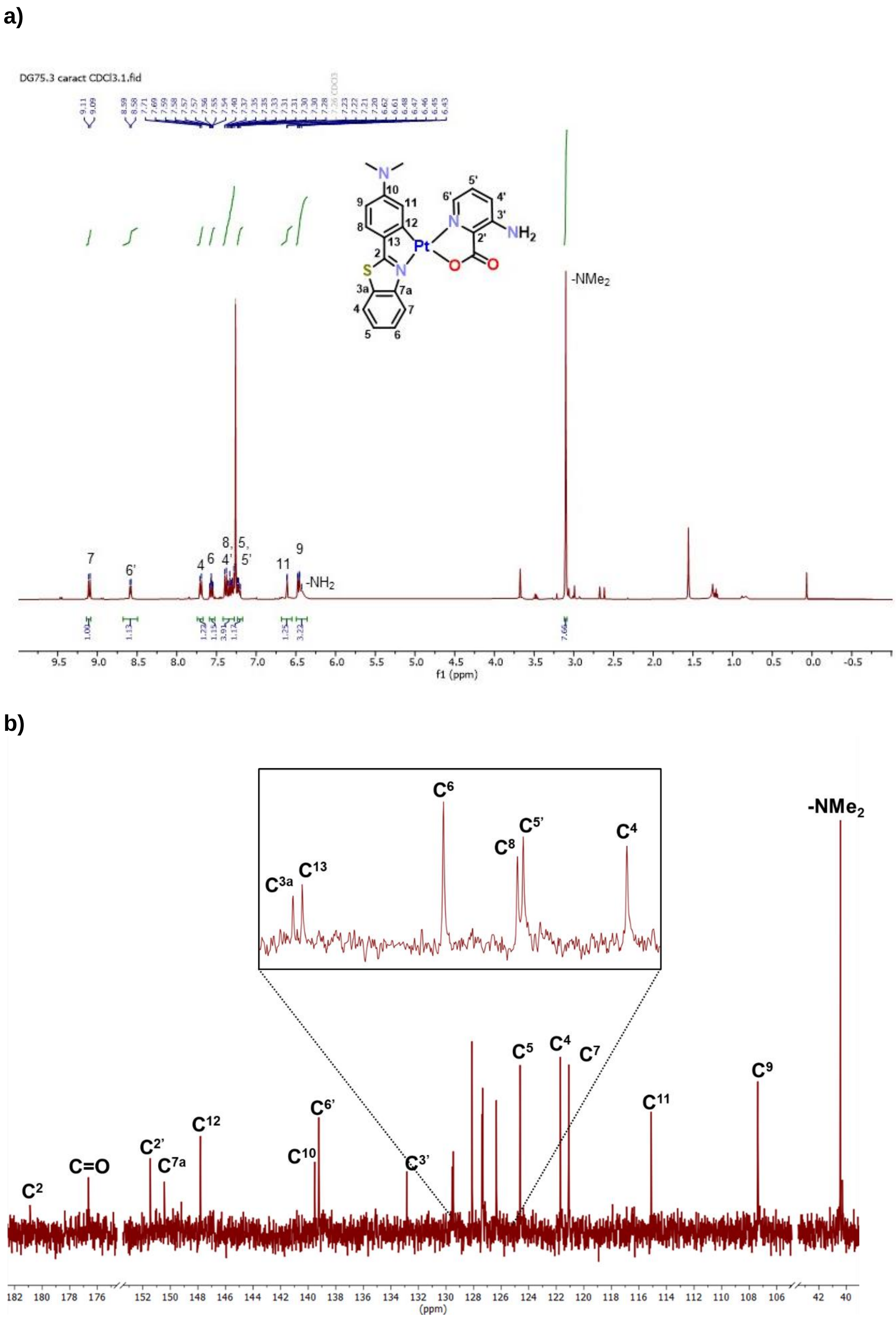

Figure S2. NMR spectra of **2** in $CDCl_3$ at 298 K (a) ^1H, (b) $^{13}C\{^1H\}$

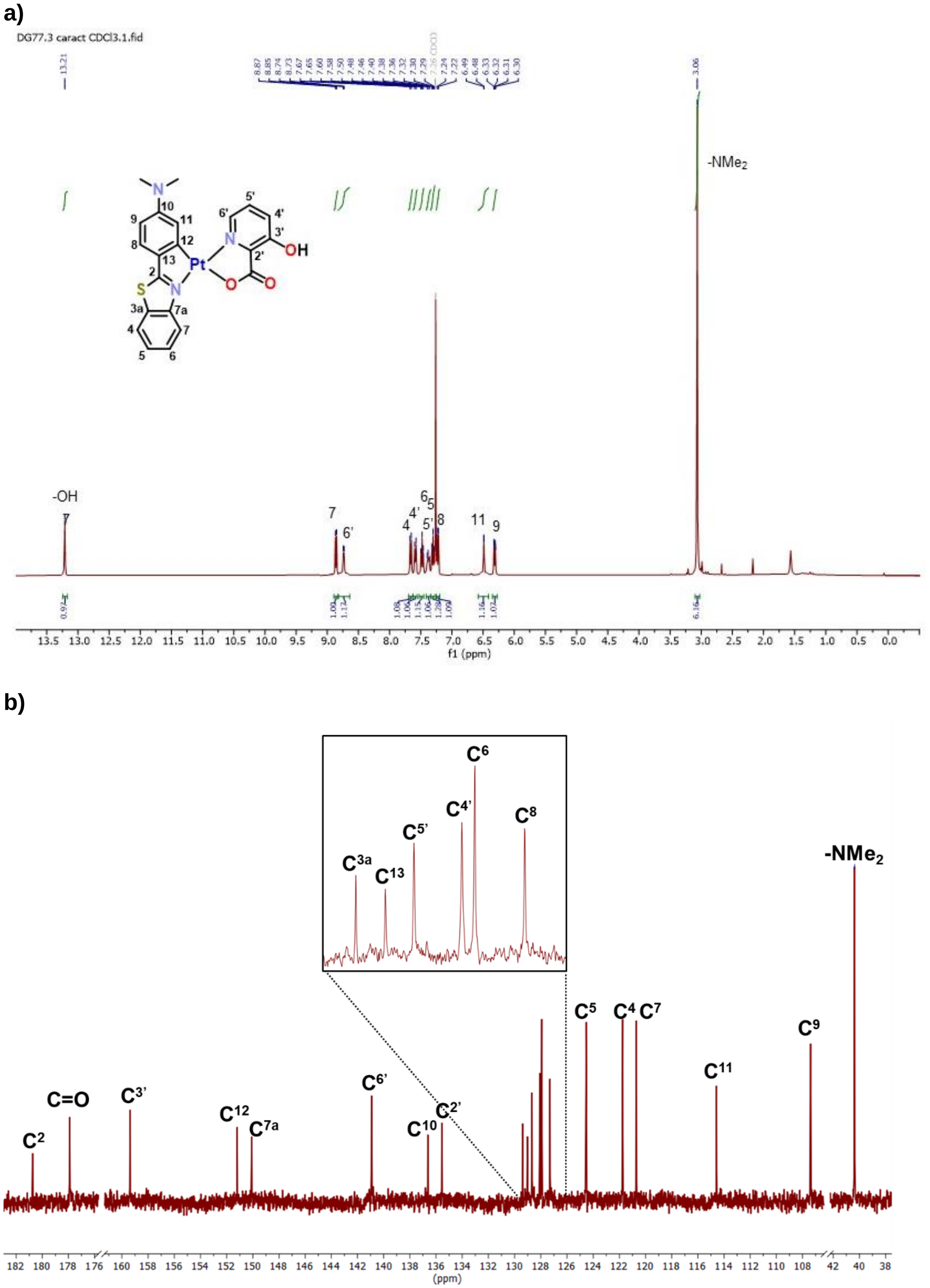

Figure S3. NMR spectra of **3** in $CDCl_3$ at 298 K (a) ^1H, (b) $^{13}C\{^1H\}$

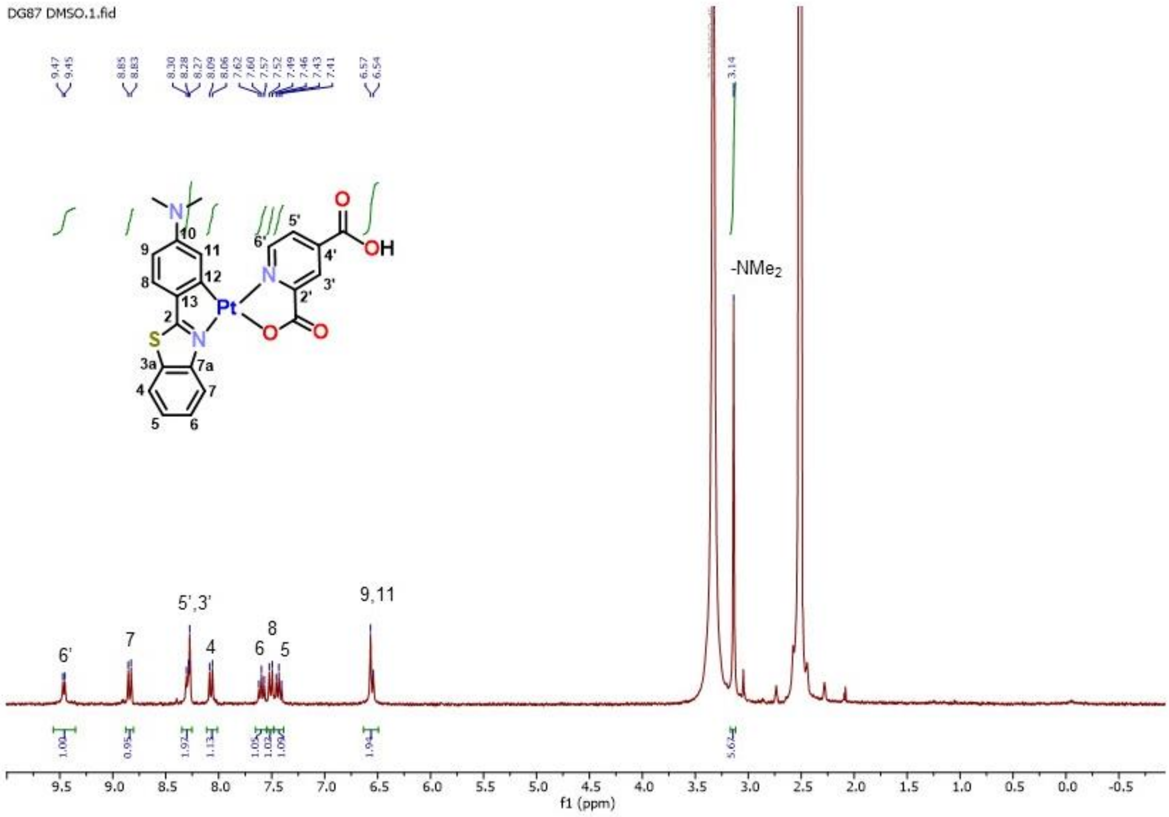

Figure S4. ^{1}H NMR spectra of **4** in DMSO-d^{6} at 298 K

2.- Crystal Structures

Table S1. X-ray Crystallographic data for **1** and **3·CHCl3**

	**1**	**3·CHCl3**
**Empirical formula**	$C_{21}H_{17}N_3O_2PtS$	$C_{21}H_{17}N_3O_3PtS$
**Molecular weight**	570.52	564.71
**T (K)**	120(2)	120(2)
**Wavelength (Å)**	0.71076	0.71076
**Crystal system**	Monoclinic	Monoclinic
**Space group**	$P2_1/n$	$P2_1/n$
**Crystal size (mm)**	0.372 x 0.127 x 0.033	0.252 x 0.073 x 0.060
**a (Å)**	11.6800(6)	6.9068(6)
**b (Å)**	7.6022(4)	22.472(2)
**c (Å)**	20.6723(9)	29.815(3)
**α (°)**	90.0	90.0
**β (°)**	97.483(2)	92.004(3)
**γ (°)**	90.0	90.0
**V (Å^3)**	1819.94(16)	4624.8(7)
**Z**	4	10
**Density (calculated) (Mg/cm^3)**	2.082	2.028
**Absorption coefficient (mm^{-1})**	7.848	6.536
**F(000)**	1096	2720
**θ range for data collection (°)**	2.858 to 27.954	2.270 to 27.923
**Index ranges**	-15<=h<=15, -10<=k<=10, -27<=l<=27	-9<=h<=9, -29<=k<=29, -39<=l<=39
**Reflections collected**	103007	297580
**Independent reflections**	4355 [R(int) = 0.0347]	11047 [R(int) = 0.0293]
**Data / restraints / parameters**	4355 / 0 / 306	11047 / 0 / 603
**Goodness-of-fit on F2**	1.093	1.075
**Final R indices [I>2σ(I)]**[a]	R1 = 0.0160, wR2 = 0.0396	R1 = 0.0192, wR2 = 0.0425
**R indices (all data)**[a]	R1 = 0.0170, wR2 = 0.0402	R1 = 0.0209, wR2 = 0.0434
**Largest diff. peak and hole (e Å^{-3}) (dmin/dmax)**	1.876 and -0.823	1.157 and -1.395

[a] $R1 = \Sigma(|F_o| - |F_c|)/\Sigma|F_o|$; $wR2 = [\Sigma w(F_o^2 - F_c^2)^2/\Sigma wF_o^2]^{1/2}$; goodness of fit = $\{\Sigma[w(F_o^2 - F_c^2)^2]/(N_{obs} - N_{param})\}^{1/2}$; $w = [\sigma^2(F_o) + (g_1P)^2 + g_2P]^{-1}$; $P = [\max(F_o^2;0 + 2F_c^2]/3$.

Table S2. Selected distances (Å) and angles (°) for complexes **1** and **3·$CHCl_3$**

**1**			
**Distances (Å)**		**Angles (°)**	
Pt(1)-N(1)	2.0185(19)	N(1)-Pt(1)-O(1)	97.90(8)
Pt(1)-N(3)	2.021(2)	N(3)-Pt(1)-O(1)	79.69(8)
Pt(1)-C(1)	1.999(2)	C(20)-N(3)-Pt(1)	114.03(17)
Pt(1)-O(1)	2.1036(18)	N(3)-C(20)-C(21)	116.0(2)
C(8)-C(9)	1.399(4)	O(1)-C(21)-C(20)	114.5(2)
		C(21)-O(1)-Pt(1)	114.41(16)
		N(1)-Pt(1)-C(1)	80.61(9)
		C(1)-Pt(1)-N(3)	102.46(9)
**3·$CHCl_3$**			
**Distances (Å)**		**Angles (°)**	
Pt(1)-N(1)	2.024(2)	N(1)-Pt(1)-O(1)	98.49(8)
Pt(1)-N(3)	2.031(2)	N(3)-Pt(1)-O(1)	79.46(8)
Pt(1)-C(1)	1.960(2)	C(20)-N(3)-Pt(1)	113.38(18)
Pt(1)-O(1)	2.1396(19)	N(3)-C(20)-C(21)	115.9(2)
C(8)-C(9)	1.425(3)	O(1)-C(21)-C(20)	117.2(2)
Pt(2)-N(4)	2.019(2)	C(21)-O(1)-Pt(1)	112.33(16)
Pt(2)-N(6)	2.027(2)	N(1)-Pt(1)-C(1)	80.92(9)
Pt(2)-C(22)	2.000(3)	C(1)-Pt(1)-N(3)	101.97(9)
Pt(2)-O(4)	2.1363(19)	N(4)-Pt(2)-O(4)	97.91(8)
C(27)-C(28)	1.420(3)	N(6)-Pt(2)-O(4)	79.43(8)
		C(41)-N(6)-Pt(2)	114.22(17)
		N(6)-C(41)-C(42)	115.8(2)
		O(4)-C(42)-C(41)	116.5(2)
		C(42)-O(4)-Pt(2)	112.33(16)
		N(4)-Pt(2)-C(22)	80.91(10)
		C(22)-Pt(2)-N(6)	102.70(10)

a)

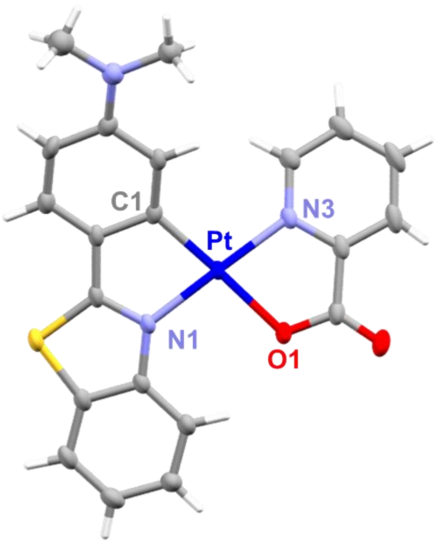

b)

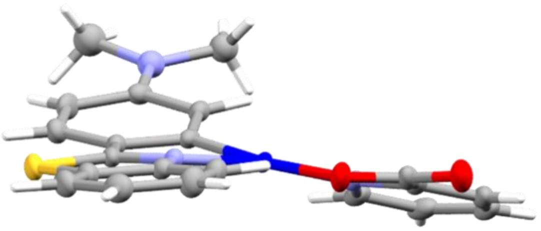

c)

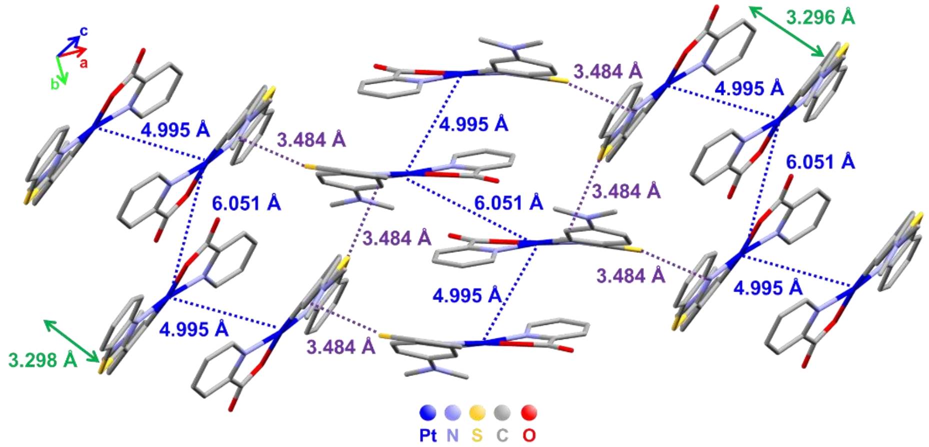

Figure S5. X-ray diffraction structure of **1**. a) and b) Two views of the molecular structure. c) Packing

a)

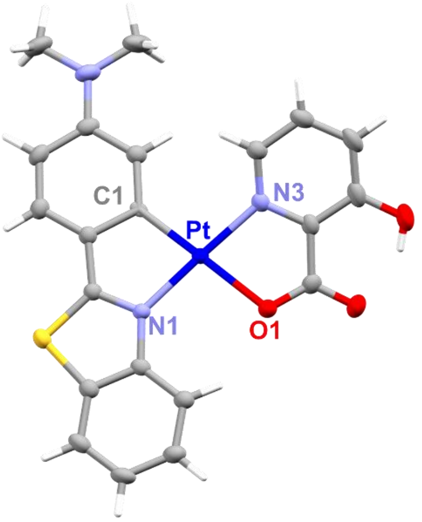

b)

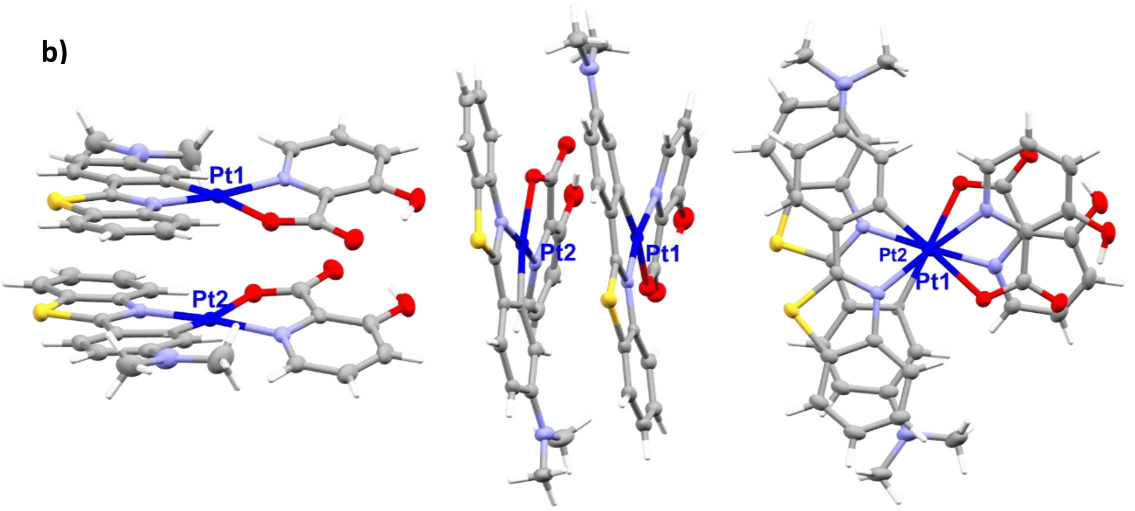

c)

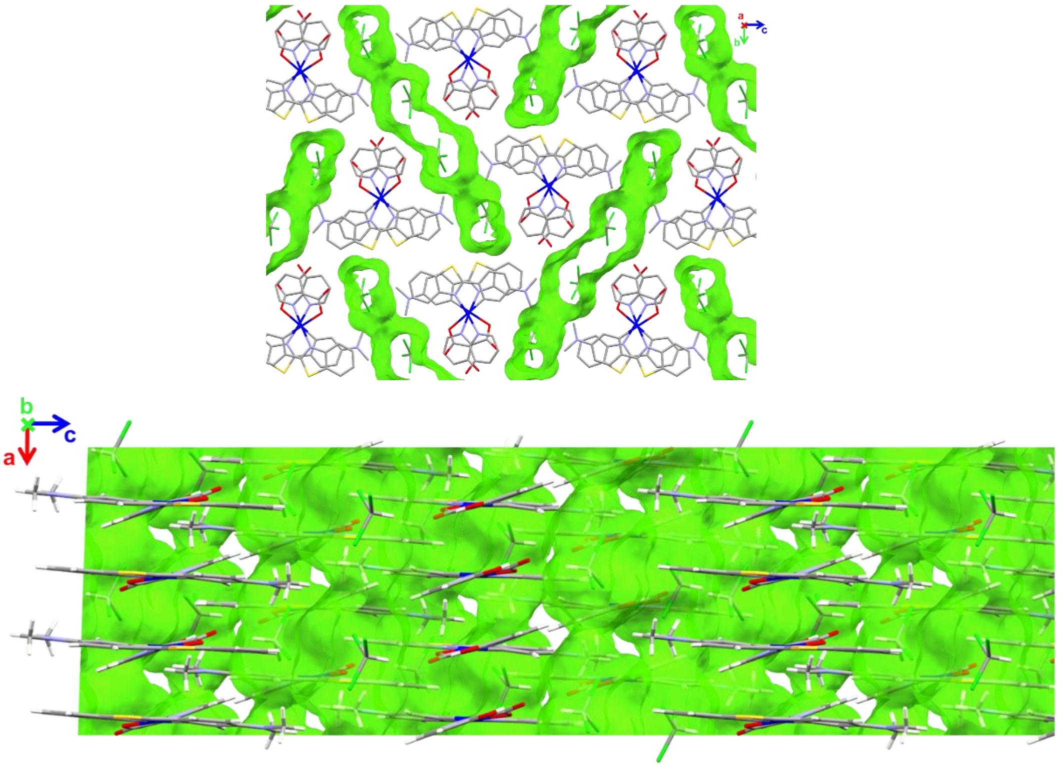

Figure S6. X-ray diffraction structure of **3·CHCl₃**. a) A view of the molecular structure. b) Several views of the dimers formed by the molecules **A** and **B**. c) Packing showing the localization of the $CHCl_3$ molecules.

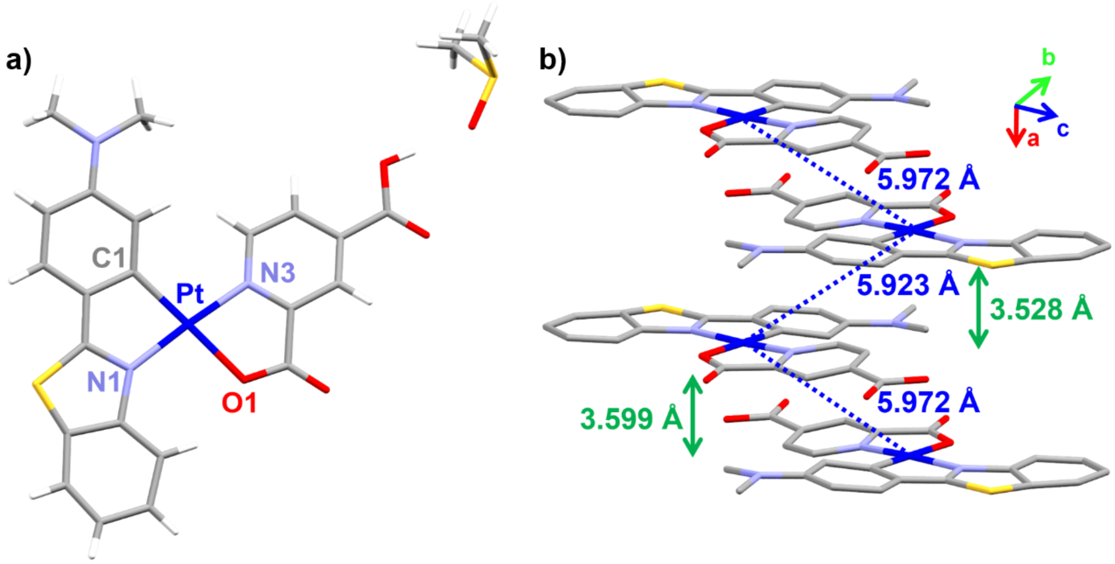

Figure S7. a) Molecular structure of **4·DMSO** showing the connectivity b) view of the molecular packing.

3.- Photophysical Properties and Theoretical calculations

**Table S3**. Absorption data for complexes **1** – **4** (solutions 5 × 10^{-5} M)		
**Compound**	**Media**	**λ_{abs}/nm (ε × 10^{-3}/$M^{-1}cm^{-1}$)**
**[Pt(Me_2N-pbt)(pic)] (1)**	CH_2Cl_2	247 (22.13), 293 (9.42), 315_{sh} (8.34), 358 (16.67), 376 (15.58), 445 (13.37), 460 (12.50)
	Solid	402, 430, 473, 505, 575_{sh}
**[Pt(Me_2N-pbt)(3-OH-pic)] (2)**	CH_2Cl_2	250 (27.22), 293 (10.21), 313_{sh} (9.62), 357 (22.00), 381_{sh} (16.01), 439 (13.75), 461 (11.60)
	Solid	390, 443, 472_{sh}, 574_{sh}
**[Pt(Me_2N-pbt)(3-NH_2-pic)] (3)**	CH_2Cl_2	243 (26.87), 295 (12.52), 310 (13.49), 322_{sh} (12.08), 359 (19.62), 385_{sh} (18.06), 446 (15.18), 464 (13.52)
	Solid	391, 430_{sh}, 457, 480_{sh}, 562_{sh}
**[Pt(Me_2N-pbt)(4-COOHpic)] (4)**	DMSO	295 (10.69), 315 (9.97), 360 (15.02), 384 (14.86), 453 (14.18)
	Solid	422, 494, 523, 640, 680, $725_{sh,}$ tail to 800

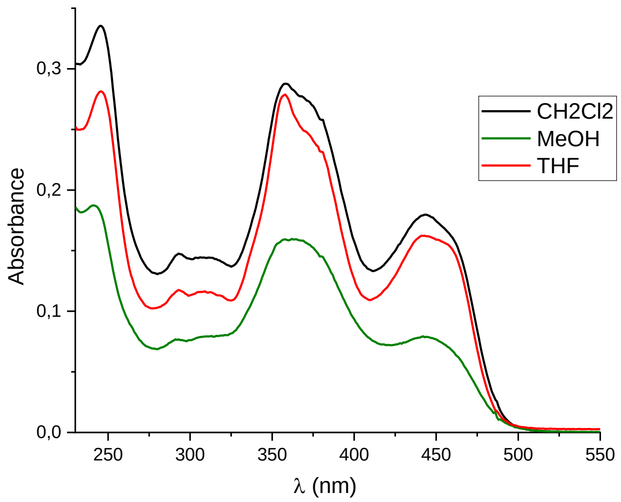

Figure S8. UV vis absorption spectra of **1** in different solvents (5 x 10^{-5} M)

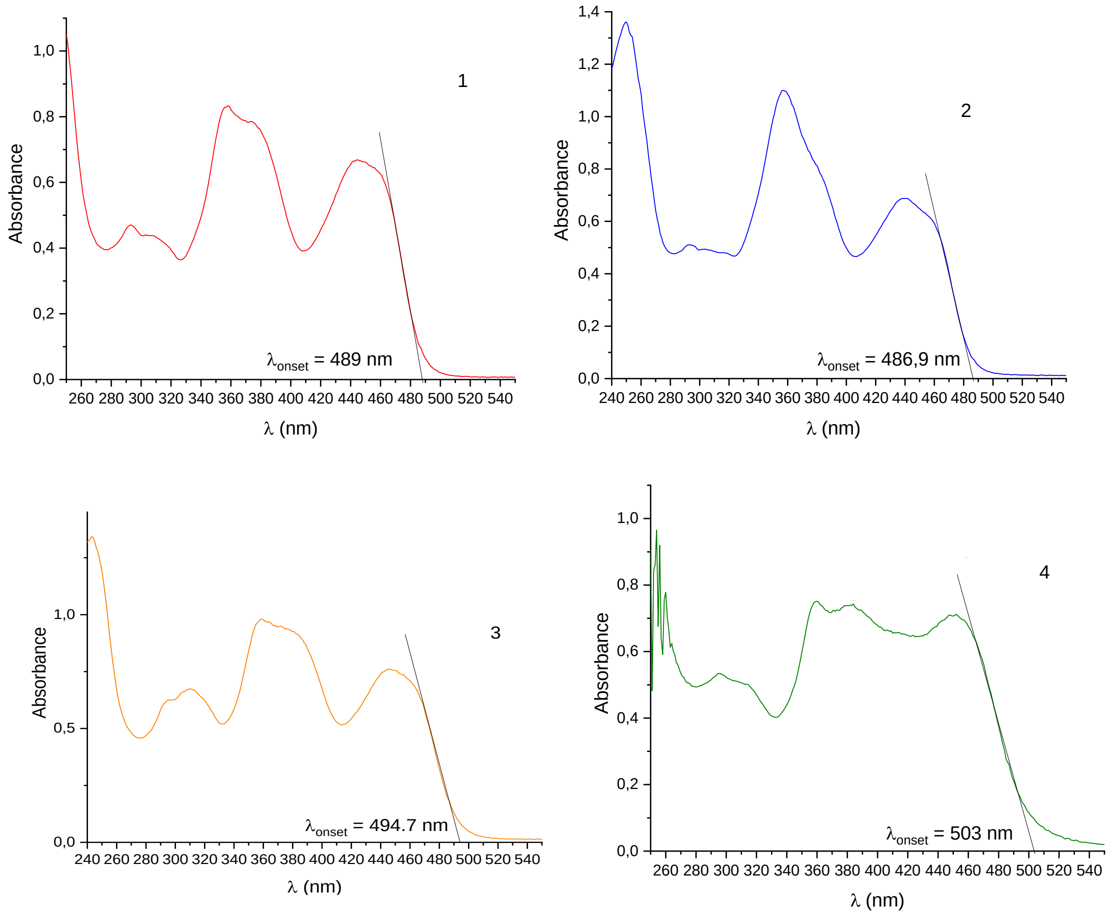

Figure S9. Onset determination of λ_{onset} from the UV-vis absorption spectra of **1-4**

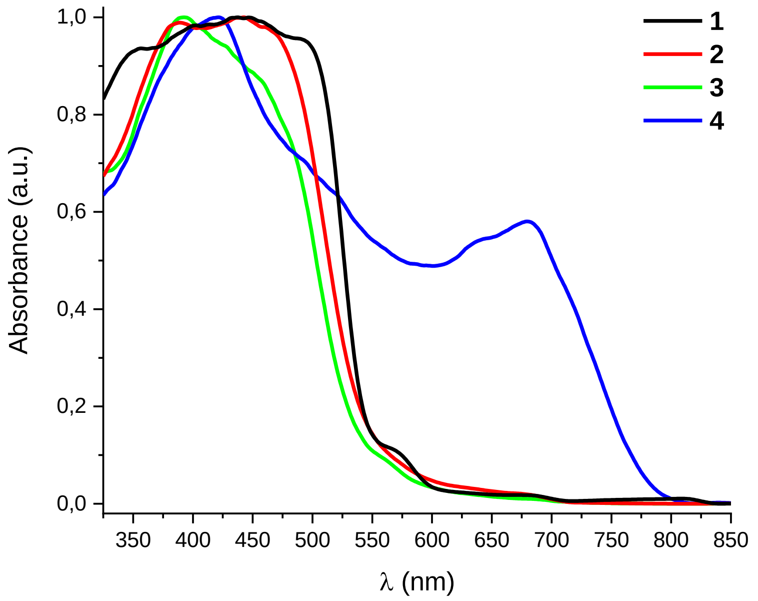

Figure S10. Normalized diffuse reflectance spectra in the solid state of **1-4**

Table S4. Selected parameter of DFT optimized geometries for ground state and triplet state in solution (PCM model; CH_2Cl_2 **1-3**, DMSO **4**).

**1**			
**Parameter**	**X-Ray**	**S_0**	**T_1**
Pt(1) - C(1)	1.999(2)	2.01512	2.00511
Pt(1) - N(1)	2.0185(19)	2.05076	2.02390
Pt(1) - N(3)	2.021(2)	2.05660	2.05419
Pt(1) - O(1)	2.1036(18)	2.16260	2.16246
C(1)-Pt(1)-N(1)	80.61(9)	80.10506	80.90593
N(1)-Pt(1)-O(1)	97.90(8)	99.15313	98.90093
O(1)-Pt(1)-N(3)	79.46(8)	78.40656	78.33843
N(3)-Pt(1)-C(1)	102.46(9)	102.94531	102.44383
**2**			
**Parameter**	**X-Ray**	**S_0**	**T_1**
Pt(1) - C(1)	-	2.01608	2.00651
Pt(1) - N(1)	-	2.05289	2.02552
Pt(1) - N(3)	-	2.05931	2.05881
Pt(1) - O(1)	-	2.15086	2.15176
C(1)-Pt(1)-N(1)	-	80.02525	80.81306
N(1)-Pt(1)-O(1)	-	99.28281	98.97418
O(1)-Pt(1)-N(3)	-	78.16691	78.09080
N(3)-Pt(1)-C(1)	-	103.13520	102.63849
**3**			
**Parameter**	**X-Ray**	**S_0**	**T_1**
Pt(1) - C(1)	1.960(2)	2.01141	2.00180
Pt(1) - N(1)	2.024(2)	2.05033	2.02379
Pt(1) - N(2)	2.031(2)	2.06389	2.06118
Pt(1) - O(1)	2.1396(19)	2.17824	2.17647
C(1)-Pt(1)-N(1)	80.92(9)	80.16920	80.91013
N(1)-Pt(1)-O(1)	98.49(8)	99.18649	98.82279
O(1)-Pt(1)-N(3)	79.46(8)	78.52947	78.45381
N(3)-Pt(1)-C(1)	101.97(9)	102.84846	102.39547
**4**			
**Parameter**	**X-Ray**	**S_0**	**T_1**
Pt(1) - C(1)	-	2.01335	2.00010
Pt(1) - N(1)	-	2.05040	2.05251
Pt(1) - N(2)	-	2.05450	2.02101
Pt(1) - O(1)	-	2.16923	2.13180
C(1)-Pt(1)-N(1)	-	80.09949	80.20560
N(1)-Pt(1)-O(1)	-	99.53282	98.49754
O(1)-Pt(1)-N(2)	-	78.37558	79.19423
N(2)-Pt(1)-C(1)	-	102.72405	102.71250

Table S5. DFT optimized geometries for ground state and triplet state (in CH_2Cl_2 **1-3**, DMSO **4**).

**1**	
**Singlet (S_0)**	**Triplet (T_1)**
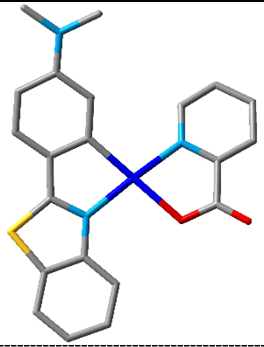	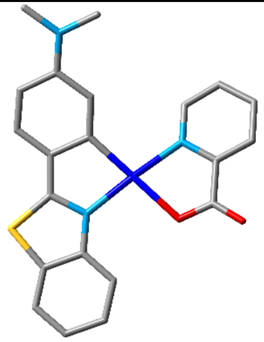
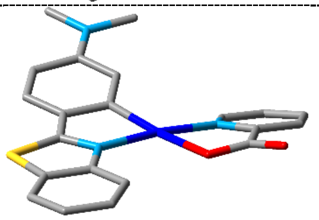	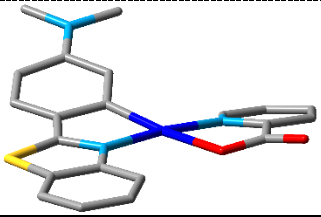
**2**	
**Singlet (S_0)**	**Triplet (T_1)**
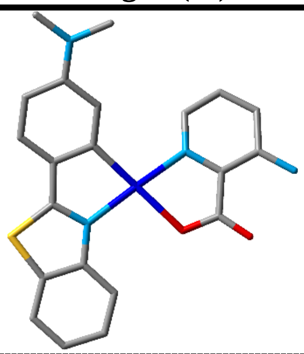	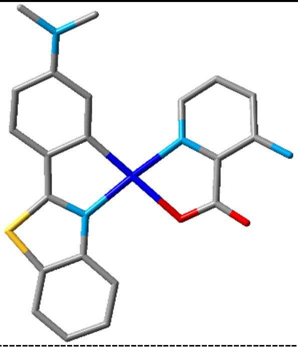
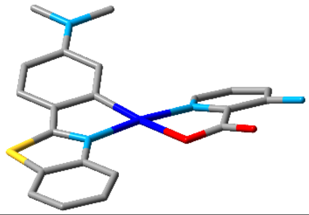	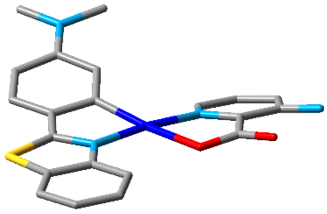
**3**	
**Singlet (S_0)**	**Triplet (T_1)**

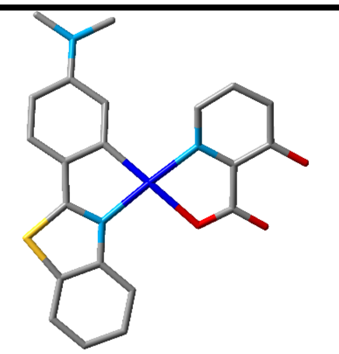	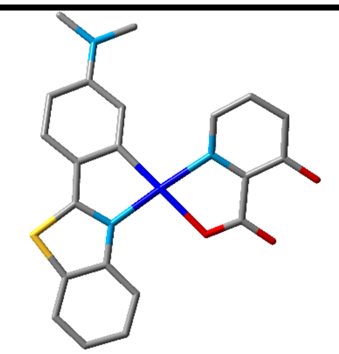
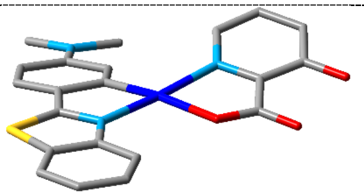	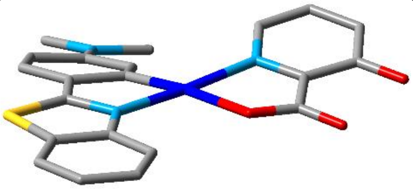
**4**	
**Singlet (S_0)**	**Triplet (T_1)**
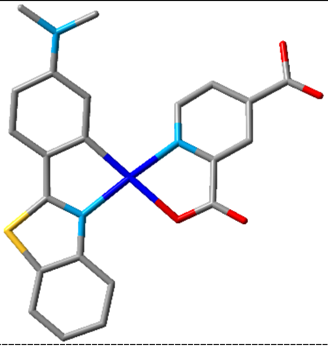	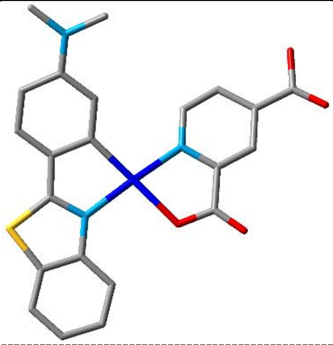
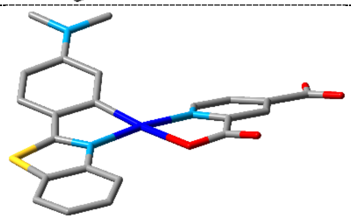	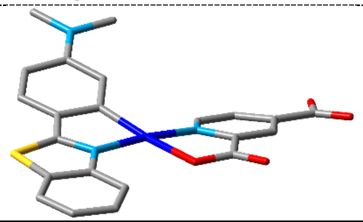

Table S6. Selected vertical excitations energies singlets (S_n) and first triplets (T_n) computed by TD-DFT/SCRF (CH_2Cl_2 **1-3**, DMSO **4**) with the orbitals involved.

	State	λ/nm	*f*	Transition (% Contribution)
**1**	T_1	563.9	-	HOMO→LUMO (55%), HOMO→L+1 (41%)
	T_2	442.2	-	HOMO→LUMO (40%), HOMO→L+1 (52%)
	T_3	409.4	-	H-1→LUMO (92%)
	S_1	458.3	0.1047	HOMO→LUMO (96%)
	S_2	398.2	0.2966	H-1→LUMO (21%), HOMO→L+1 (74%)
	S_3	380.7	0.2692	H-1→LUMO (74%), HOMO→L+1 (18%)
	S_4	359.0	0.0219	H-3→LUMO (39%), H-2→LUMO (58%)
	S_6	351.8	0.0049	HOMO→L+2 (96%)
	S_7	341.6	0.0866	H-1→L+1 (86%)
**2**	T_1	562.3	-	HOMO→LUMO (62%), HOMO→L+1 (35%)
	T_2	465.2	-	H-2→LUMO (67%), H-2→L+1 (22%)
	T_3	430.9	-	HOMO→LUMO (33%), HOMO→L+1 (57%)
	S_1	448.1	0.1463	HOMO→LUMO (95%)
	S_2	395.5	0.2874	H-1→LUMO (17%), HOMO→L+1 (78%)
	S_3	376.5	0.2405	H-1→LUMO (78%), HOMO→L+1 (14%)
	S_4	356.2	0.0110	H-4→LUMO (21%), H-3→LUMO (55%), H-2→LUMO (21%)
	S_6	341.7	0.0543	H-3→LUMO (10%), H-2→LUMO (18%), H-1→L+1 (64%)
	S_7	338.9	0.2911	H-3→LUMO (13%), H-2→LUMO (53%), H-1→L+1 (25%)
**3**	T_1	566.9	-	HOMO→LUMO (51%), HOMO→L+1 (46%)
	T_2	451.9	-	HOMO→LUMO (44%), HOMO→L+1 (49%)
	T_3	406.7	-	H-4→LUMO (11%), H-1→LUMO (70%)
	S_1	467.9	0.0819	HOMO→LUMO (97%)
	S_2	399.6	0.3629	H-1→LUMO (17%), HOMO→L+1 (78%)
	S_3	379.9	0.2278	H-1→LUMO (79%), HOMO→L+1 (15%)
	S_4	363.5	0.0189	H-3→LUMO (48%), H-2→LUMO (49%)
	S_6	340.0	0.1145	H-3→LUMO (19%), H-2→LUMO (19%), H-1→L+1 (55%)
	S_7	335.1	0.2283	H-3→LUMO (29%), H-2→LUMO (26%), H-1→L+1 (35%)
**4**	T_1	617.1	-	HOMO→LUMO (80%), HOMO→L+1 (18%)
	T_2	528.1	-	HOMO→LUMO (18%), HOMO→L+1 (78%)
	T_3	469.3	-	H-1→LUMO (95%)

	S_1	581.9	0.0170	HOMO→LUMO (99%)
	S_2	453.6	0.0144	H-1→LUMO (97%)
	S_3	416.4	0.0212	H-3→LUMO (44%), H-2→LUMO (50%)
	S_4	403.7	0.5488	HOMO→L+1 (84%)
	S_6	378.4	0.1612	H-3→LUMO (27%), H-2→LUMO (28%), HOMO→L+2 (38%)
	S_7	375.0	0.1177	H-3→LUMO (17%), H-2→LUMO (18%), HOMO→L+2 (61%)

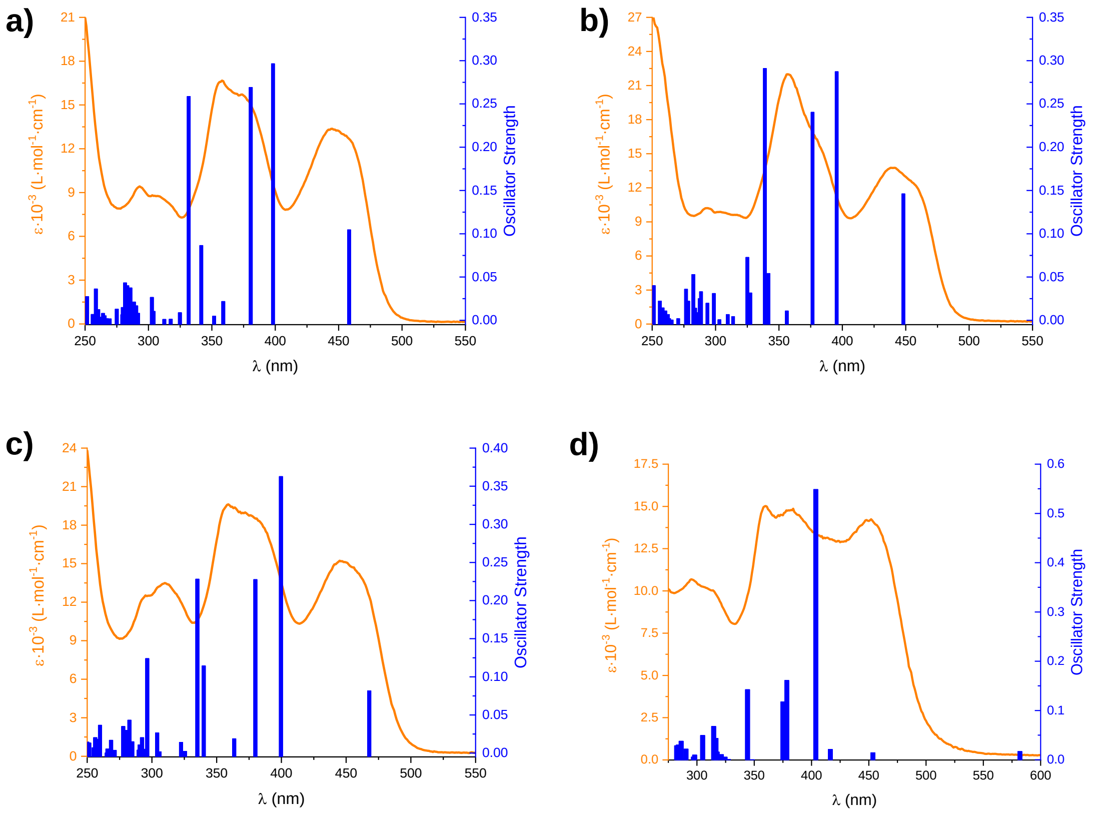

Figure S11. Calculated stick absorption spectra (blue bars) of a) **1**, b) **2**, c) **3** and d) **4** in solution compared with the experimental spectra (orange line).

Table S7. Composition (%) of Frontier MOs in terms of ligands and metals in the ground state in solution (CH_2Cl_2 **1-3**, DMSO **4**).

**1**				
**Orbital**	**Energy (eV)**	**Pt**	**Me_2N-pbt**	**pic**
**LUMO+5**	-0.14	20	77	3
**LUMO+4**	-0.30	7	91	2
**LUMO+3**	-0.36	42	44	14
**LUMO+2**	-1.18	2	2	96
**LUMO+1**	-1.61	3	69	28
**LUMO**	-2.00	5	28	67
**HOMO**	-5.21	4	95	1
**HOMO-1**	-5.97	44	44	13
**HOMO-2**	-6.28	58	37	5
**HOMO-3**	-6.37	69	28	4
**HOMO-4**	-6.80	3	96	1

Orbital	Energy (eV)	Pt	Me_2N-pbt	
**HOMO-5**	-6.89	9	10	80
		**2**		
**Orbital**	**Energy (eV)**	**Pt**	**Me_2N-pbt**	**NH_2-pic**
**LUMO+5**	-0.11	20	77	3
**LUMO+4**	-0.25	31	43	26
**LUMO+3**	-0.30	9	88	3
**LUMO+2**	-0.53	10	8	83
**LUMO+1**	-1.56	3	60	37
**LUMO**	-1.91	5	37	58
**HOMO**	-5.17	4	95	1
**HOMO-1**	-5.92	42	42	16
**HOMO-2**	-6.06	10	5	85
**HOMO-3**	-6.24	65	31	5
**HOMO-4**	-6.36	56	30	14
**HOMO-5**	-6.78	3	96	1
		**3**		
**Orbital**	**Energy (eV)**	**Pt**	**Me_2N-pbt**	**OH-pic**
**LUMO+5**	-0.18	19	78	3
**LUMO+4**	-0.33	5	94	1
**LUMO+3**	-0.43	42	35	23
**LUMO+2**	-0.77	7	6	88
**LUMO+1**	-1.66	3	75	22
**LUMO**	-2.08	5	22	73
**HOMO**	-5.23	4	96	1
**HOMO-1**	-6.07	43	48	9
**HOMO-2**	-6.32	53	42	5
**HOMO-3**	-6.40	72	20	8
**HOMO-4**	-6.79	4	34	63
**HOMO-5**	-6.84	3	75	22
		**4**		
**Orbital**	**Energy (eV)**	**Pt**	**Me_2N-pbt**	**4-COOH-pic**
**LUMO+5**	-0.23	19	73	8
**LUMO+4**	-0.37	6	92	1
**LUMO+3**	-0.51	46	39	15
**LUMO+2**	-1.48	1	1	98
**LUMO+1**	-1.76	4	91	4
**LUMO**	-2.65	4	4	92
**HOMO**	-5.25	3	96	1
**HOMO-1**	-6.09	43	46	11
**HOMO-2**	-6.38	47	48	5
**HOMO-3**	-6.50	76	21	3
**HOMO-4**	-6.85	3	97	1
**HOMO-5**	-7.06	20	23	57

a)

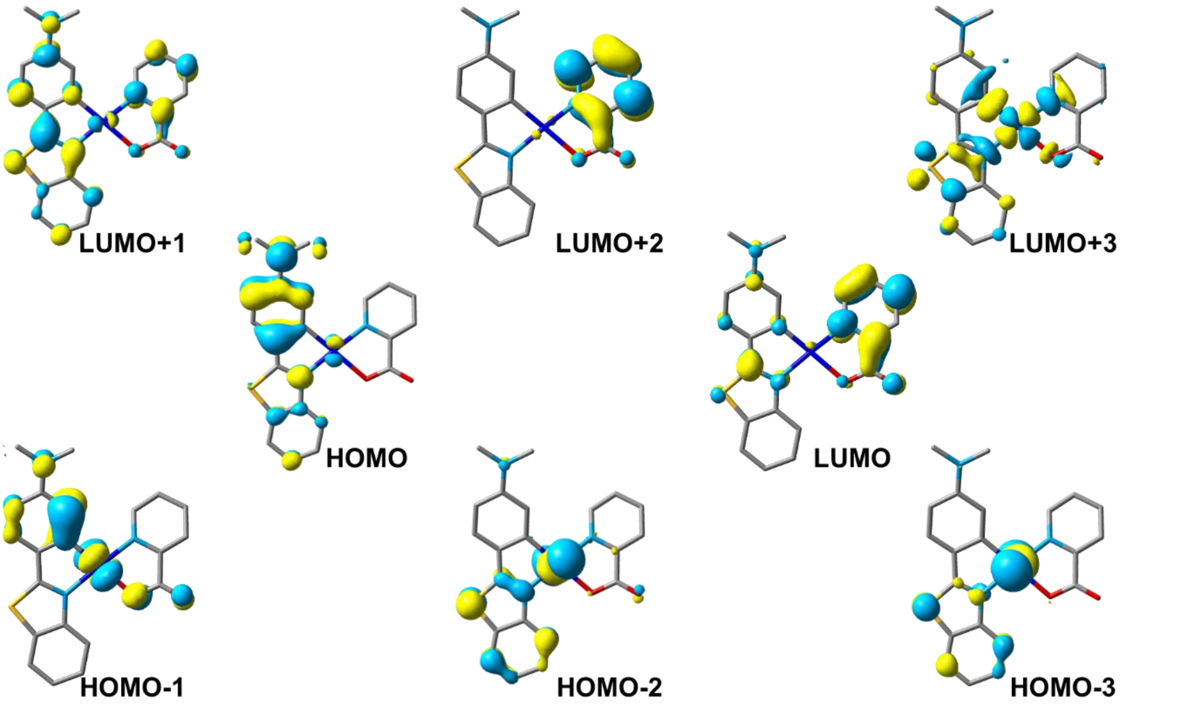

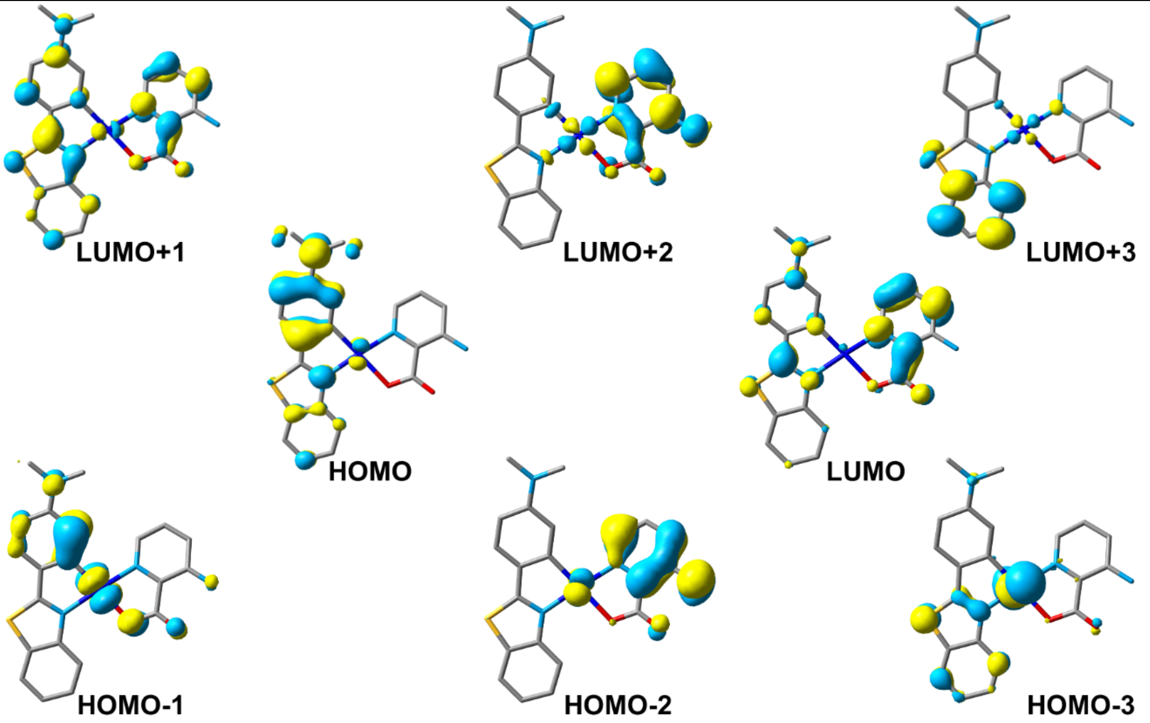
LUMO+1
LUMO+2
LUMO+3
HOMO
LUMO
HOMO-1
HOMO-2
HOMO-3

b)

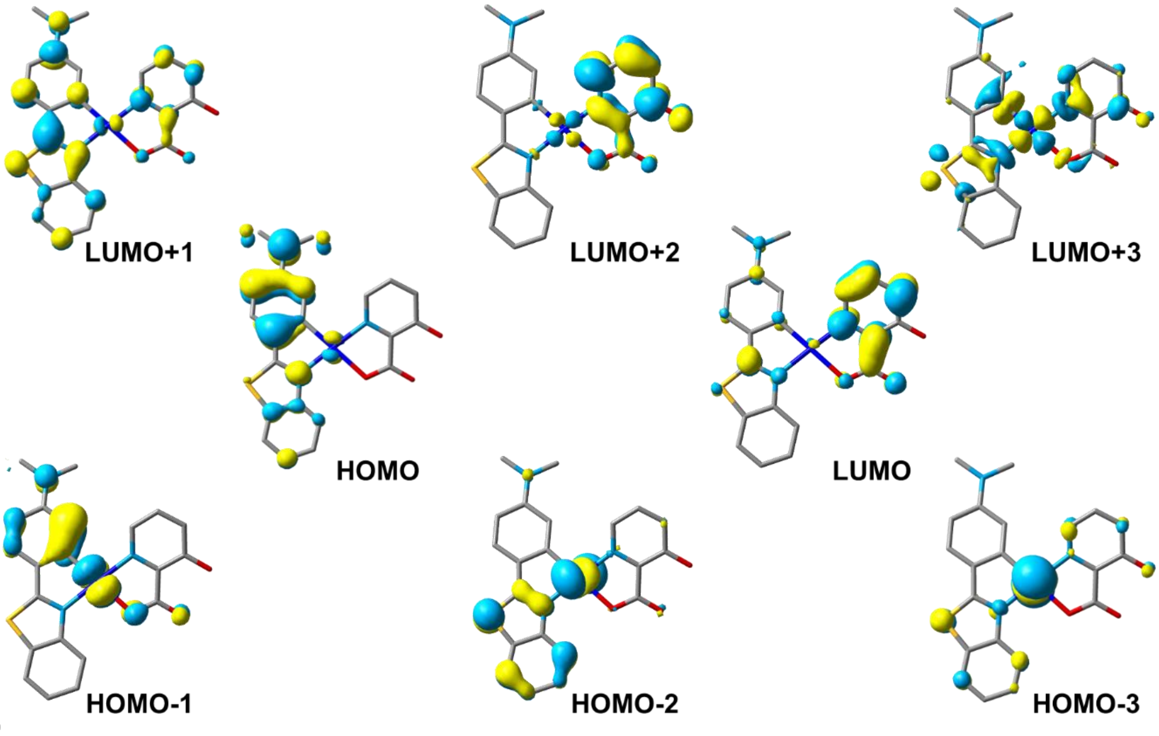
LUMO+1
LUMO+2
LUMO+3
HOMO
LUMO
HOMO-1
HOMO-2
HOMO-3

c)

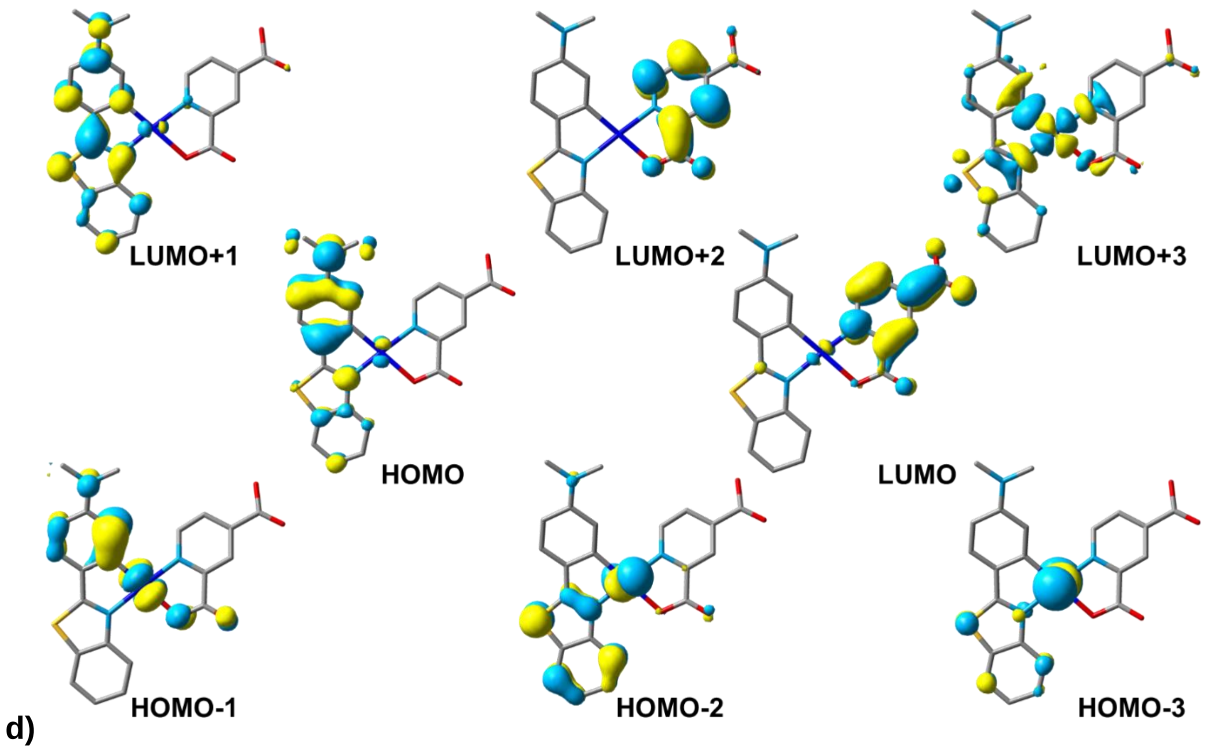

Figure S12. Selected frontier Molecular Orbitals for a) **1**, b) **2**, c) **3** and d) **4** in the ground state in solution.

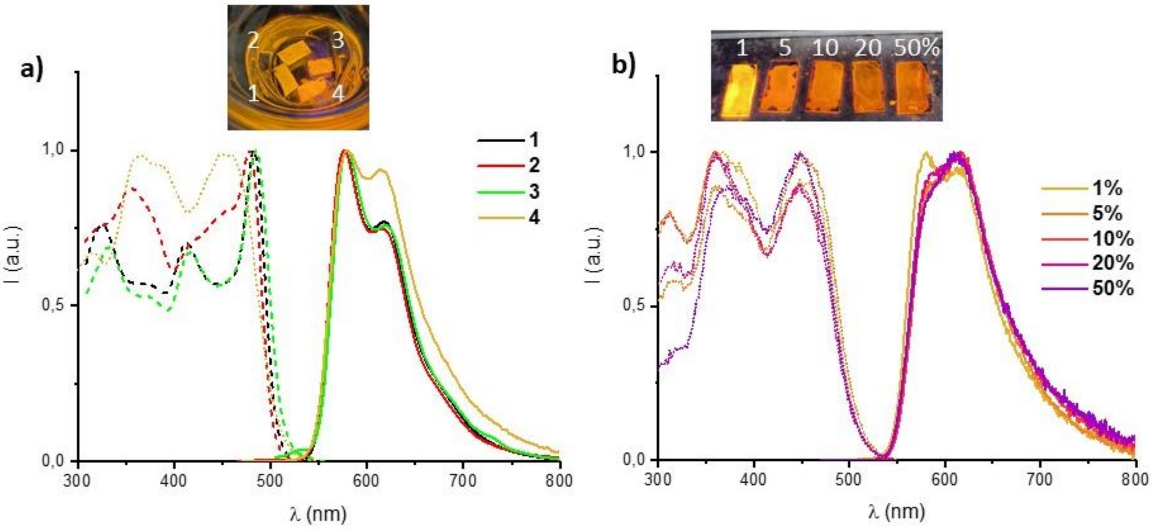

Figure S13. a) Normalized excitation and emission spectra of the complexes **1** – **4** in PS film (1% *wt*) (λ_{ex} = 450 nm) and photographs under deoxygenated conditions, b) Normalized excitation and emission spectra of the complex **3** in PS films at different concentrations (λ_{ex} = 450 nm) and photographs under deoxygenated conditions.

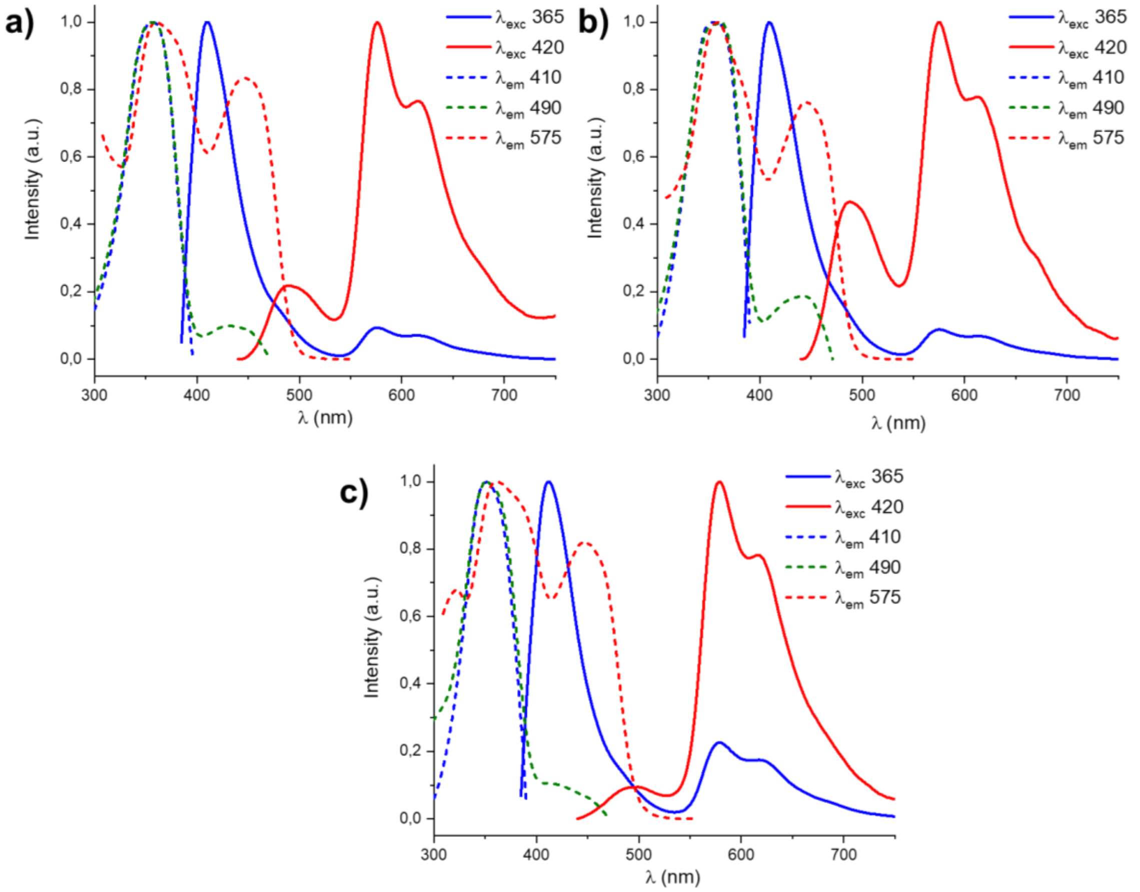

Figure S14. Normalized excitation and emission spectra of complexes a) **1**, b) **2** and c) **3** in aerated CH_2Cl_2 solution (5×10^{-5} M).

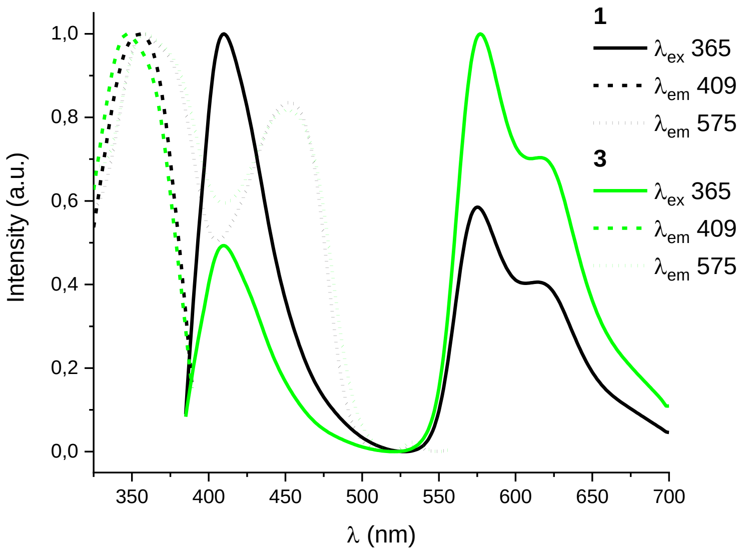

Figure S15. Normalized excitation and emission spectra of complexes a) **1** and b) **3** in deoxygenated CH_2Cl_2 solution (5×10^{-5} M).

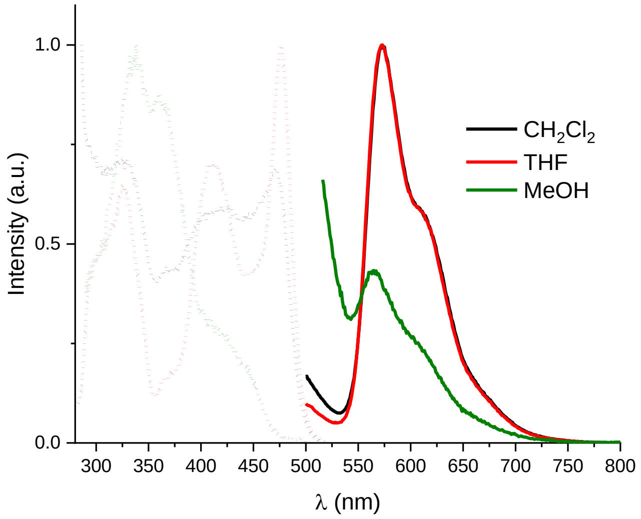

Figure S16. Normalized excitation and emission spectra of complex **1** in deoxygenated CH_2Cl_2, THF or MeOH (5×10^{-5} M) solution at 298 K (λ_{exc} 450 nm; λ_{em} 570 nm).

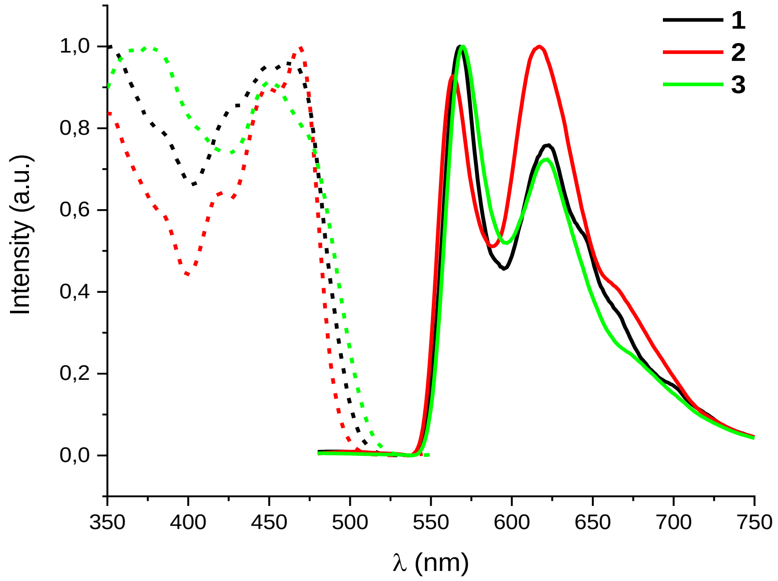

Figure S17. Normalized excitation and emission spectra of complexes **1** – **3** in 5×10^{-5} M CH_2Cl_2 solution at 77 K (λ_{ex} 460 nm).

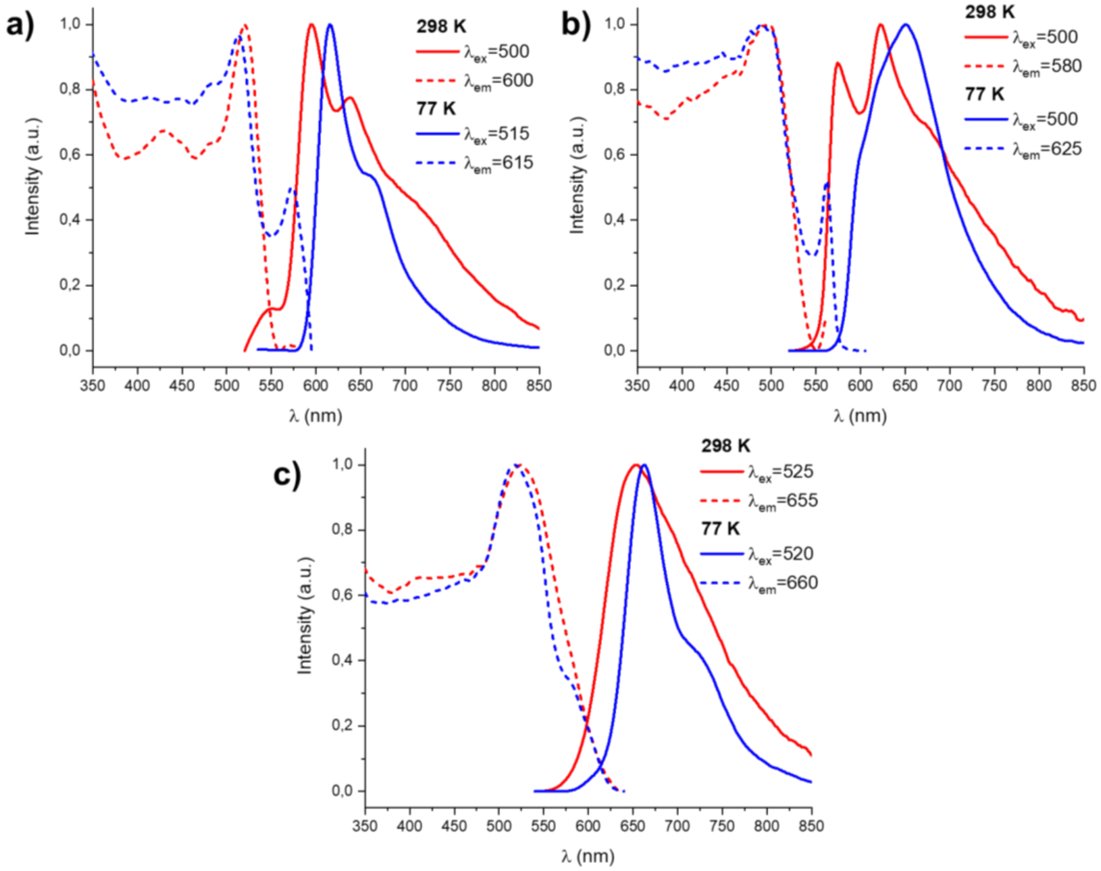

Figure S18. Normalized excitation and emission spectra of complexes a) **1**, b) **3** and c) **2** in solid state at 298 and 77 K.

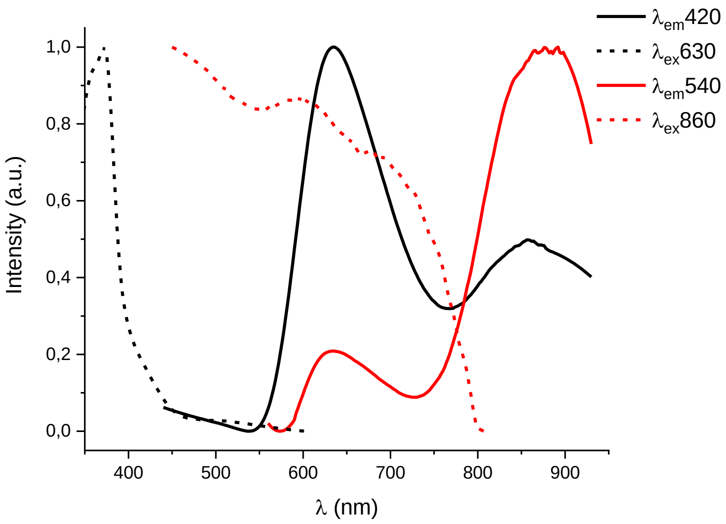

Figure S19. Normalized excitation and emission spectra of complexes **4** in solid state at 298 K.

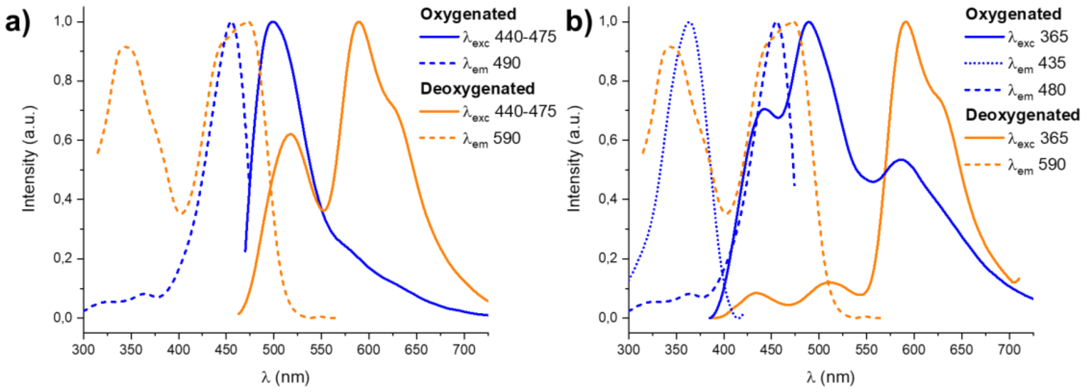

Figure S20. Excitation and emission spectra of complex **4** in oxygenated and deoxygenated DMSO solution (5×10^{-4} M) at 298 K upon excitation at a) 440-475 nm and b) 365 nm.

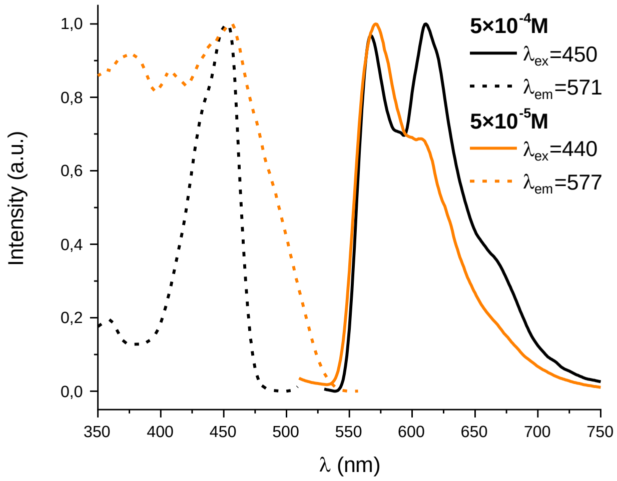

Figure S21. Normalized excitation and emission spectra of complexes **4** in deoxygenated DMSO (5×10^{-4} and 5×10^{-5} M) solution at 77 K.

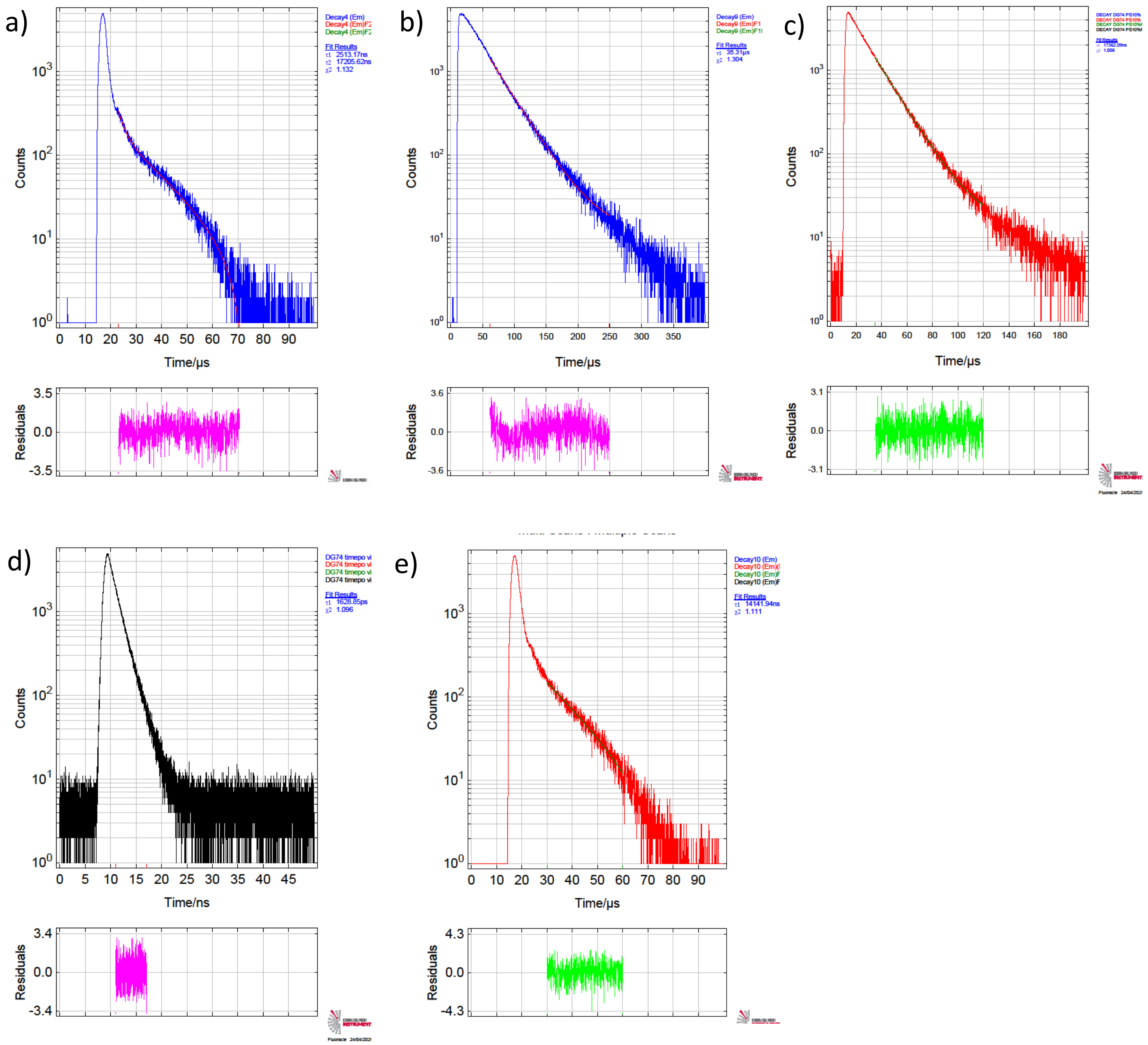

Figure S22-1. Lifetime decay curves and fits for complex **1** (a) Solid, 298 K, λ_{em} 595 nm (b) Solid, 77 K, λ_{em} 615 nm (c) PS 1%, λ_{em} 572 nm (d) CH_2Cl_2 5 x 10^{-5} M λ_{em} 409 nm (e) CH_2Cl_2 5 x 10^{-5} M λ_{em} 575 nm

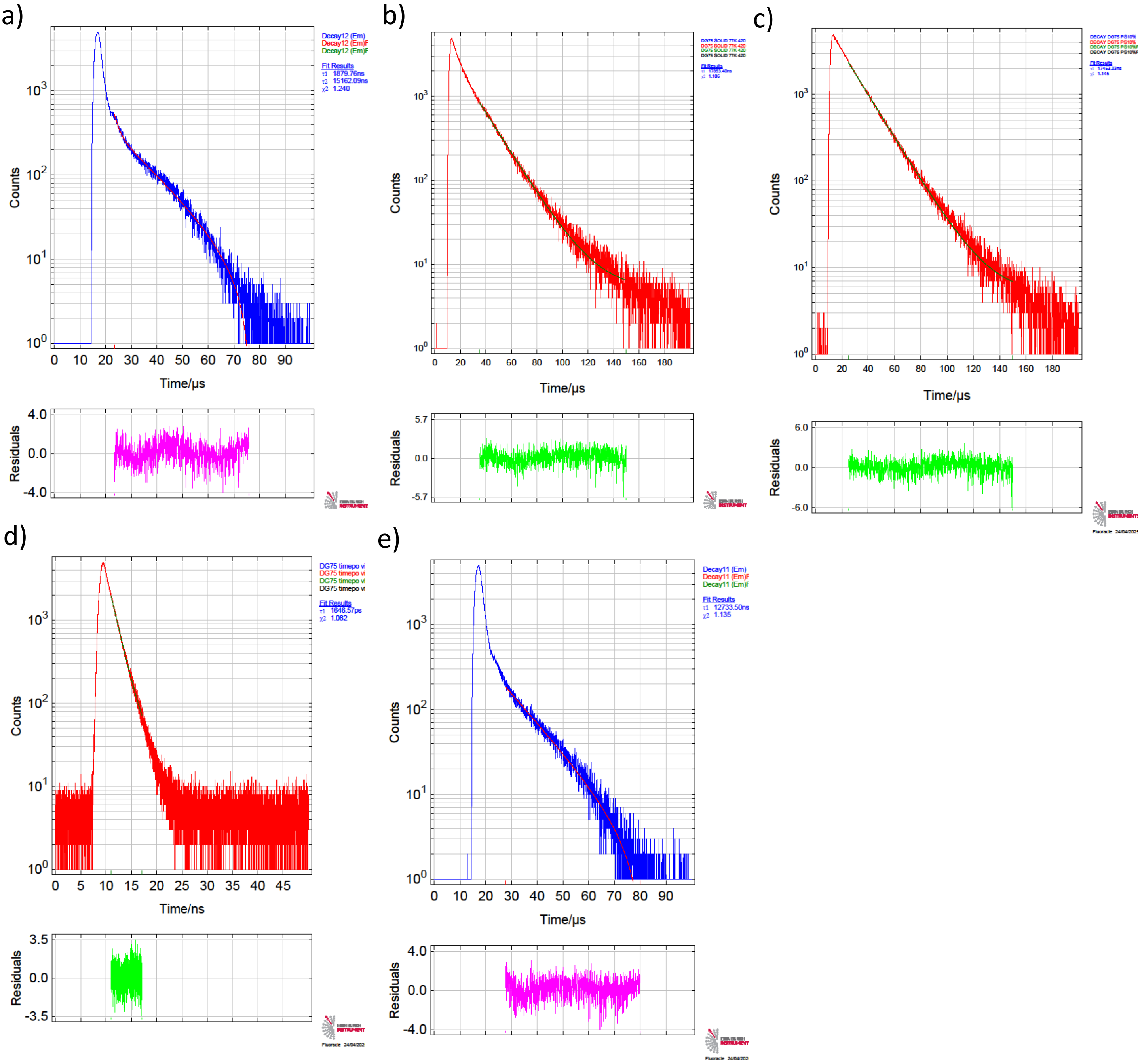

Figure S22-2. Lifetime decay curves and fits for complex **2** (a) Solid, 298 K, λ_{em} 655 nm (b) Solid, 77 K, λ_{em} 660 nm (c) PS 1%, λ_{em} 568 nm (d) CH_2Cl_2 5 x 10^{-5} M λ_{em} 409 nm (e) CH_2Cl_2 5 x 10^{-5} M λ_{em} 574 nm.

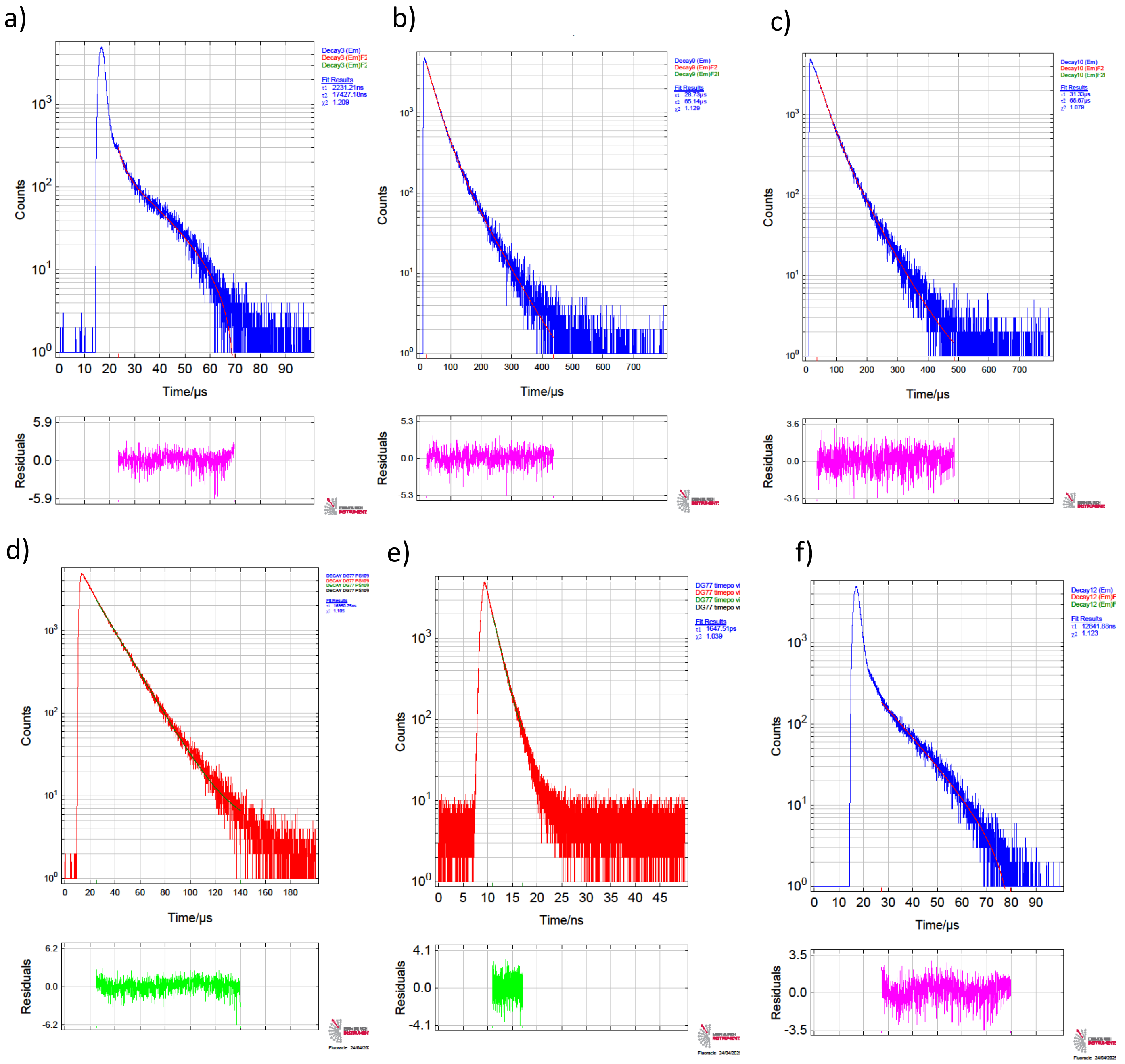

Figure S22-3. Lifetime decay curves and fits for complex **3** (a) Solid, 298 K, λ_{em} 576 nm (b) Solid, 77 K, λ_{em} 600 nm (c) Solid, 77 K, λ_{em} 625 nm (d) PS 1%, λ_{em} 580 nm (e) CH_2Cl_2 5 x 10^{-5} M λ_{em} 409 nm (f) CH_2Cl_2 5 x 10^{-5} M λ_{em} 577 nm

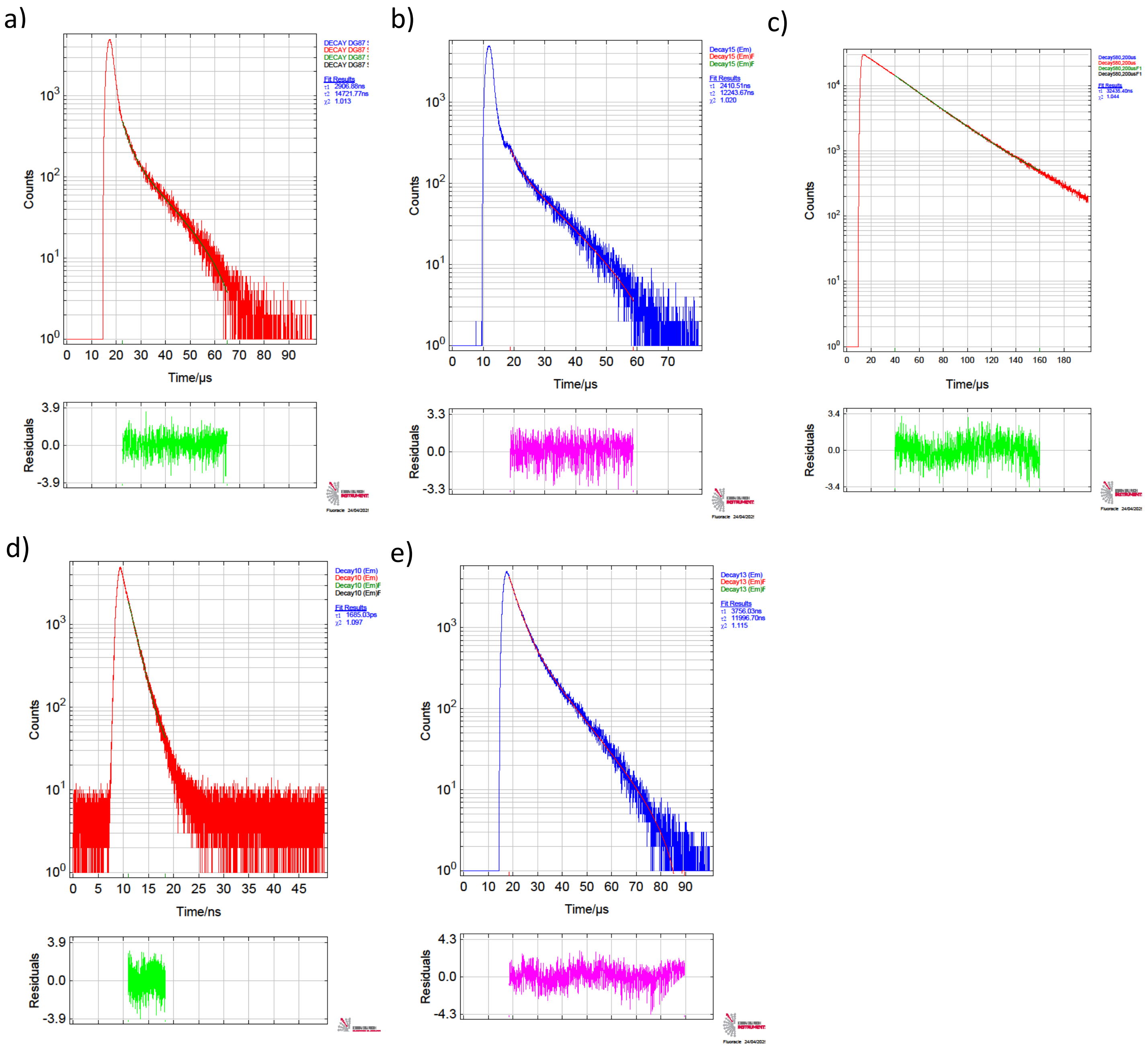

Figure S22-4. Lifetime decay curves and fits for complex **4** (a) Solid, 298 K, λ_{em} 630 nm (b) Solid 298 K, λ_{em} 880 nm (c) PS 1%, λ_{em} 580 nm (d) DMSO 5 x 10^{-5} M λ_{em} 588 nm (e) DMSO 5 x 10^{-5} M λ_{em} 588 nm.

Table S8. Plots and composition (%) of the frontier MOs and the spin density of the first triplet state in solution

**1**	
**HSOMO**	**LSOMO**
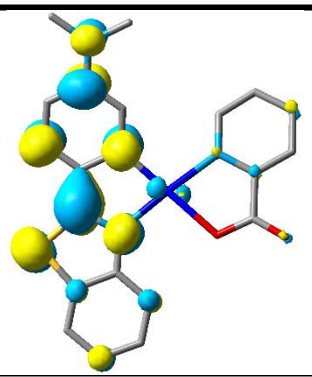	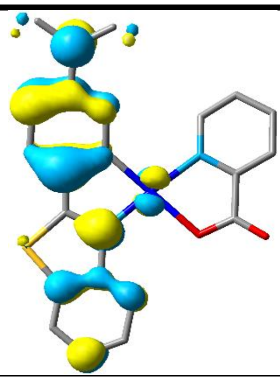
- 3.19 eV<br>Me_2N-pbt 90%, pic 6%, Pt 5%	- 4.00 eV<br>Me_2N-pbt 94%, pic 1%, Pt 5%

**2**	
**HSOMO**	**LSOMO**
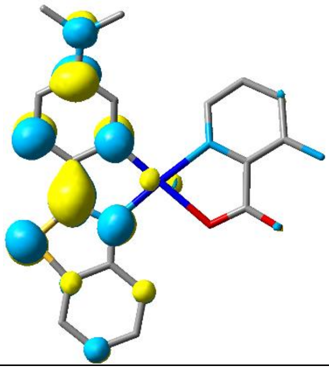	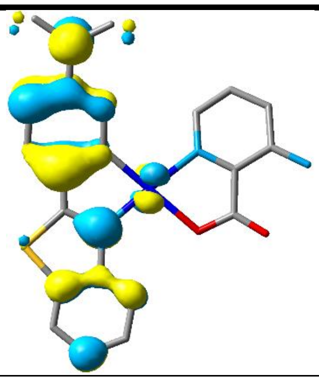
-3.16 eV<br>Me_2N-pbt 91%, NH_2-pic 5%, Pt 4%	-3.96 eV<br>Me_2N-pbt 94%, NH_2-pic 1%, Pt 5%

**3**	
**HSOMO**	**LSOMO**
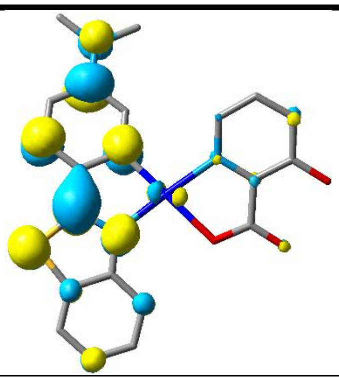	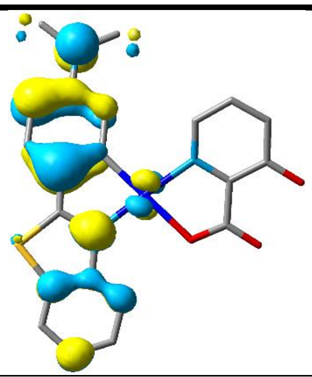
-3.22 eV<br>Me_2N-pbt 89%, OH-pic 7%, Pt 5%	-4.03 eV<br>Me_2N-pbt 94%, OH-pic 1%, Pt 5%

4	
**HSOMO**	**LSOMO**
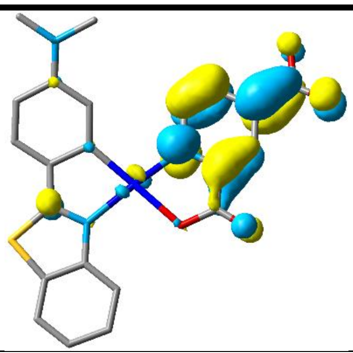	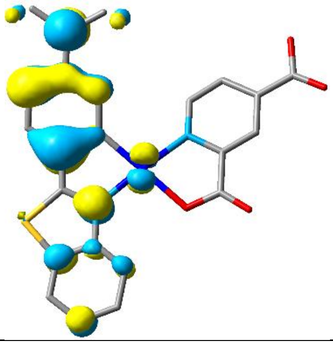
-3.44 eV<br>Me_2N-pbt 11%, 4-COOH-pic 85%, Pt 4%	-4.58 eV<br>Me_2N-pbt 93%, 4-COOH-pic 1%, Pt 6%

Table S9. Calculated emission energies (λ_{em} / nm) in solution.

Complex	Emission energy calculated (nm)
**1**	635
**2**	635
**3**	637
**4**	759

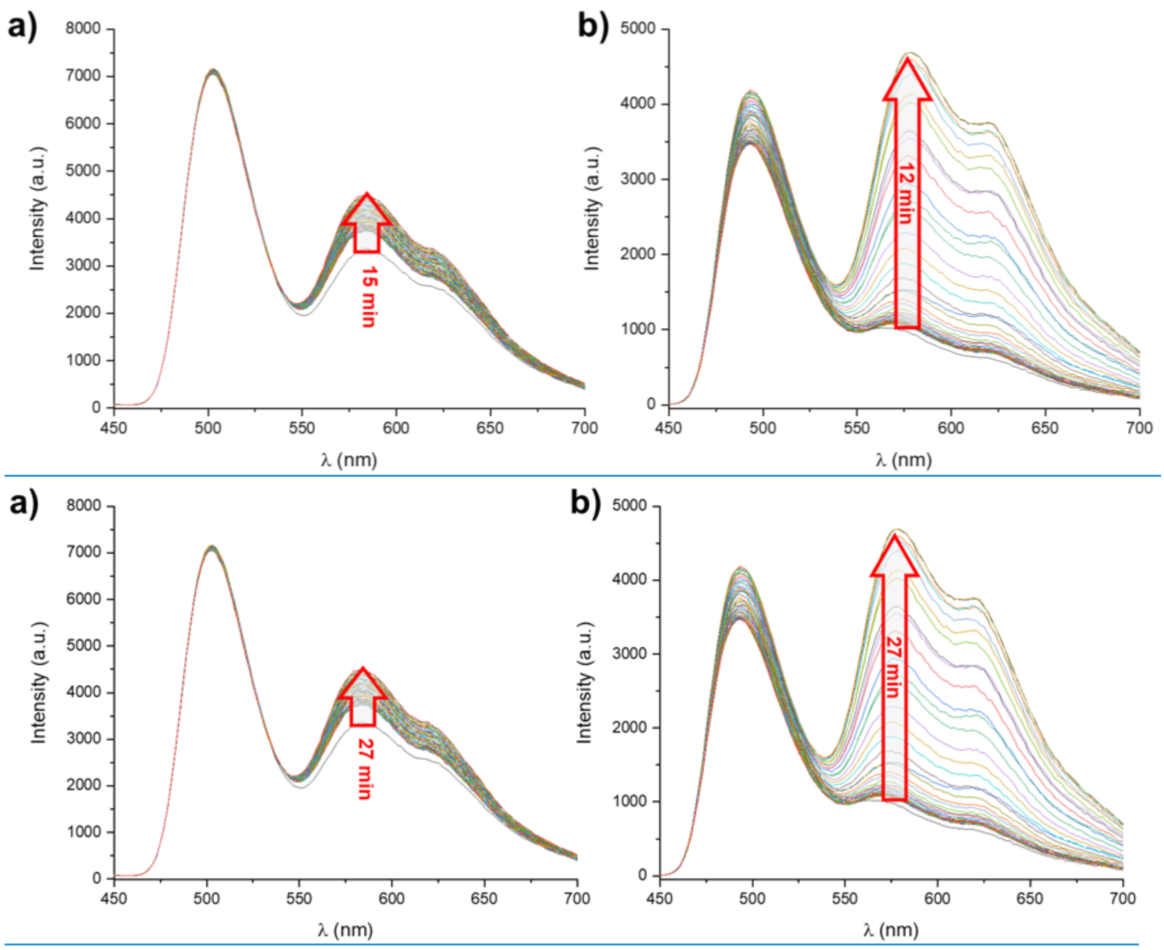

Figure S23. Enhancement of the phosphorescent emission of (a) **1** and (b) **3** in an aerated DMSO solution upon excitation at 365 nm and emission spectra in DMSO 5 × 10^{-4} M solution in presence of O_2 with different irradiation times.

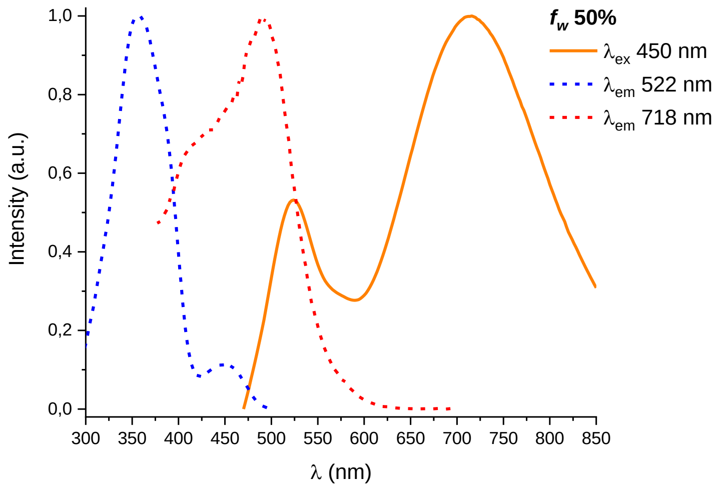

Figure S24. Excitation and emission spectra of a DMSO-H_2O mixture (f_w 50%) of **3** (2×10^{-4} M) (λ_{exc} 450 nm).

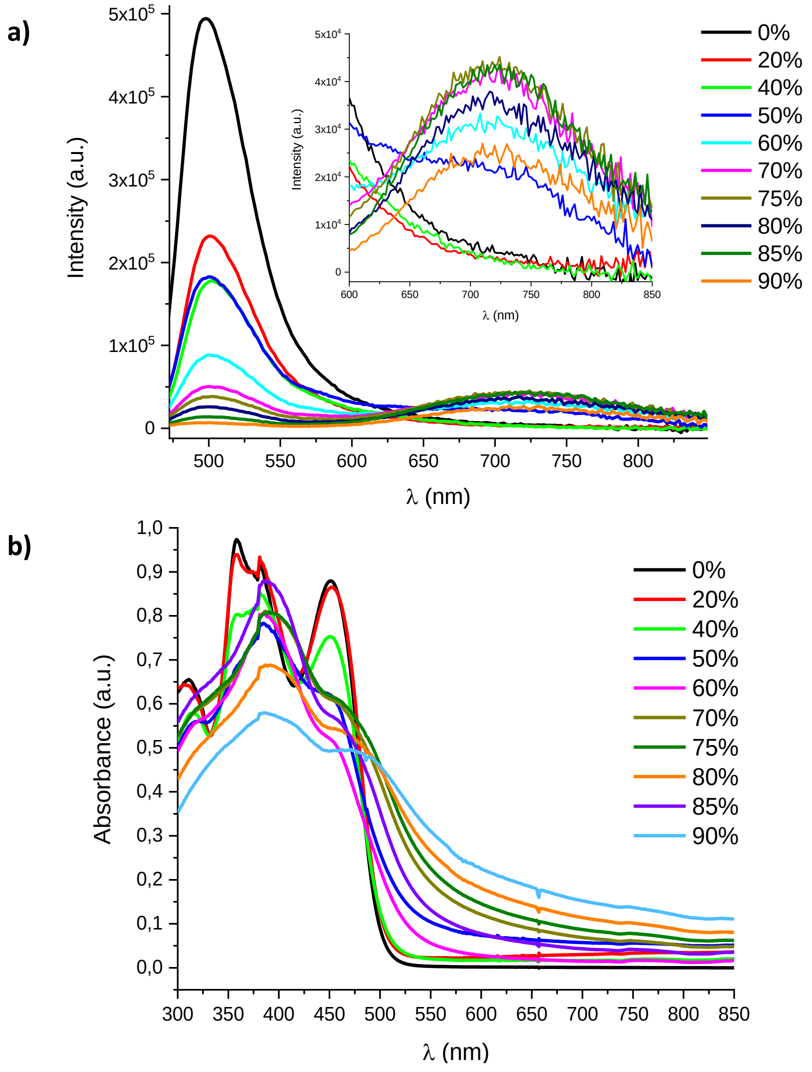

Figure S25. DMSO-H_2O mixtures of **3** (total concentration 5×10^{-5} M) with different H_2O fractions (0-90% at 298 K) a) Emission spectra (λ_{exc} 450 nm), b) UV-vis absorption.

4.- Biological Properties

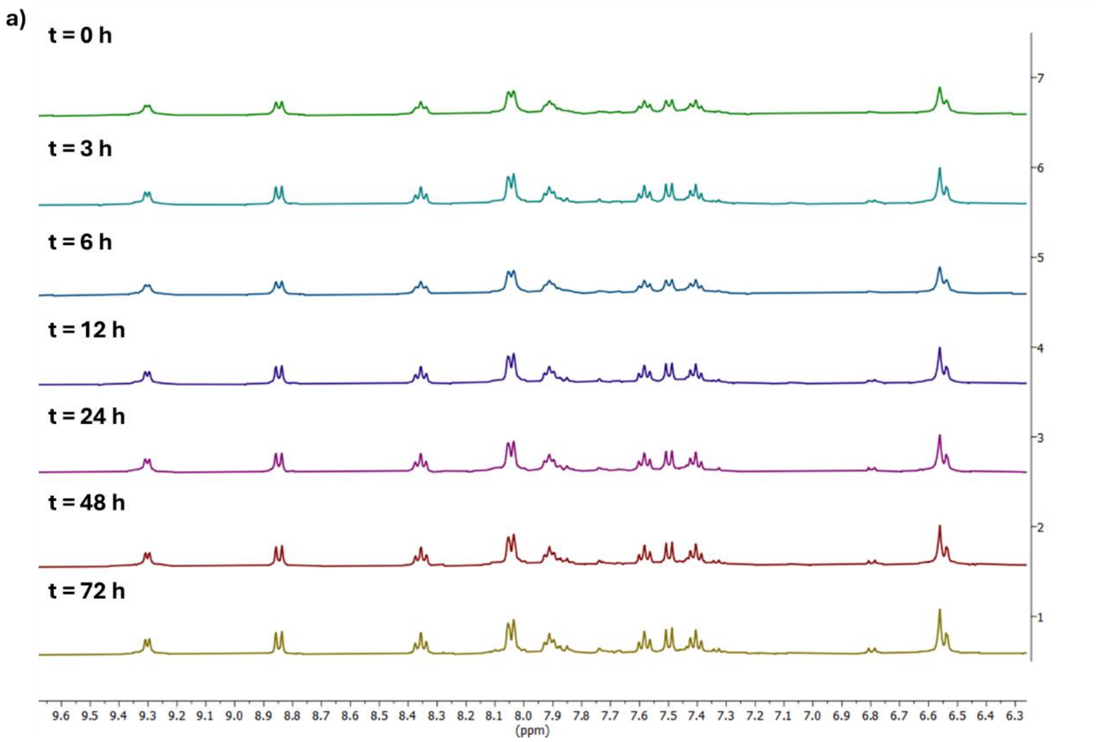

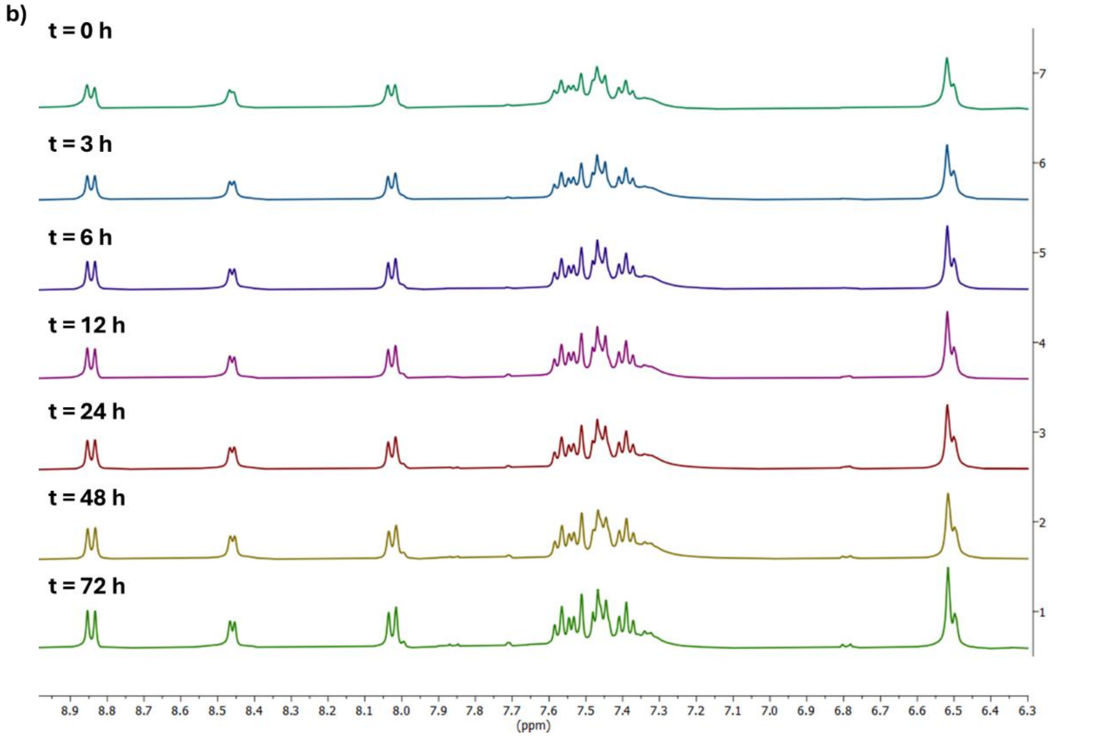

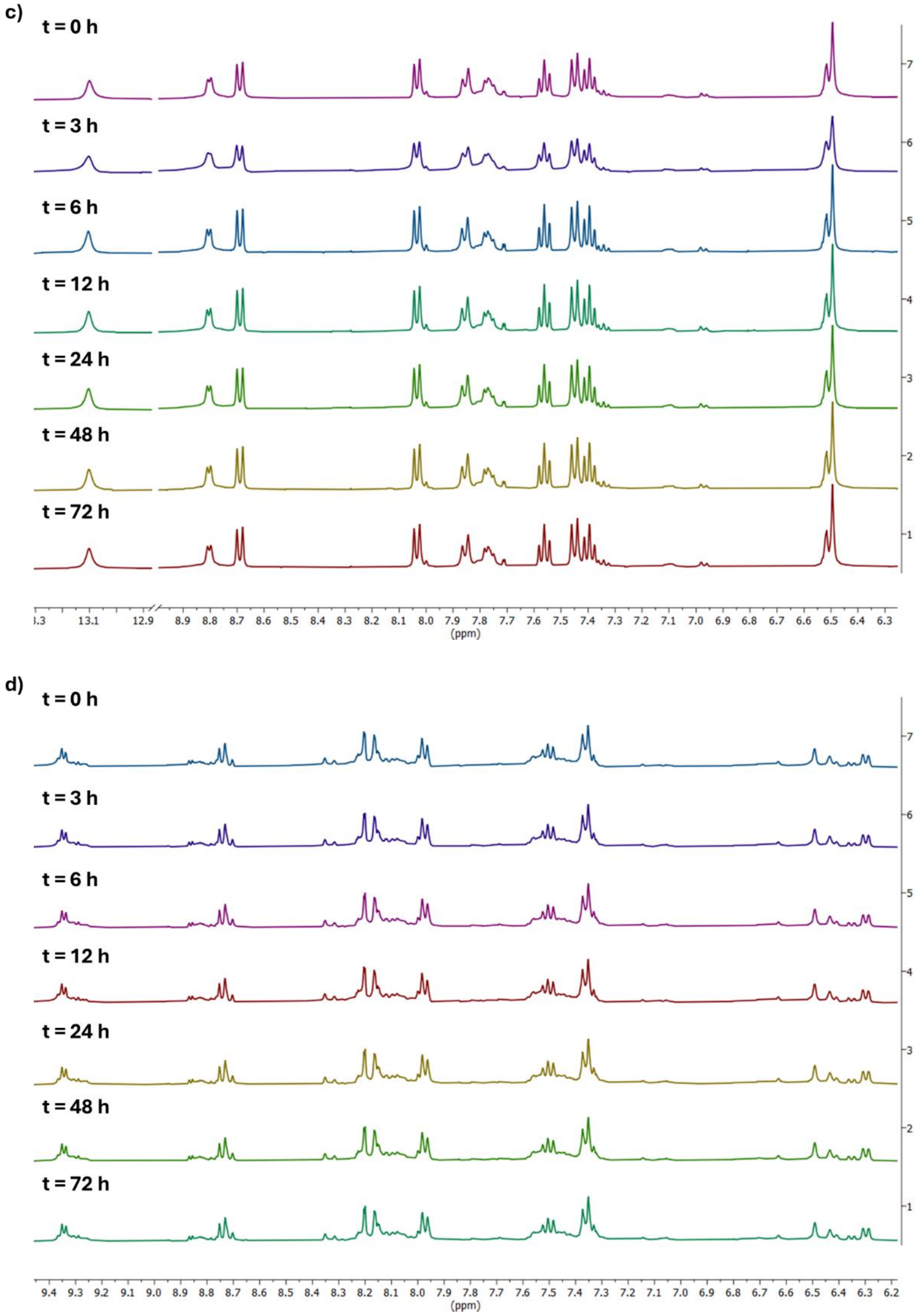

Figure S26. Stability of a) **1**, b) **2**, c) **3** and d) **4** monitored by ^{1}H NMR at several times (0-72 h) recorded in DMSO-d^{6}.

a)

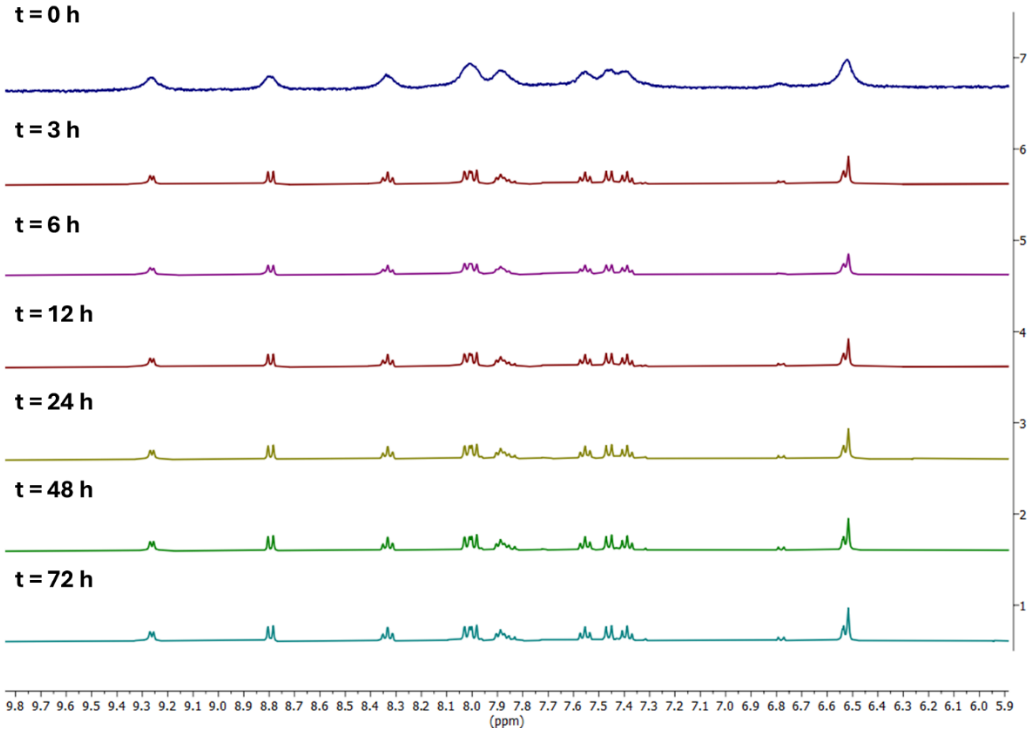
t = 0 h
t = 3 h
t = 6 h
t = 12 h
t = 24 h
t = 48 h
t = 72 h
(ppm)

b)

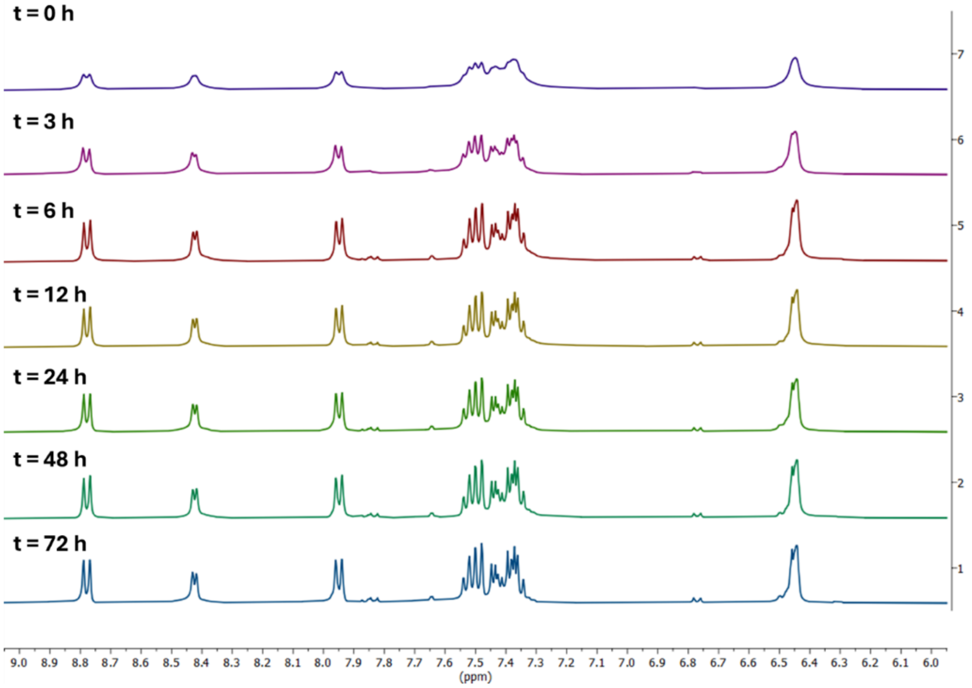
t = 0 h
t = 3 h
t = 6 h
t = 12 h
t = 24 h
t = 48 h
t = 72 h
(ppm)

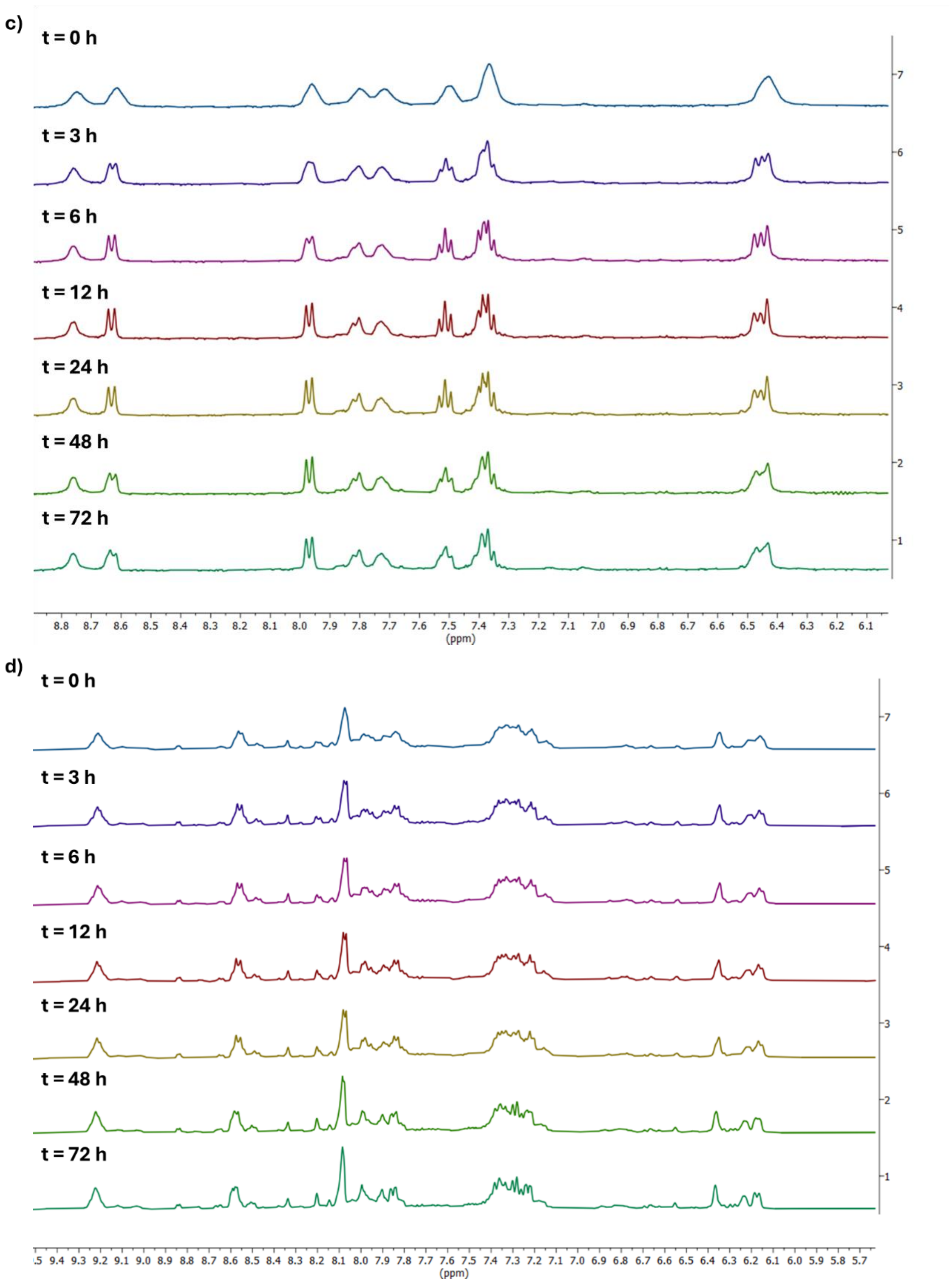

Figure S27. Stability of a) **1**, b) **2**, c) **3** and d) **4** monitored by ^{1}H NMR at several times (0-72 h) recorded in DMSO-d^{6}/D_2O (9/1).

a)

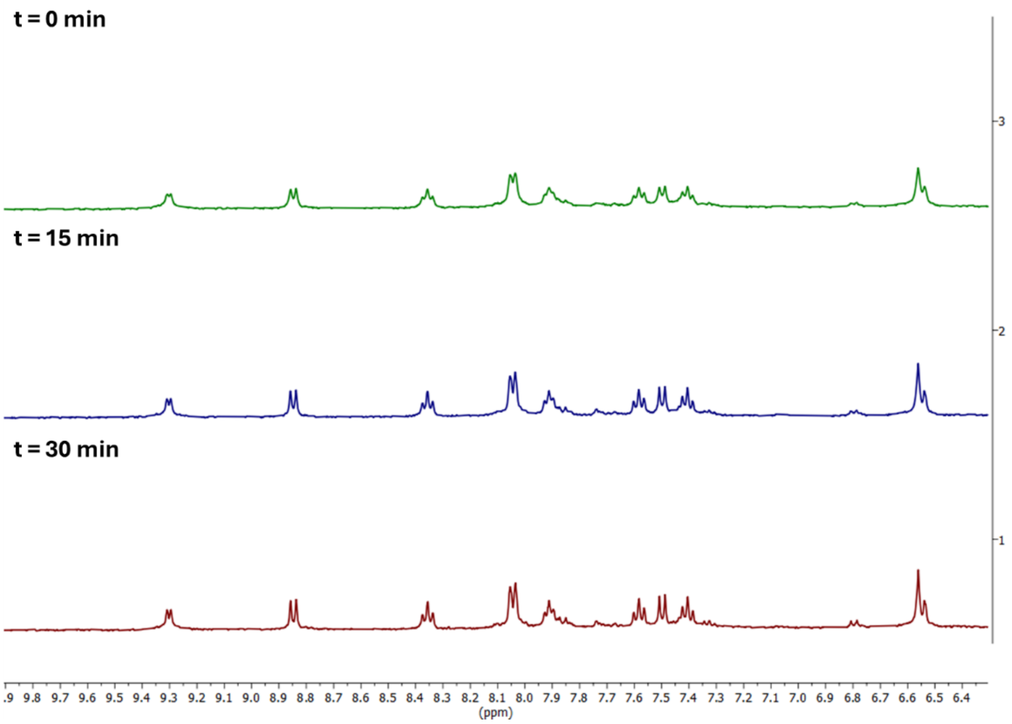

b)

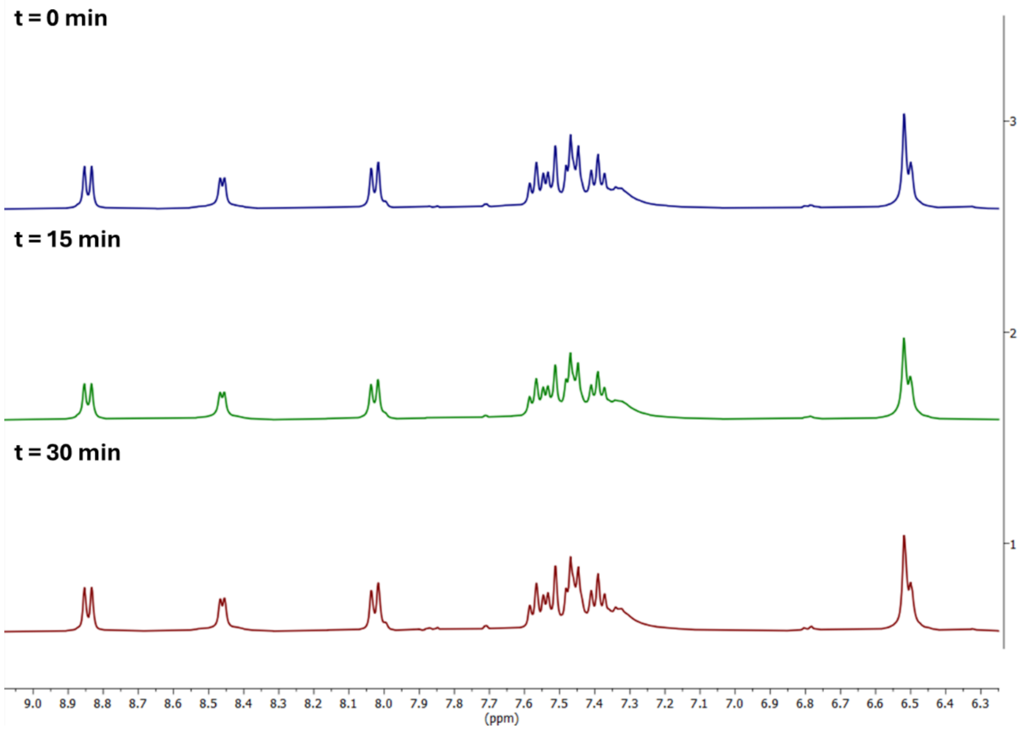

c)

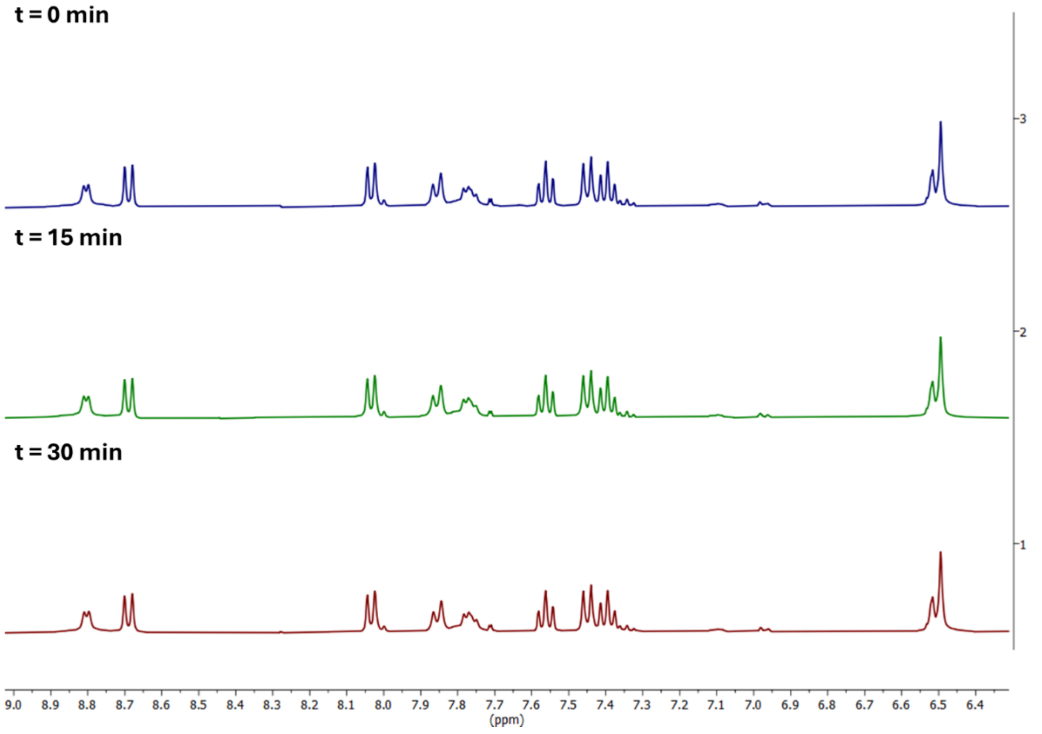

d)

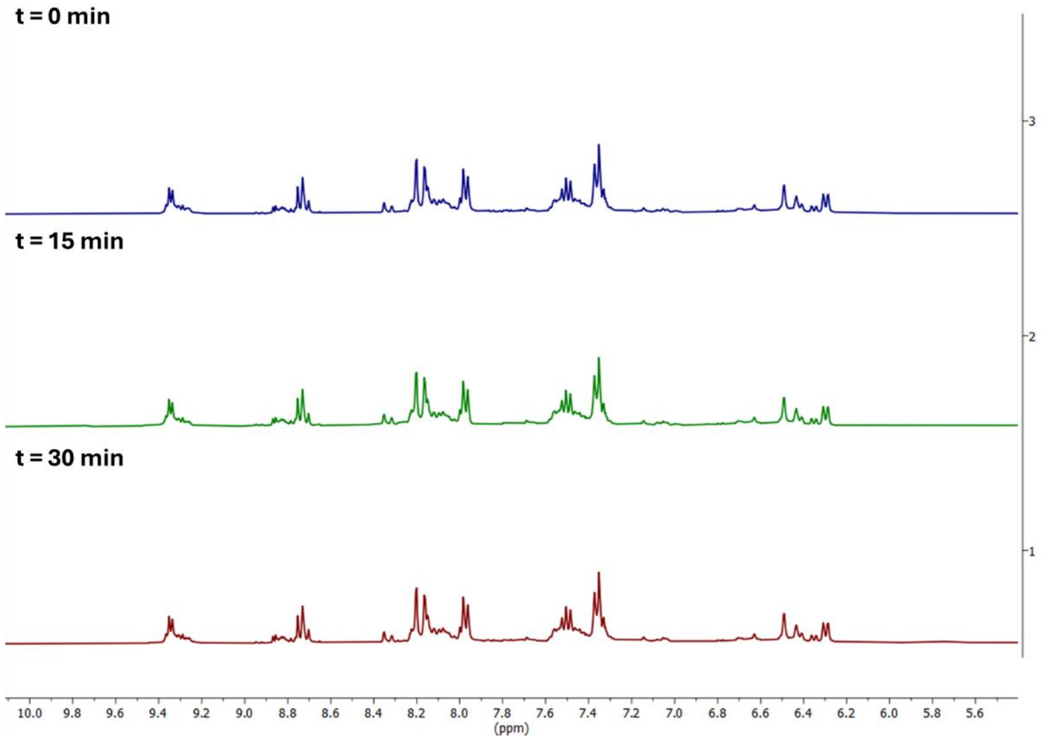

Figure S28. Photostability of a) **1**, b) **2**, c) **3** and d) **4** monitored by ^{1}H NMR at several times of irradiation with 460 nm LED (0-30 min) recorded in DMSO-d^{6}.

a)

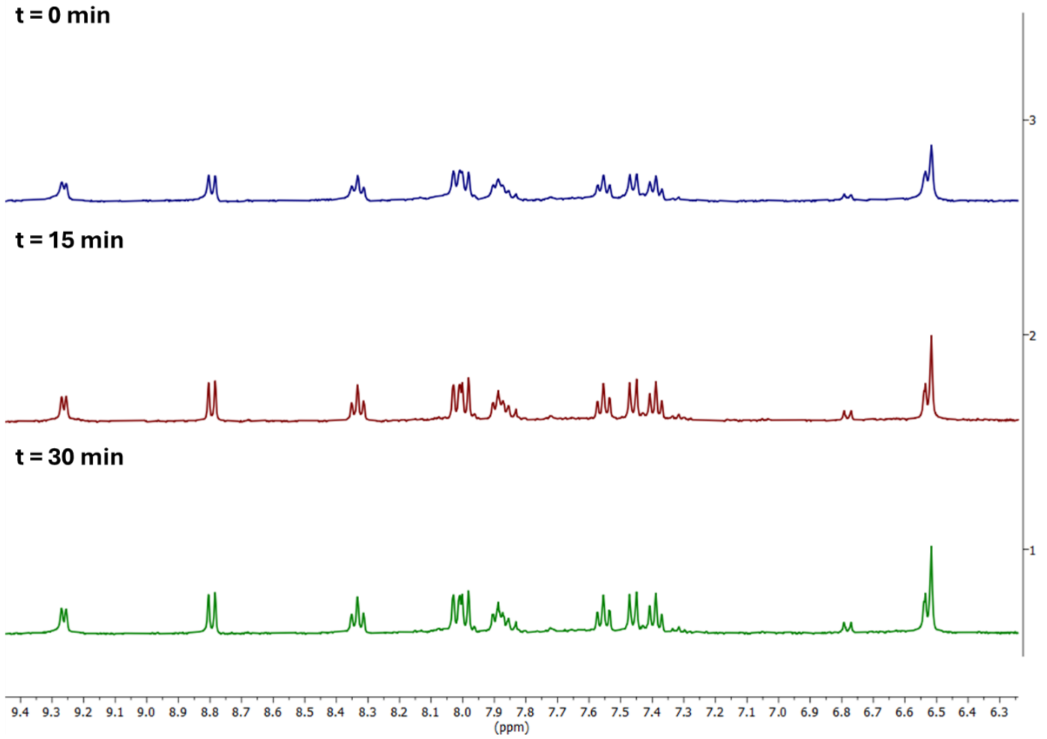

t = 0 min
t = 15 min
t = 30 min
9.4 9.3 9.2 9.1 9.0 8.9 8.8 8.7 8.6 8.5 8.4 8.3 8.2 8.1 8.0 7.9 7.8 7.7 7.6 7.5 7.4 7.3 7.2 7.1 7.0 6.9 6.8 6.7 6.6 6.5 6.4 6.3
(ppm)
3
2
1

b)

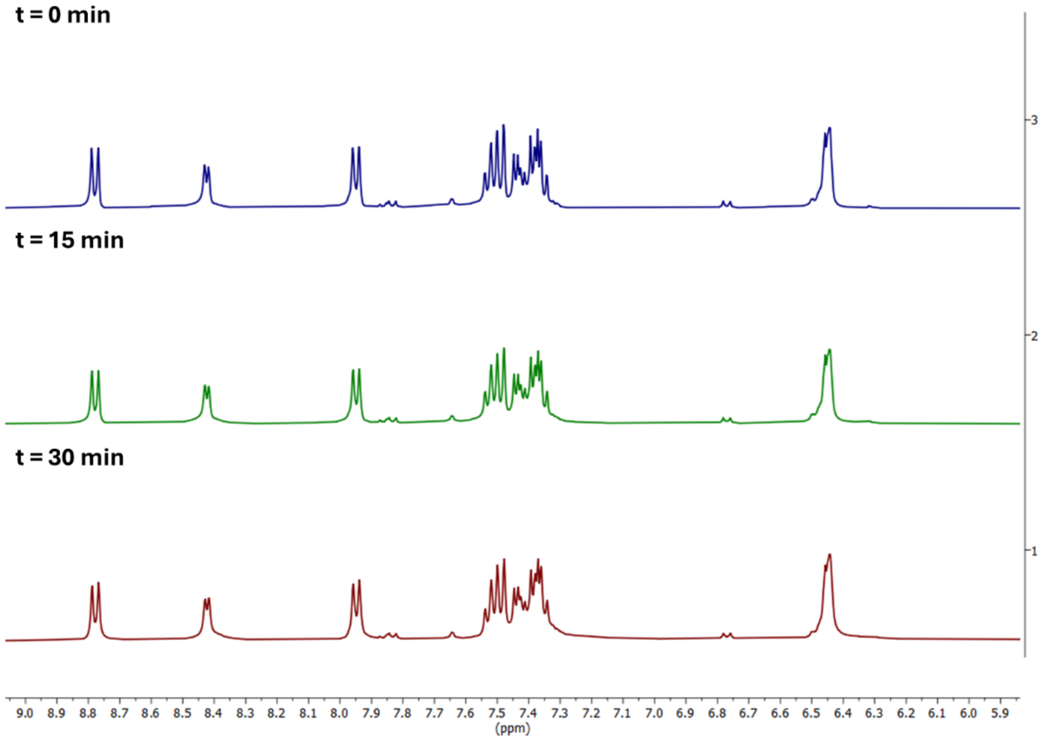

t = 0 min
t = 15 min
t = 30 min
9.0 8.9 8.8 8.7 8.6 8.5 8.4 8.3 8.2 8.1 8.0 7.9 7.8 7.7 7.6 7.5 7.4 7.3 7.2 7.1 7.0 6.9 6.8 6.7 6.6 6.5 6.4 6.3 6.2 6.1 6.0 5.9
(ppm)
3
2
1

c)

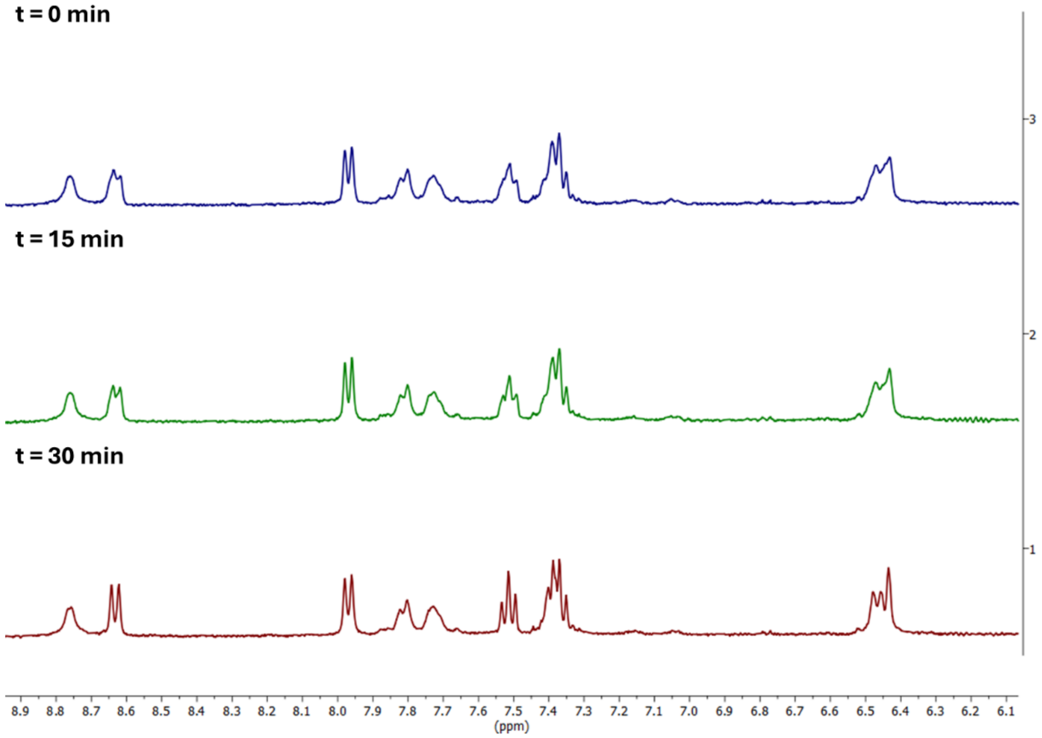

d)

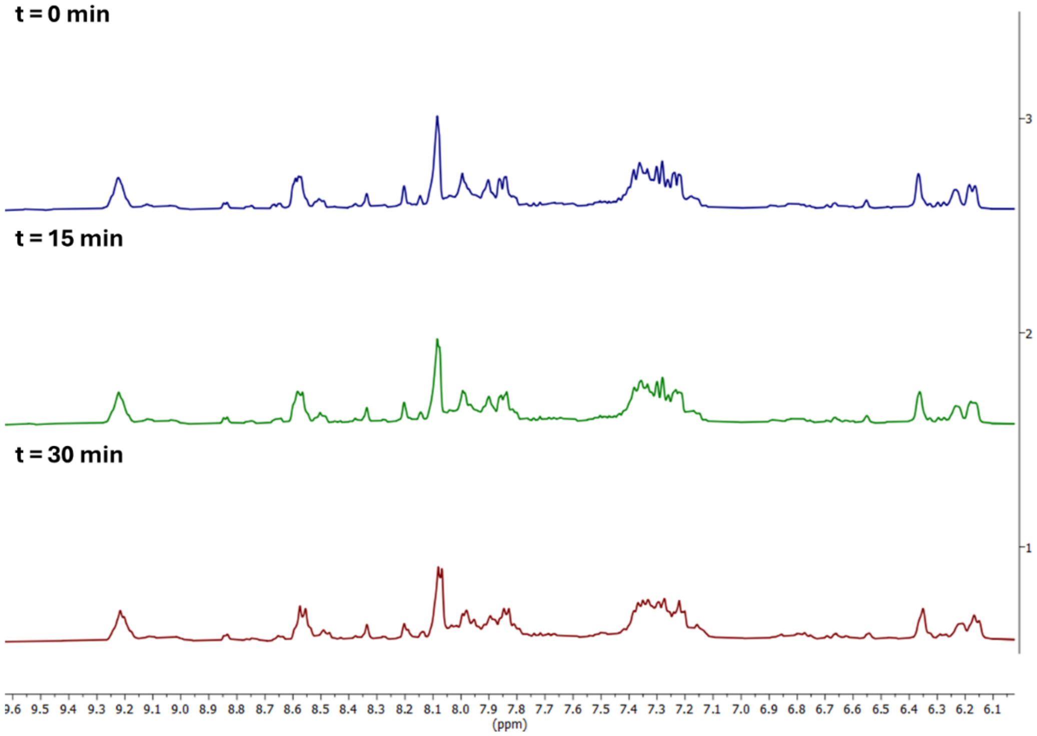

Figure S29. Photostability of a) **1**, b) **2**, c) **3** and d) **4** monitored by ^{1}H NMR at several times of irradiation with 460 nm LED (0-30 min) recorded in DMSO-d^6/D_2O (9/1).

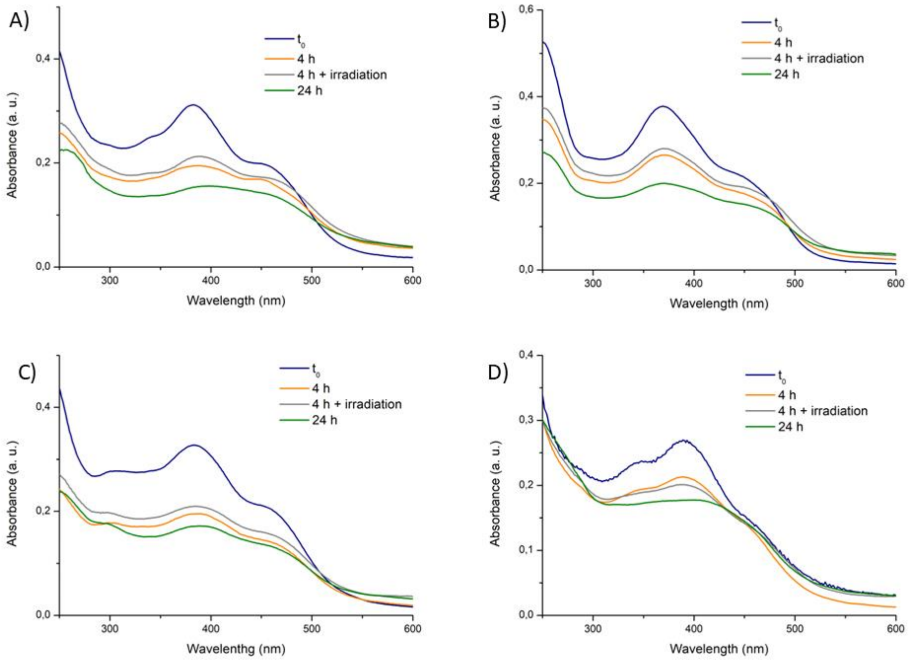

Figure S30. Electronic absorption spectra of complex **1** (A), **2** (B), **3** (C) and **4** (D) in PBS containing 5 % DMSO (25 µM) at t_0, after incubation at 37 °C for 4 h and 24 h in the dark and after incubation at 37 °C for 4 h followed by irradiation 5 min at 450 nm.

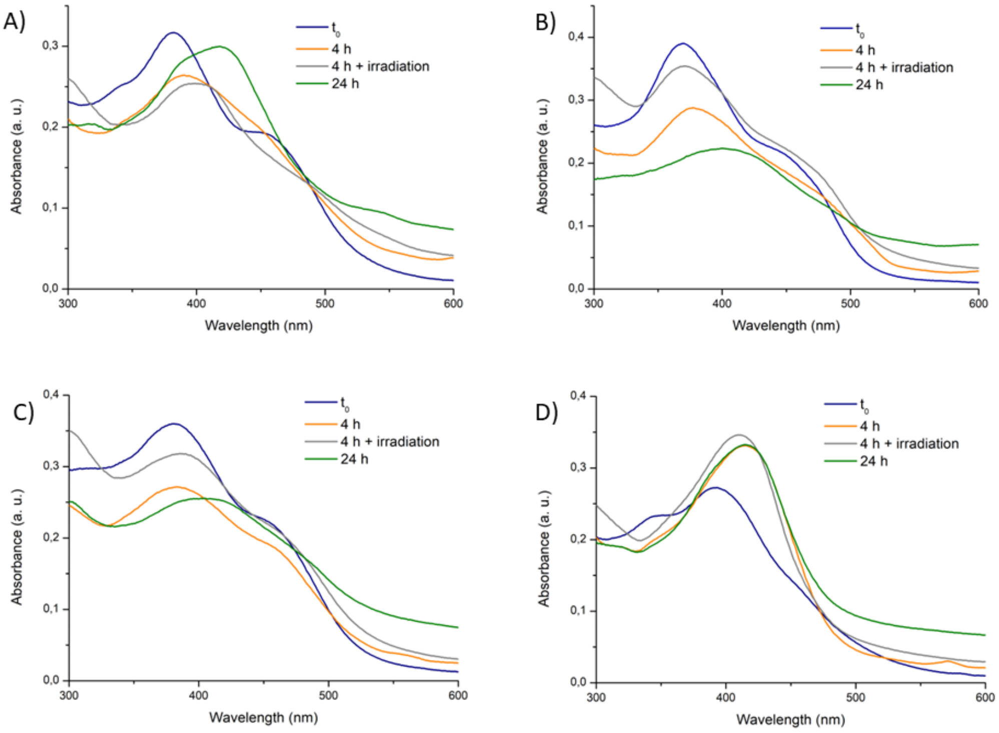

Figure S31. Electronic absorption spectra of complex **1** (A), **2** (B), **3** (C) and **4** (D) in DMEM (w/o red phenol, w/o glutamine, w/o FBS) containing 5 % DMSO (25 µM) at t_0, after incubation at 37 °C for 4 h and 24 h in the dark and after incubation at 37 °C for 4 h followed by irradiation 5 min at 450 nm.

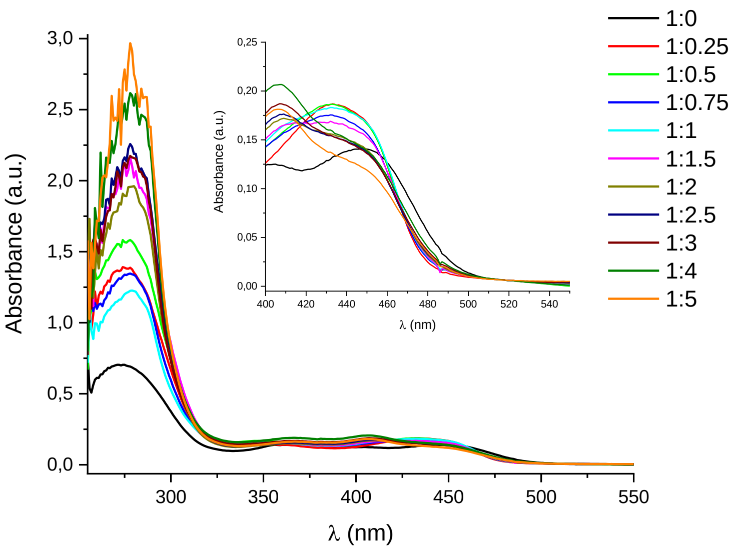

Figure S32. Electronic absorption spectra of complex **3** in DMSO (10 µM) at 298 K with increased amounts of BSA (from 0 to 50 µM).

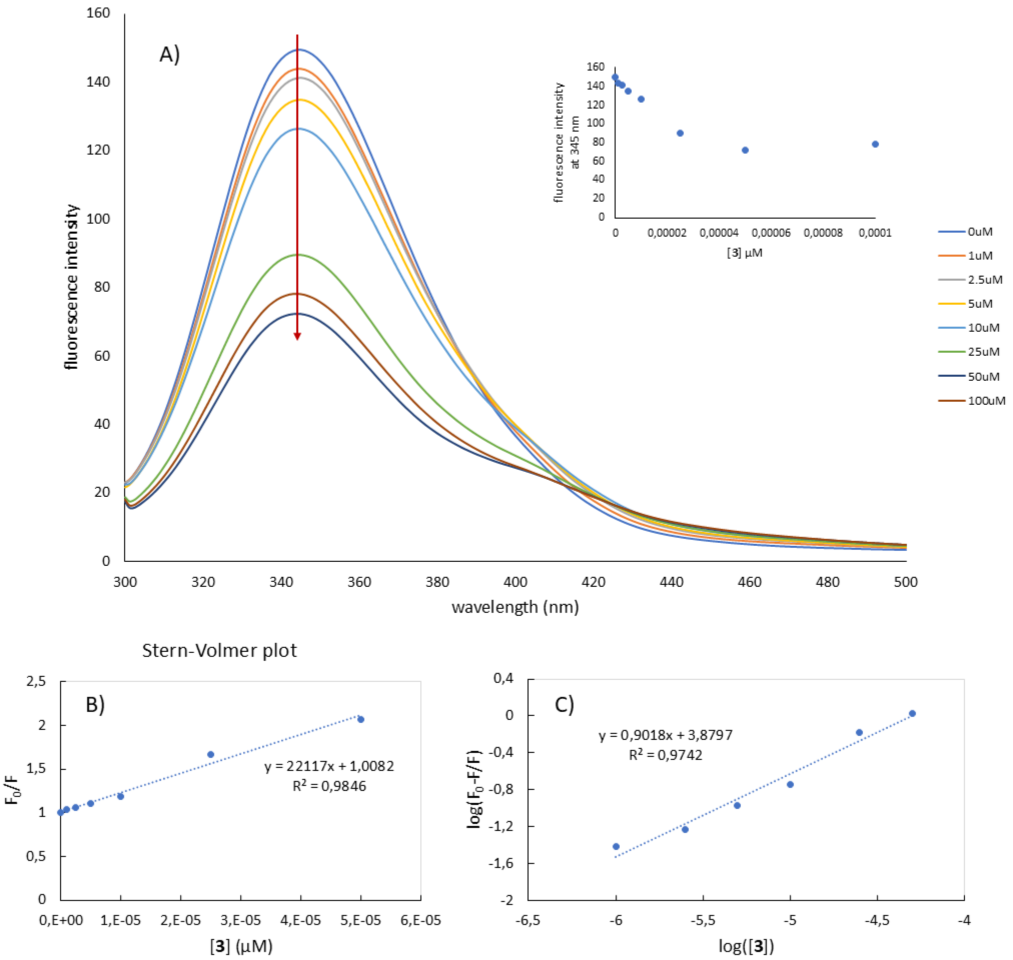

Figure S33. A) Fluorescence spectra (λ_{exc} = 290 nm) of 5 µM solution of BSA in 20 mM PB pH 7.4 with 1 % DMSO after 1 h incubation at room temperature with increasing concentrations of **3** (1 to 100 µM); inset: fluorescence intensity at 345 nm vs. concentration of **3**. B) Stern-Volmer plot showing the quenching of BSA fluorescence by **3**. C) Plot of log(F_0-F/F) vs. log([**3**]).

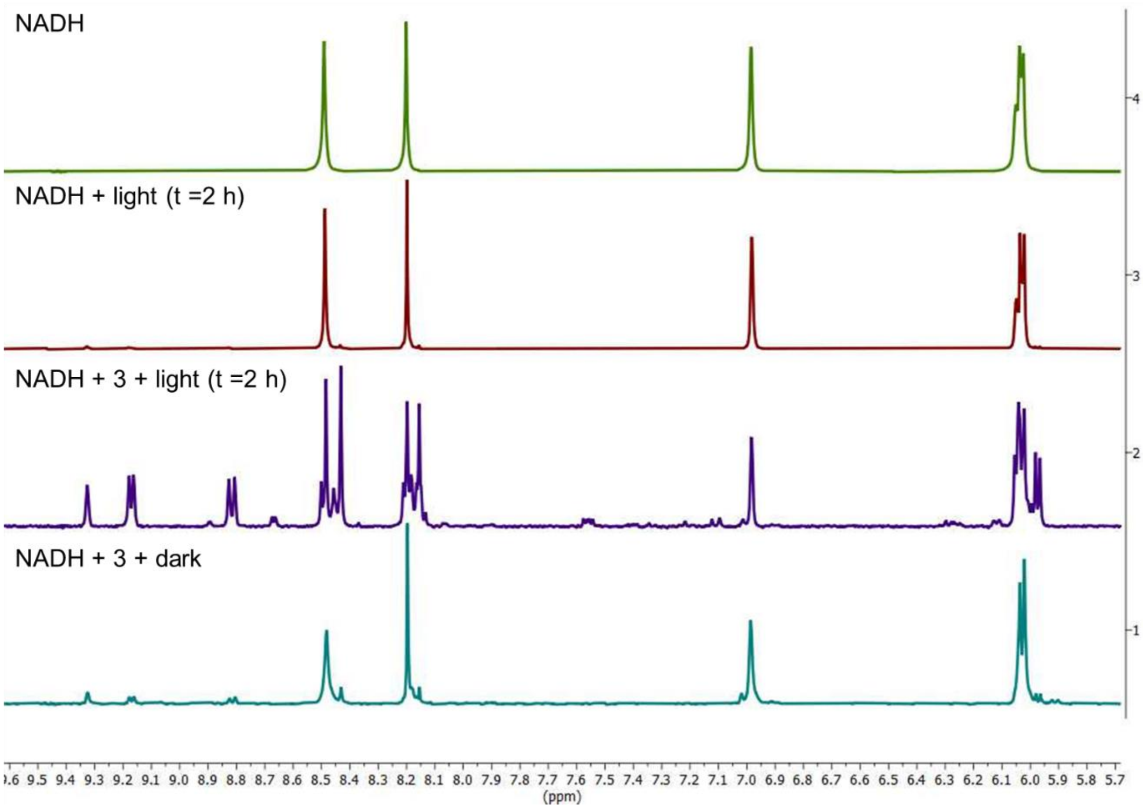

Figure S34. ^{1}H NMR spectra of the oxidation and photooxidation of NADH (2.8 mM) by complex **3** (0.8 mM) in DMSO/D_2O (5/95 v/v) after 2 h light irradiation (460 nm) or leave it in the dark for 72 h.

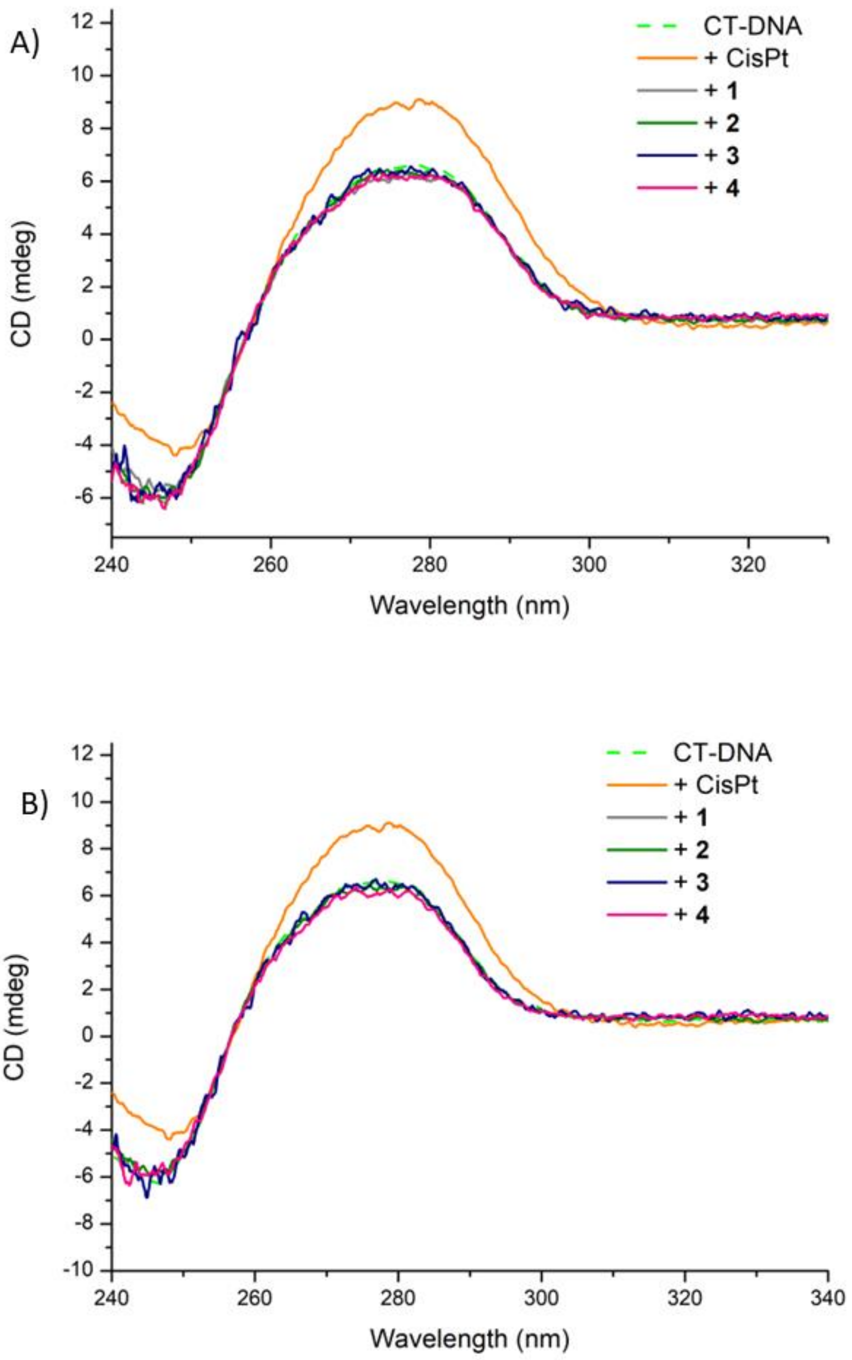

Figure S35. CD spectra of CT-DNA (50 µM) incubated at 37 °C in 5 mM TRIS.HCl, 50 mM NaCl, pH 7.4 with cisplatin and complexes **1-4** A) after 24 h in the dark; B) after 4 h in the dark + 5 min irradiation at 450 nm + 20 h back in the dark.

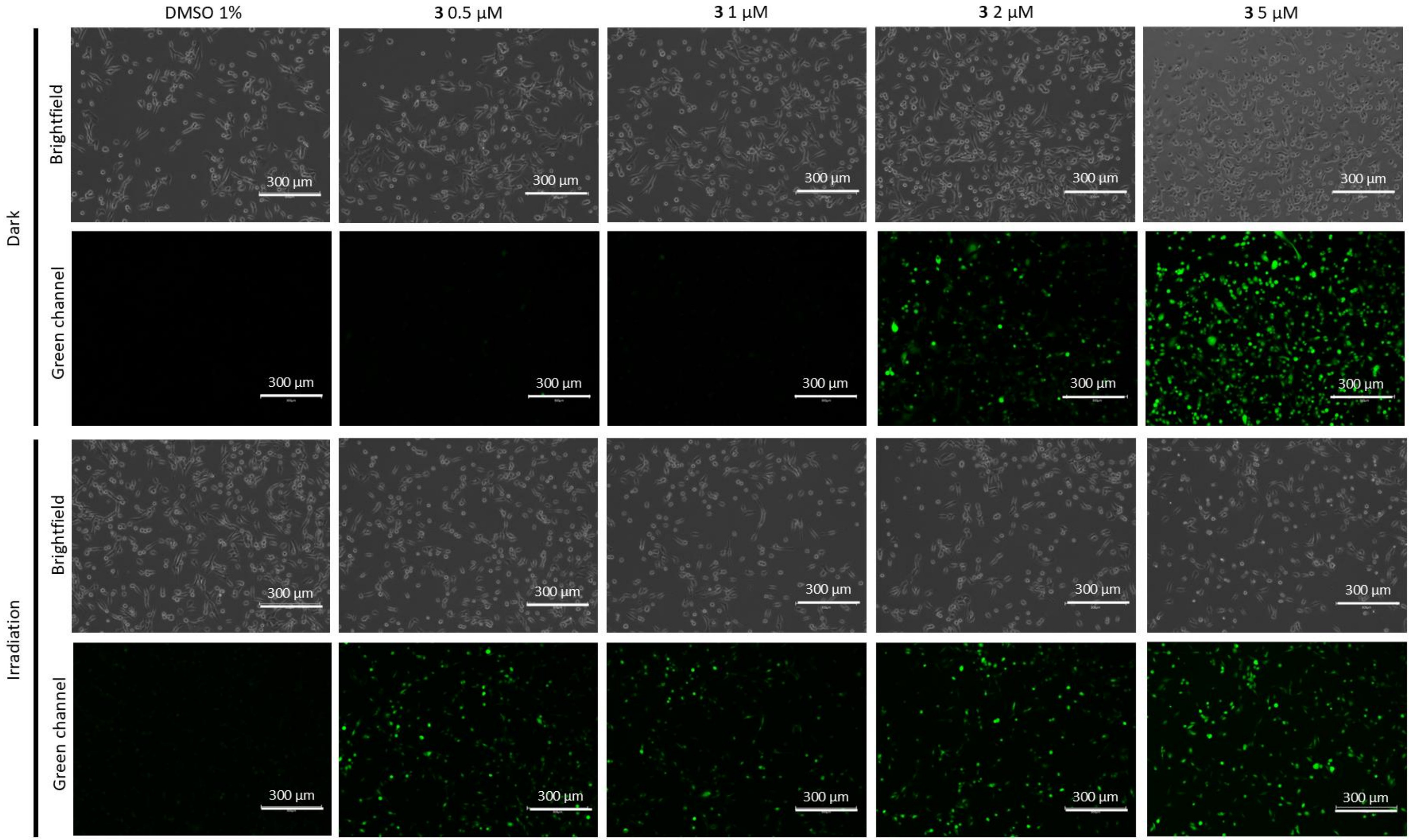

Figure S36. Microscopy images (10x magnification) of MDA-MB-231 cells treated by complex **3** at 0, 0.5, 1, 2 or 5 µM after 4 h incubation at 37 °C in dark conditions (dark) and 4 h at 37 °C of incubation followed by 5 min irradiation at 450 nm (irradiation) in brightfield (cell visualization) and green channel (ROS probe H_2DCFDA fluorescence).
